# Electrochemical recycling of homogeneous catalysts

**DOI:** 10.1126/sciadv.ade3094

**Published:** 2022-10-19

**Authors:** Stephen Cotty, Jemin Jeon, Johannes Elbert, Vijaya Sundar Jeyaraj, Alexander V. Mironenko, Xiao Su

**Affiliations:** Department of Chemical and Biomolecular Engineering, University of Illinois Urbana-Champaign, 600 S Mathews Ave., Urbana, IL 61801, USA.

## Abstract

Homogeneous catalysts have rapid kinetics and keen reaction selectivity. However, their widespread use for industrial catalysis has remained limited because of challenges in reusability. Here, we propose a redox-mediated electrochemical approach for catalyst recycling using metallopolymer-functionalized electrodes for binding and release. The redox platform was investigated for the separation of key platinum and palladium homogeneous catalysts used in organic synthesis and industrial chemical manufacturing. Noble metal catalysts for hydrosilylation, silane etherification, Suzuki cross-coupling, and Wacker oxidation were recycled electrochemically. The redox electrodes demonstrated high sorption uptake for platinum-based catalysts (*Q*_max_ up to 200 milligrams of platinum per gram of adsorbent) from product mixtures, with up to 99.5% recovery, while retaining full catalytic activity over multiple cycles. The combination of mechanistic studies and electronic structure calculations indicate that selective interactions with anionic intermediates during the catalytic cycle played a key role in the separations. Last, continuous flow cell studies support the scalability and favorable technoeconomics of electrochemical recycling.

## Teaser

Selective electrochemical separations can enable the recycling of valuable homogeneous catalysts for key industrial reactions.

## INTRODUCTION

Homogeneous catalysts are known for their remarkable turnover, selectivity, and versatility, making them the systems of choice for a range of important reactions (*1*–*3*). Single-site homogeneous catalysts enable precise control over reaction pathways through synthetic design (*4*–*6*), for key reactions in chemical and pharmaceutical manufacturing (*7*). However, heterogeneous catalysis makes up roughly 75% of chemical and petrochemical processes in industry (*8*). Catalyst homogeneity can be a double-edged sword; while single-site molecular catalysts show superior kinetics and selectivity, separating these catalysts from the product mixtures can be challenging, posing substantial challenges for economical reuse. Moreover, homogeneous catalysts often consist of platinum group metals (PGMs), which are valuable critical elements (*9*).

For example, platinum catalyzed hydrosilylation is a cornerstone for the organosilicon industry (*10*), with a market size valued at $1.1 billion USD in 2019. The recovery of homogeneous platinum catalysts from silane product mixtures is a prominent challenge in chemical manufacturing (*9*–*12*), with the platinum from catalysts accounting for up to 30% of the production cost of silicones. The high viscosity and boiling points of hydrosilylation products make traditional recovery methods such as distillation extremely costly (*9*, *10*). While there have been efforts to find earth-abundant alternatives such as Fe, Co, and Ni complexes (*13*–*16*), platinum-based hydrosilylation catalysts still monopolize the industry due to their unmatched atomic efficiency and kinetics, despite the drawbacks (*9*, *12*). Similarly, palladium-catalyzed cross-coupling reactions have rapidly seen the leap from laboratory-scale organic chemistry to industrial synthesis (*17*–*19*). Recovery of dilute palladium catalysts [<20 parts per million (ppm)] remains a central problem due to excess competing salts (base and stabilizers) in the product solution (*2*). Furthermore, these Pd catalysts contain sensitive ligands, making nondestructive recovery an arduous challenge (*20*).

Therefore, enabling efficient homogeneous catalyst recycling is central for increasing feasibility of these highly active and tunable homogeneous catalysts (*2*). Current catalyst recovery techniques based on thermal or chemical methods can be energetically demanding or require laborious downstream processing (*20*). Distillation is the most common catalyst recovery technique in industry currently (*21*). However, distillation has a limited scope of economic feasibility for reaction systems with low boiling point products and thermally stable catalysts (*20*), making them inefficient for the sensitive Pt and Pd homogeneous catalysts (*20*, *22*). Furthermore, homogeneous catalysis typically operate in the parts-per-million range for catalyst concentration (*23*) due to high turnover numbers and the high cost of the catalysts, making thermal recovery methods even more costly and carbon intensive (*24*).

Electrification of chemical manufacturing offers a key pathway toward carbon neutrality (*25*), and electrochemical separations can play a critical role in this mission (*26*). “Plug and play” electrochemical platforms can lower chemical and energy costs by field-assisted control and facilitate integration with renewable energy (*27*). However, major electrochemical separation processes such as capacitive deionization (*28*) and electrodeposition (*29*) have substantial challenges for fine process synthesis purification, due to a lack of molecular selectivity and dependence on high voltages. On the other hand, redox-active polymers have gained intense attention as a promising platform for selective ion separations (*30*, *31*). Redox-active materials have longtime been the focus of energy storage (*32*), electrocatalysis (*33*), and electrochemical sensing (*34*) and recently have been explored for electrochemical separations due to their selective molecular interactions and electrochemical reversibility (*35*–*37*). However, most of their applications have been for aqueous phase contaminant removal (*38*), with this powerful concept yet to be applied and generalized for value-added recovery of catalysts from organic solutions.

**Fig. 1.**
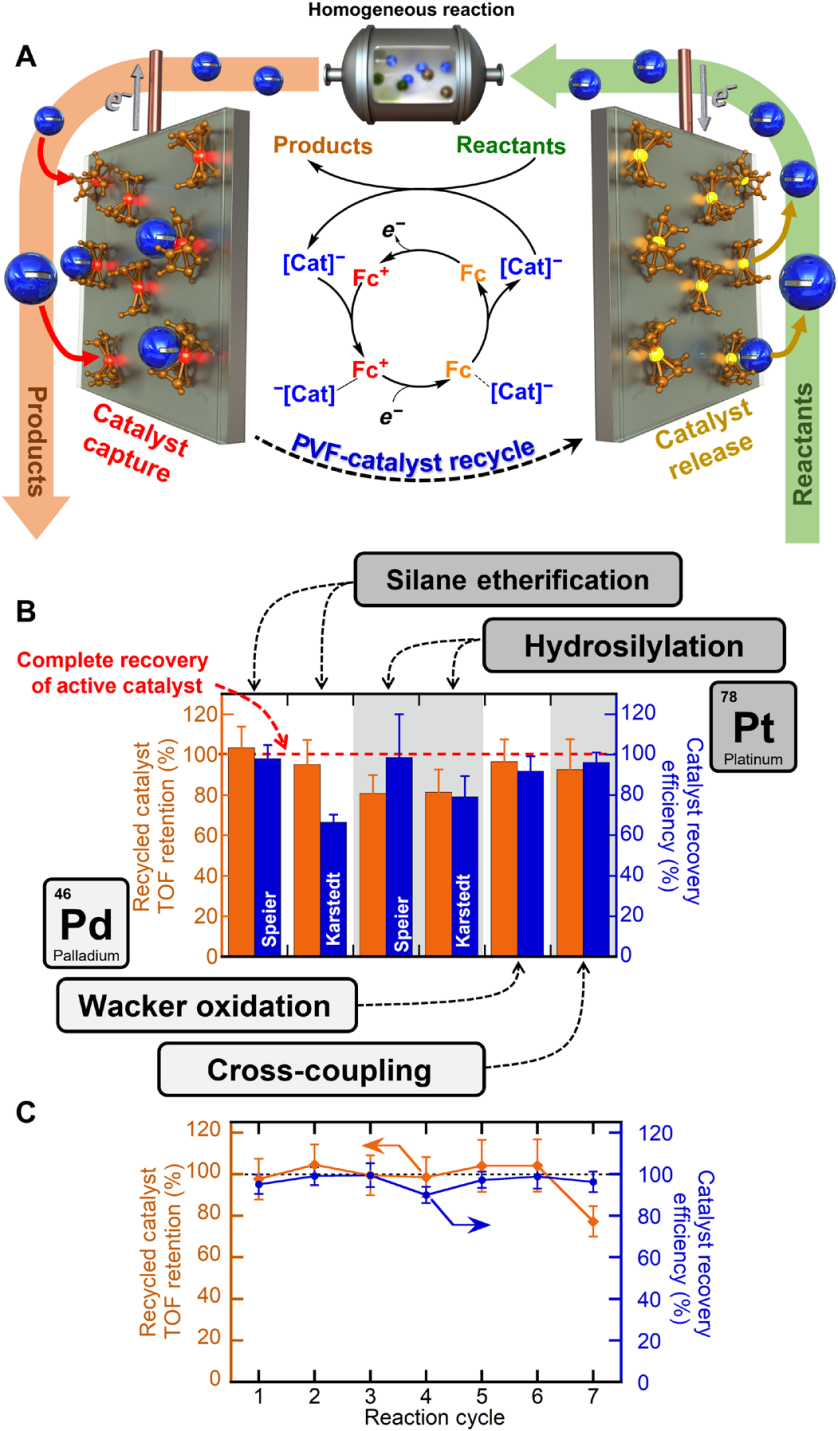
Overall schematic and catalyst recovery performance of the PVF-CNT electrosorption system. (**A**) Redox-mediated electrochemical recycle schematic of organometallic catalysts. (**B**) Comparative catalyst recycle performance of the PVF-CNT electrosorption system for the four model reactions tested, with red dashed line representing full catalyst activity retention. (**C**) Catalyst recycling performance over seven cycles, for silane etherification with the model reaction using ethanol (EtOH) and triethylsilane (TES).

In this work, we develop a benign electrochemical separation platform based on redox metallopolymer–based electrosorbents for the in situ recycling of industrially relevant homogeneous PGM catalysts: Speier’s catalyst (*39*), Karstedt’s catalyst (*16*), palladium chloride (*40*), bis(triphenylphosphine) palladium dichloride (*41*), and other PGM chlorocomplexes. We investigate the performance of our redox separation platform for platinum-catalyzed hydrosilylation and silane etherification, as well as palladium-catalyzed Wacker oxidation and Suzuki cross-coupling (Fig. 1A). Our proof of concept leverages the favorable charge-transfer interactions between the oxidized redox groups with anionic metal complexes for both the charged catalysts and charged catalytic intermediates. Our work demonstrates the powerful generality of our proposed electrochemical recycling concept toward both industrially relevant reactions and major classes of metal-catalyzed organic synthesis. Central to the study is the preservation of catalytic structure and activity, driven solely by electrochemical control without Faradaic destruction of the catalysts. Electronic structure calculations and spectroscopy provide insights into the electrostatic and charge-transfer binding modes between the catalyst and redox electrosorbent. Last, we discuss the technoeconomic advantages of electrochemical homogeneous catalyst recycling and the scalability of the process in a continuous flow process.

## RESULTS AND DISCUSSION

### Redox-mediated electrosorption for reversible catalyst recycling

Polyvinyl ferrocene (PVF)–coated electrode material was selected as the electrosorbent platform due to its rapid charge-transfer affinity for metal-containing anions (*36*). Figure 1A depicts the proposed in situ catalyst recovery scheme: after reaction, the anionic PGM-based homogeneous catalyst is selectively captured from the reaction products with PVF-coated electrodes via an applied oxidizing potential. Neutral ferrocene polymeric units are oxidized to cationic ferrocenium to form favorable binding sites for anionic PGM catalyst, reversibly sequestering catalyst at the electrode surface. Following electrosorption, the catalyst-laden PVF electrode is transferred to a fresh reactant stream, where an opposing applied potential reduces ferrocenium (Fc^+^) back to neutral ferrocene (Fc), deactivating the binding sites and facilitating the release of catalytically active homogeneous complexes into fresh reactants, to restart the reaction with the recycled catalyst. Catalyst recovery is then repeated with the regenerated PVF electrodes, with the entire cycle driven solely by electrical input.

**Fig. 2.**
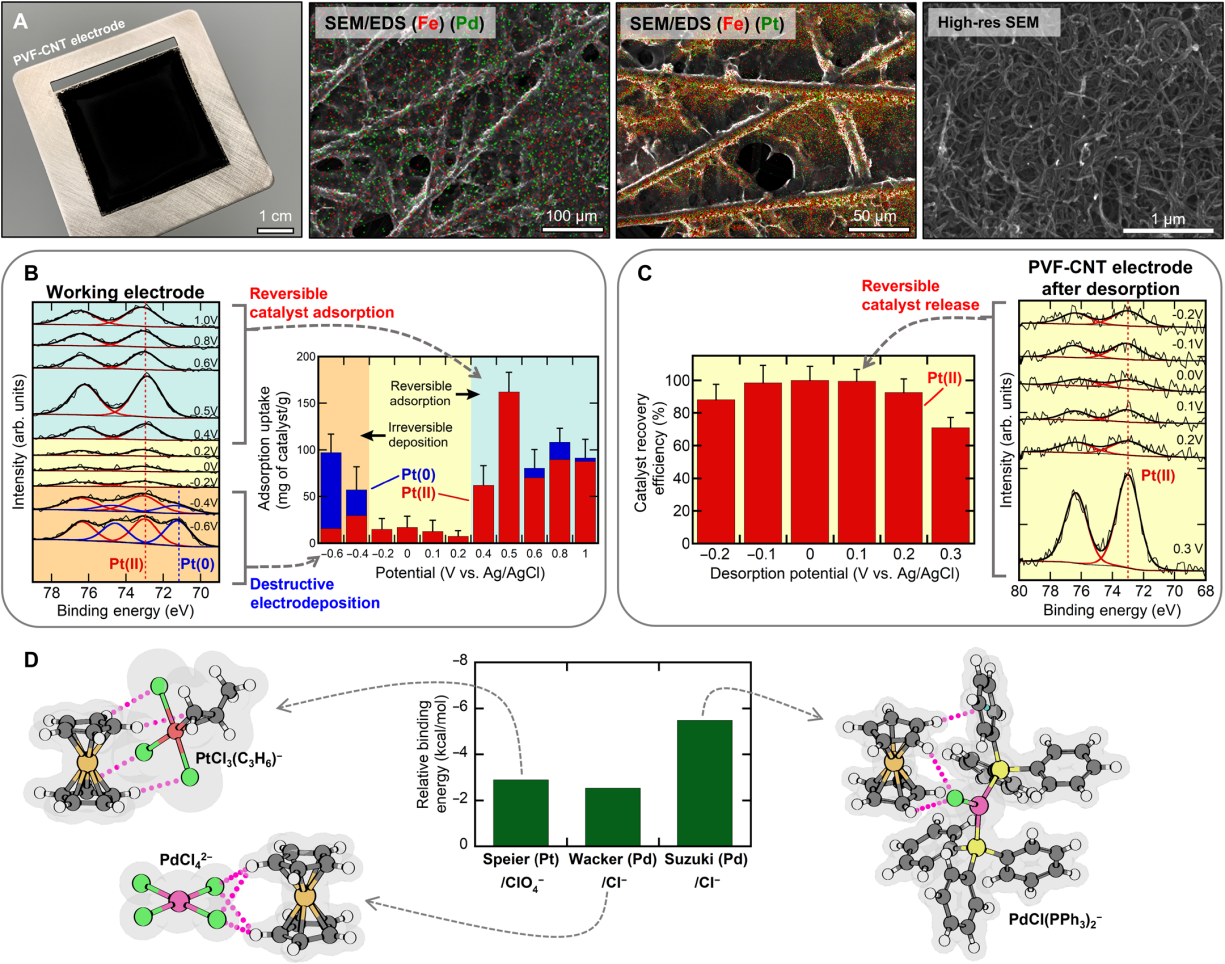
Mechanistic investigation of catalyst binding with electrochemical studies and DFT calculation. (**A**) Left: A photo of a PVF-CNT electrode. Middle: SEM/EDS spectra of PVF-CNT electrodes following adsorption of Pt and Pd catalyst [with Fe (red) and Pd/Pt (green)]. Right: High-resolution SEM of PVF-CNT electrode. (**B**) Left: Pt 4f XPS spectra of PVF-CNT electrodes after adsorption of Speier’s catalyst for a range of applied potentials. Right: Corresponding uptake of Speier’s catalyst for a range of potentials and representative Pt speciation from XPS. (**C**) Left: Regeneration efficiency of Speier’s catalyst over a range of reduction potentials. Speier’s catalyst was initially adsorbed at 0.5 V versus Ag/AgCl. Right: Corresponding Pt 4f XPS spectra of the PVF-CNT electrodes after desorption. (**D**) Optimized geometries of the binding of ferrocenium to Speier’s catalyst [PtCl_3_C_3_H_6_]^−^, the catalyst for Wacker’s reaction [PdCl_4_]^2−^, and the catalyst for Suzuki coupling [PdCl(PPh_3_)_2_]^−^. Interatomic distances are shown in angstroms. Predicted Gibbs binding energy by density functional theory (DFT) of Speier’s, Wacker, and Suzuki catalysts relative to supporting electrolyte for each reaction system (ClO_4_^−^ for Speier and Cl^−^ for Wacker and Suzuki).

Electrochemical catalyst recycle was achieved with a three-electrode electrochemical cell consisting of a PVF and carbon nanotube (PVF-CNT)–coated working electrode, a carbon paper (CP) counter electrode, and an appropriate reference electrode (Ag/AgCl or Ag/AgNO_3_), unless otherwise stated. Toray 030 carbon paper was chosen as the current collector for both working and counter electrodes due to its superior performance at inhibiting electrodeposition of PGMs compared to other materials tested (316SS, Ti, graphene; fig. S17D), as will be discussed in later sections. Cyclic voltammogram of the PVF-CNT working and bare carbon paper counter cell showed excellent reversible behavior and stability (fig. S18). High-resolution scanning electron microscopy (SEM) showed the nanoporous features of the PVF-CNT films, and energy dispersive x-ray spectroscopy (EDS) confirmed the presence of surface-bound iron (Fig. 2A).

We summarize the applicability of our approach for the recycling for key PGM-based homogeneous catalysts, used for major industrial and organic synthesis reactions: silane etherification, hydrosilylation, Wacker oxidation, and Suzuki cross-coupling (Table 1). On the basis of tailored electrochemical operating conditions, >95% preservation of activity and uptake (>100 mg/g) was achievable for all catalyst-reaction systems studied. We first discuss the realization of electrochemically mediated homogeneous catalyst recycling, through preservation of the catalytic reactivity of important, yet sensitive, organometallic catalysts under a range of industrially relevant reaction conditions. Next, we delve into the electrochemical design principles that show how one can tailor the same redox system for different solvents and target compounds, as well as the associated spectroscopic and computational studies of the mechanisms for selective anionic catalyst capture.

**Table 1.**
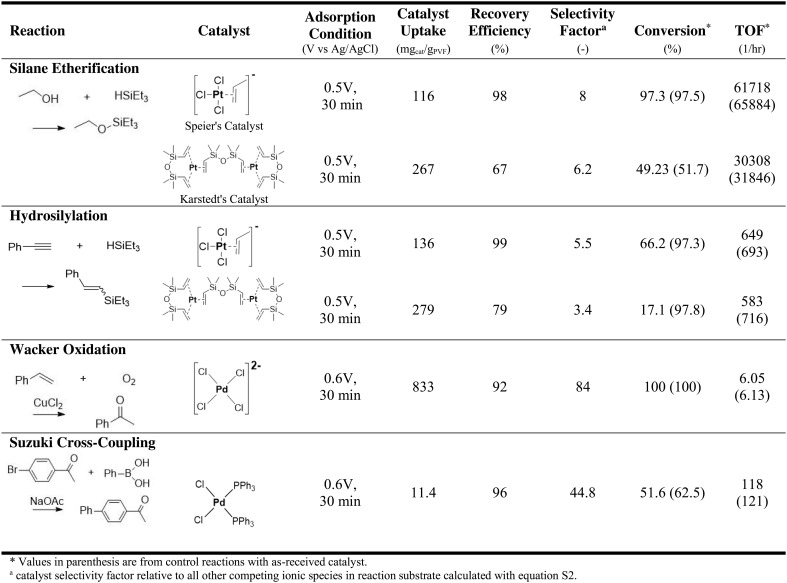
Overview of electrochemically mediated catalyst recycle performance and recycled catalyst reaction metrics for each class of catalyzed reaction.

### Recycling and reuse of homogeneous silane etherification catalyst

The first model reaction investigated for PVF-mediated catalyst recovery was silane etherification with Speier’s catalyst, a staple homogeneous platinum catalyst in the global silane industry due to its high catalytic activity and turnover for a range of hydrosilylation reactions (*10*, *39*). Table 1 depicts the anionic Pt(II) complex formed from hexachloroplatinic acid and isopropyl alcohol (*42*), as well as the reaction schemes, catalyst recycle performance, and operational parameters for the catalyst recycle. Speier’s catalyst was electrochemically recycled from silane etherification products containing 50-ppm catalyst with PVF-CNT electrodes (depicted in the Fig. 1A scheme), yielding a catalyst uptake of 112 mg of Pt/g of PVF from inductively coupled plasma optical emission spectroscopy (ICP-OES) analysis of solution Pt concentration before and after adsorption. To compare, CNT-only electrodes resulted in catalyst uptake (5 mg/g) from silane etherification product solution, directly linking PVF with the high uptake of Speier’s catalyst. In addition, PVF uptake was highly selective to Speier’s catalyst versus competing perchlorate anions in silane etherification medium with a relative selectivity factor of 8.2. Following electrosorption, 98% of Speier’s catalyst was electrochemically released from PVF electrode to fresh silane etherification reactants, and the electrochemically recycled catalyst converted 97.3% of triethylsilane to triethylethoxysilane with an average turnover frequency of 1029 min^−1^, indicating that catalyst activity was retained after electrochemical recycle of Speier’s catalyst with the PVF-CNT system (reactions analyzed with electrospray ionization mass spectrometry, ^1^H nuclear magnetic resonance, and liquid chromatography mass spectrometry).

The kinetic profile of silane etherification (fig. S24A) with electrochemically recycled Speier’s catalyst was compared to a control, catalyzed with 20 ppm of fresh catalyst, and 96 ± 6% of the catalyst activity was retained after recycling (Table 1). In addition, 92% of the isolated product yield was retained compared to the control reaction, indicating that no degradation of reactants or products occurred. For both the recycled and as-received catalyst reactions, a lag period was observed within 5 min, followed by rapid progression to 100% conversion of triethylsilane within 15 min. Notably, the recycled catalyst reaction showed a longer lag period due to the gradual in situ release of the catalyst from the electrode, which was observed to require 4 min on average (fig. S27). Time-of-flight secondary ion mass spectrometry (TOF-SIMS) analysis of the PVF-CNT electrode surface after catalyst adsorption (fig. S65) showed PtCl*_n_* fragments indicating the presence of chloride ligands bound to the adsorbed platinum catalyst, whereas analysis of electrodes following desorption showed no presence of platinum (fig. S62). X-ray photoelectron spectroscopy (XPS) analysis of electrodes confirmed that Pt(II) was only found on the PVF-CNT electrode after adsorption (fig. S12A).

To verify that Speier’s catalyst was the only catalytic active species, PVF-CNT electrodes were oxidized and reduced in silane etherification reactant solution devoid of the homogeneous catalyst, and the result showed no conversion (fig. S28). The active platinum catalyst was detected with ultraviolet (UV) spectroscopy in situ during electrochemical release of Speier’s catalyst into silane etherification reactants (fig. S24C). The UV spectra of a control silane etherification reaction (where 50 ppm of fresh Speier’s catalyst was used) showed a distinct peak at 360 nm, assigned to the active catalytic platinum species in accordance with literature (*43*). Comparing recycled catalyst reaction spectra to control, features corresponding to the active catalyst were preserved, thus indicating that reversible capture and release did not affect catalyst structure or activity, allowing for immediate reuse.

Speier’s catalyst was electrochemically recycled in a range of alcohol and silane substrates for silane etherification (fig. S24B), and an average of 95% of recycled catalyst activity was retained for the entire substrate scope. Catalyst uptake over the span of 30 min varied from 52.3 mg/g with isopropanol-triethylsilane to 151.1 mg/g with ethanol (EtOH)–dimethylphenylsilane due to greater solution conductivity, allowing more facile adsorption kinetics. Catalyst recovery efficiency of the PVF-CNT electrode was consistently high regardless of substrate, averaging 97.7% of all adsorbed catalyst released, thus demonstrating the generality of the recycling approach for diverse silane etherification reactions.

#### Electrode cycling

To confirm electrode cyclability, a PVF-CNT electrode was reused for multiple catalyst recovery cycles (Fig. 1C) in the etherification reaction of triethylsilane with EtOH. Speier’s catalyst uptake and regeneration remained consistent throughout all cycles, with an average uptake of 100 mg/g and a regeneration efficiency of 99.7% for seven consecutive cycles of recycling. The recycled catalyst turnover frequency and product yield remained consistent relative to the corresponding control reaction, with an average turnover frequency of 1354 min^−1^, corresponding to a 95 ± 6% retention of catalyst activity upon release. In addition, no Pt(II) or Pt(0) was observed with XPS analysis on reused electrodes after recycling (fig. S12A), confirming elimination of irreversible electrodeposition and avoidance of catalyst degradation. Last, PVF-CNT electrodes were cycled over 5000 charge/discharge cycles (methodology described in the Supplementary Materials), and cyclic voltammograms taken before and after electrode cycling show no loss in electrochemical performance (fig. S5F), supporting the robust longevity of these electrodes in relevant organic solvents.

### Recycling and reuse of homogeneous Pt catalysts for hydrosilylation

The discovery of Speier’s catalyst in 1957 launched the organosilane industry owing to the catalyst’s high activity and turnover for the cornerstone hydrosilylation reaction, and, later, the discovery of Karstedt’s catalyst, derived from Speier’s catalyst, improved catalyst solubility to a wider range of hydrosilylation substrates and propelled the silane industry to the globally encompassing giant recognized today (*10*). As a proof of concept, electrochemical recycle of both Speier’s catalyst and Karstedt’s catalyst was investigated for the homogeneously catalyzed hydrosilylation of triethylsilane and phenylacetylene using PVF-CNT electrosorbent.

#### Speier’s catalyst

Analogous to silane etherification results, PVF-CNT electrosorption achieved a catalyst uptake of 135 mg of Pt/g of PVF from product solution with 98.0% of catalyst electrochemically released into fresh triethylsilane and phenylacetylene reactants (Table 1), indicating successful catalyst recovery with PVF electrosorbent. PVF electrosorption sites were selective to Speier’s catalyst under the hydrosilylation reaction conditions, with a relative selectivity factor of 5.5 versus supporting perchlorate anions based on XPS (fig. S15B). The electrochemically recycled catalyst retained 81 ± 10% of its catalytic activity (649 hour^−1^ of TOF), and the isolated hydrosilylation product yield was similarly within 83% of control (Table 1). No metallic Pt(0) was observed on electrodes after adsorption and desorption with XPS (fig. S12B); furthermore, TOF-SIMS analysis of the PVF-CNT electrode surface after electrosorption (fig. S65) showed PtCl*_n_* fragmentation, indicating the retention of chloride ligands on adsorbed platinum catalyst.

#### Karstedt’s catalyst

Electrochemical recycle of Karstedt’s catalyst from hydrosilylation product solution was also successful with an uptake of 279 mg of Pt/g of PVF with 79.2% of Karstedt’s catalyst recycled into fresh hydrosilylation reactants (Table 1). The presence of Pt on the electrode surface after adsorption was confirmed with TOF-SIMS analysis (fig. S65), and Karstedt’s catalyst showed a selectivity factor of 3.36 versus competing perchlorate anions, with clear accumulation of Pt on Fe adsorption sites (table S20 and fig. S10). The recycled catalyst progressed with a turnover frequency of 583 hour^−1^ for the hydrosilylation reaction, resulting in 81% retention of catalytic activity after recycling.

To delve into the charged state of the active intermediate from Karstedt’s catalyst during reaction, electric conductivity was measured for solutions of the silane etherification reactions catalyzed by a range of concentrations of Karstedt’s catalyst (from 0 mM Pt to 1.3 mM Pt in the presence of reaction substrates) and compared to control solutions of the catalyst in pure EtOH (no reactants). Once Karstedt’s catalyst was added, the reaction progressed, and the solution resistance markedly reduced from 3.5 megohms to 62 kilohms (fig. S23). On the other hand, the addition of Karstedt’s catalyst to pure EtOH showed no change in solution resistance of 3.5 megohms. The molar electrical conductivity of Karstedt’s catalyst was two orders of magnitude more conductive in the silane etherification reaction solution (12.6 μS cm^−1^ mM^−1^) than the catalyst solely in pure EtOH (0.07 μS cm^−1^ mM^−1^). These observations, in addition to the catalyst recycling results, provide evidence that ionic catalytic intermediates were formed during silane etherification, strongly indicating that an anionic catalyst intermediate is likely to be the bound species during separations, despite a neutral starting Karstedt’s precatalyst. These results are in line with observations by Stein *et al.* (*43*) that suggest that platinum converges to a common active catalytic intermediate regardless of initial precatalytic composition. Our results indicate that an anionic intermediate plays a key role in both the Speier and the Karstedt’s cycle.

### Recycling and reuse of homogeneous Pd catalysts for oxidation and cross-coupling

#### Wacker oxidation

Following successful in situ recycle of platinum-based homogeneous catalyst systems, PVF-enabled electrochemical capture and release of industrially pertinent palladium-based homogeneous catalyst systems were investigated, namely PdCl_2_-catalyzed Wacker oxidation, well known to form anionic active catalyst intermediates (*44*). Wacker oxidation is the textbook example of a successfully applied homogeneous catalyst system in industry, and with arguably the most versatile transition metal in catalysis, palladium, the Pd-catalyzed Wacker system was an impactful model reaction to measure PVF-enabled catalyst recycle performance (*45*). Electrochemical capture of PdCl_2_ catalyst from the Wacker oxidation of styrene to acetophenone yielded an uptake of 989 mg of Pd/g of PVF of PdCl_2_ compared to previous platinum recycle results due to the high catalyst concentration, 60 mM PdCl_2_ (Table 1). Despite a 20-fold excess of redox-active catalytic promotor, CuCl_2_ (fig. S25E), PVF remained selective to Pd with a selectivity factor of 84 Pd:Cu from ICP-OES data. To contrast, the absence of PVF (CNT only) inhibited Pd catalyst uptake by 98.7% with no selectivity and confirmed PVF-driven catalyst uptake. Following adsorption, 84% of PVF-captured Pd catalyst was electrochemically released and recycled to fresh Wacker reactants via 0.1 V reducing potential (Fig. 1B), indicative of reversible catalyst recycle. From gas chromatography–mass spectrometry (fig. S68), the electrochemically recycled palladium catalyst retained 93% of its catalytic activity and 98% of isolated product yield (Table 1), demonstrating effective recycle of high concentration palladium complex catalysts without loss of catalytic activity for Wacker oxidation reactions with the PVF system.

#### Suzuki cross-coupling

Similar to PdCl_2_ in Wacker oxidation, the industrial standard Suzuki cross-coupling catalyst, PdCl_2_(PPh_3_)_2_, has been reported to be anionic in the active catalytic state (fig. S26C) (*46*). Further, the Nobel Prize winning Suzuki-Miyaura reaction has quickly become an indispensable tool in the agrochemical and pharmaceutical industry despite notable catalyst recycle challenges, making an excellent candidate system for PVF-enabled catalyst recycle (*47*). In contrast to previously tested systems, retention of sensitive triphenylphosphine ligand structure was critical for effective catalyst reuse (*46*, *48*), and Suzuki reaction substrate typically contained >100 fold excess of competing ionic species compared to catalyst (*48*). Therefore, PVF electrosorption was initially investigated in control solutions of the isolated anionic catalyst intermediate, [PdCl(PPh_3_)_2_]^−^, achieving an uptake of 47.5 mg/g and 86.4% recovery efficiency, and compared to neutral PdCl_2_(PPh_3_)_2_ species, uptake of isolated anionic complex was 78% greater (fig. S26D), illustrating selectivity of ferrocenium binding with the anionic catalyst intermediate. Last, an atomic ratio of 2.5:1 (±0.4) phosphorous to palladium was observed in solution after release with ICP-OES, demonstrating the intact recovery of the ligated palladium catalyst with the PVF system.

In situ recycle of PdCl_2_(PPh_3_)_2_ catalyst in cross coupling reactions with PVF-CNT resulted in catalyst uptake of 11.3 mg/g despite the dilute (18 ppm) catalyst concentrations, whereas no uptake was observed with CNT control (Fig. 1). Both PVF and the reactants of the Suzuki reaction were electrochemically stable over multiple voltammogram cycles with no apparent degradation (fig. S26, A and B), and the selectivity factor of Pd catalyst was 44.8 despite the high ionic strength (>1 M) of Suzuki reaction promotors: sodium acetate and tetrabutylammonium bromide. Electrochemical release of PVF-bound Pd catalyst resulted in a 96.2% recovery efficiency, and the recycled palladium catalyst retained 97% of its catalytic activity with 91% retention of isolated 4-acetylbiphenyl product yield compared to unrecycled control reaction (Table 1), indicating proof of principle of homogeneous recycling. In addition, no palladium was observed with XPS on the PVF-CNT or counter electrode after desorption (fig. S16C), confirming complete catalyst release with no palladium electrodeposition.

### Mechanistic electrochemical studies of Pt and Pd interaction with redox interfaces

For successful homogeneous catalyst recycling to occur with PGM complexes, detailed understanding of the Pt and Pd redox behavior at the surfaces was critical, as well as the role of the associated voltage windows, solvent, and electrolyte. Here, we discuss the fundamental electrochemical and spectroscopic studies with Pt and Pd-chloroanion complexes that are the basis for the reversible electrosorption of homogeneous catalysts, as well as the selection for the operating conditions used to obtain superior capacity and regeneration efficiencies demonstrated above.

#### Platinum catalyst recycle

To distinguish between reversible redox-mediated electrosorption and destructive Faradaic electrodeposition of Pt, batch-scale experiments were performed at a range of chronoamperometric potentials (−0.6 to 1.0 V versus Ag/AgCl). Speier’s catalyst (1 mM) with 20 mM tetrabutylammonium perchlorate (TBAP) in EtOH was used as a model solution, and a constant potential was applied to the PVF//carbon paper (CP) cell for 30 min for electrosorption. The catalyst uptake was measured for each potential using the difference in platinum concentration by ICP-OES before and after electrosorption. In addition, XPS surface analysis of both working and counter electrode identified the oxidation state of captured platinum catalyst, where Pt 4f spectra gave distinct doublet (δ = 3.35 eV) peaks at 73 eV for Pt(II) and 71 eV for Pt(0) species (fig. S11) (*49*, *50*). The results (Fig. 2B) displayed three distinct regions of catalyst uptake: (i) E < −0.4 V in orange, (ii) −0.2 V < E < +0.3 V in yellow, and (iii) E > +0.4 V in blue.

In the blue region of Fig. 2B (>+0.4 V), rapid Pt uptake was observed with a maximum at 0.5 V (162 mg/g). Oxidized ferrocene sites were observed on the working electrode with XPS at potential above 0.4 V, corresponding well with the oxidation potential of PVF from cyclic voltammetry (CV) (fig. S13). Pt 4f spectra of the working electrode surface at >+0.4 V (Fig. 2B) showed 100% Pt(II), indicating PVF adsorption of Speier’s catalyst without alteration of the platinum oxidation state. EDS mapping of the electrode after electrosorption at +0.5 V in Fig. 2A showed clear overlap of Fe-rich regions (from ferrocene) with Pt-rich regions (from Speier’s catalyst), confirming ferrocene-mediated binding. In addition, TOF-SIMS surface analysis of PVF-CNT electrodes after adsorption at +0.5 V showed the presence of PtCl*_n_* fragments, indicating that the electrosorbed Pt(II) species retained its chloride ligands (figs. S63 and S65). In the absence of a PVF-CNT coating, no Pt uptake was observed at potential above +0.4 V (fig. S17D); therefore, we conclude that PVF coating was responsible for favorable Pt uptake above +0.4 V. Catalyst uptake was also observed in the orange region of Fig. 2B (<−0.4 V) with a maximum at −0.6 V (98 mg/g). The region of catalyst uptake coincided with the region in which platinum electrodeposition occurred (fig. S17, A and D), which is also in agreement with literature (*51*). XPS analysis of the working electrode confirmed the reduction of Speier’s catalyst to metallic Pt(0) (Fig. 2B), indicating that uptake at potentials more negative than −0.4 V can be fully attributed to irreversible platinum electrodeposition.

Platinum electrodeposition at −0.6 V was found to consume several orders of magnitude more energy (*E*_−0.6V_ = 5.179 kJ/g of PVF) than electrosorption at +0.5 V (*E*_+0.5V_ = 0.162 kJ/g of PVF; fig. S19E). The energy savings of the electrosorption method were largely due to the high reversible uptake of Pt via selective binding toward PVF-CNT versus the energy-intensive Faradaic process of electrodeposition, which often suffers high mass transfer overpotentials at low Pt concentrations (*51*, *52*). XPS analysis of the counter electrode at potentials higher than +0.6 V showed metallic platinum formation (fig. S11B), indicating that high anodic potentials on the working PVF-CNT electrode can lead to favorable electrodeposition conditions at the counter electrode. Therefore, +0.5 V was chosen as the optimal adsorption potential due to its superior catalyst uptake (162 mg/g) without alteration of the platinum oxidation state of Speier’s catalyst on either the working or counter electrode.

After catalyst adsorption at +0.5 V, Speier’s catalyst was released from the redox electrode into a Pt-free EtOH solution via an applied potential within the yellow region of Fig. 2B (−0.2 V < E < +0.3 V), where the applied potential was too high to electrodeposit Speier’s catalyst (<−0.4 V) and low enough to reduce ferrocene(III) to ferrocenium(II) (<+0.4 V). A maximum recovery efficiency (defined as the mass Pt desorbed/adsorbed) of 99.5% was observed at +0.1 V (Fig. 1C), demonstrating excellent catalyst recyclability. XPS analysis of remaining platinum on the working (Fig. 1C) and counter (fig. S15A) electrodes after desorption showed only Pt(II), indicating that no electroreduction of platinum catalyst occurred during release. XPS analysis of Fe on the working electrode (fig. S14) after catalyst desorption showed complete reduction of Fe(III) to Fe(II) for all potentials up to +0.3 V. However, at +0.3 V, 40% of iron remained oxidized, corresponding to a lower recovery efficiency of 70.9% and demonstrating that the mechanism of catalyst release correlates with reduction of Fe(III). Energy consumption for desorption expectedly increased with increasingly negative applied potential (fig. S20). Therefore, an optimal desorption potential of +0.1 V was chosen for all subsequent experiments due to its high Pt recovery efficiency (99.5%) and low energy consumption (0.53 kJ/g of PVF).

#### Palladium catalyst recycle

Similarly, a series of batch scale experiments with the PVF//CP system with 1 mM palladium chloride in methanol with 20 mM copper(II) chloride as a model system for Wacker oxidation were tested at various chronoamperometric potentials (from 0.0 to 1.0 V versus Ag/AgCl). Similar to the Pt electrosorption results, three distinct regions were observed in fig. S25A for Pd electrosorption: PVF-mediated electrosorption region, >0.4 V; Pd electrodeposition region, <0.0 V; and region of safe desorption, between 0.0 and 0.4 V. Within the PVF electrosorption region (>0.4 V), we observed high Pd uptake (116 mg/g maxima at 0.8 V), and Pd XPS spectra (fig. S16B) of electrodes showed that the Pd(II) oxidation state was unaltered. PVF-free adsorption at 0.6 V (fig. S25B) showed no appreciable palladium uptake, confirming the redox-mediated mechanism for palladium catalyst recovery at potentials above 0.4 V using PVF-CNT. At an applied potential below 0.2 V, Pd uptake was again observed (111 mg/g maxima at 0.0 V) where palladium electrodeposition was observed to occur (fig. S25D), with Pd(0) observed on the working electrode at <0.0 V. Following PVF electrosorption at 0.6 V, recovery of captured palladium catalyst was possible electrochemically via release through an applied potential of 0.1 V with a recovery efficiency of 92%, and no sign of palladium was observed with XPS analysis on the fully regenerated electrode (fig. S16C). As shown, redox potential plays a key role in avoiding destructive electrodeposition, and while PVF was selected as a model polymer in the current work due to its availability and intrinsic charge-transfer properties, future work in tuning redox potential in derivative polymers can further improve efficiency.

### Mechanistic investigation of catalyst binding via electronic structure calculations

To obtain atomistic insights into the experimentally observed separation trends, we carried out density functional theory (DFT) and domain-based local pair natural orbital coupled-cluster theory calculations to corroborate separation trends and characterize the binding mechanism of ferrocenium cation (Fc^+^) interacting with Speier’s catalyst ([PtCl_3_(CH_2_CHCH_3_)]^−^), Wacker oxidation catalyst ([PdCl_4_]^2−^), Suzuki coupling catalyst ([PdCl_2_(PPh_3_)_2_]^2−^), and relevant competing ions (Cl^−^ and ClO_4_^−^). The Pt and Pd complexes in Speier’s and Wacker catalysts interact with the cyclopentadiene ring of Fc^+^ through C─H···Cl close contacts (2.6- to 3.1-Å distance; figs. S2 and S3). For Suzuki coupling, Fc^+^ interacts with [PdCl_2_(PPh_3_)_2_]^2−^ and [PdCl(PPh_3_)_2_]^−^ through noncovalent bond formation with Pd(0) with a C─H···Pd distance around 2.6 Å (fig. S4). Both Suzuki coupling complexes also exhibit the stabilizing C─H···π interaction between cyclopentadiene and phenyl rings at 2.5- to 2.6-Å distance. The binding affinity trend (Fig. 2D and fig. S6) reveals preferential sorption of catalysts over spectator anions for all catalytic systems considered, in agreement with experiments. Speier’s catalyst in EtOH interacts more strongly with Fc^+^ than perchlorate, with the binding affinity trend [PtCl_3_(C_3_H_6_)]^−^ ≈ [PtCl_6_]^2−^ > [ClO_4_]^−^. Polarizable solvents with a higher dielectric constant [such as acetonitrile (MeCN)] solvates individual ions more strongly, destabilizing the ionic pair adducts and disfavoring sorption (fig. S6A). Similarly, Wacker catalyst is adsorbed preferentially over Cl^−^, and the binding affinity presents an order of [PdCl_3_(CH_2_CHC_6_H_5_)]^−^ > [PdCl_4_]^2−^ > Cl^−^. For Suzuki coupling catalysts, the binding affinity decreases as [PdCl(PPh_3_)_2_]^−^ > [PdCl_2_(PPh_3_)_2_]^2−^ > Cl^−^. Adsorption of bromide-substituted Suzuki coupling catalysts is more pronounced than their chloride counterparts, likely because of smaller Cl^−^ size and thus higher surface charge density that promotes ion solvation and destabilizes adducts. In sum, the results indicate the stronger binding of the catalyst active complexes to ferrocenium over competing inorganic salts from solution, in alignment with the experimental results presented.

A local energy decomposition analysis was performed to understand the contribution of different factors (electrostatic, exchange, dispersion, and charge transfer) to the interaction between Fc^+^ and catalysts/competitive ions (table S17). While electrostatics dominate the overall interaction energy, solvation of catalysts and Fc^+^ in polar solvents reduces electrostatic interactions upon adduct formation. According to table S18, charge transfer occurs predominantly from anions to ferrocenium cation, i.e., from highest occupied molecular orbital of catalysts/anions to lowest unoccupied molecular orbital of Fc^+^, playing a role in dictating selectivity. Table S18 shows that dispersion interactions are more prevalent in Pt/Pd complex systems, as compared to competitive anions due to the presence of more than two C─H···Cl close contacts in these systems. Dispersion energy maps are shown in fig. S5, indicating the atom pairs involved in dispersion interactions between Fc^+^ and anions.

### Redox electrosorption versatility toward solvent, electrolyte, and noble metal catalyst

#### Solvent selection

**Fig. 3.**
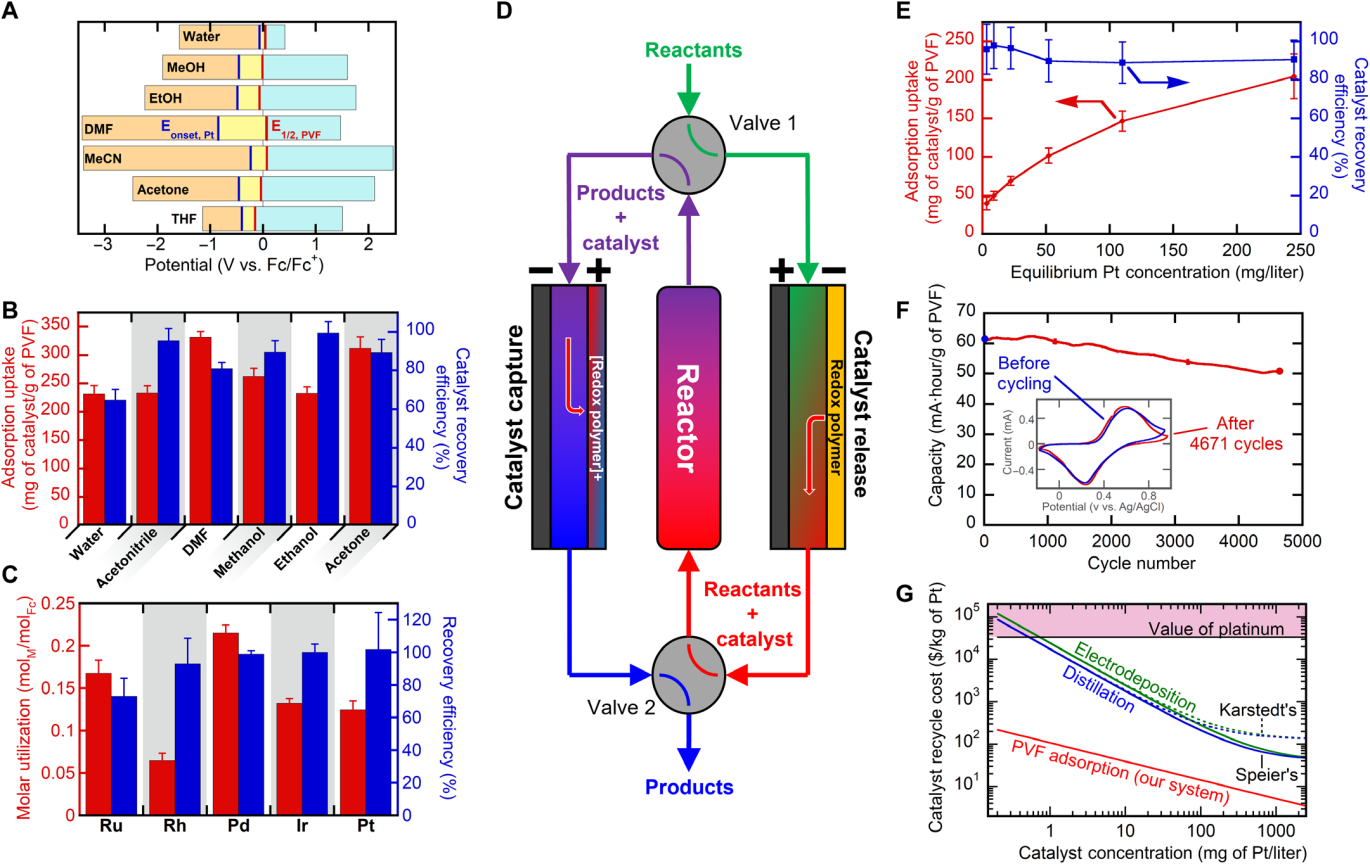
Feasibility study of PVF-mediated electrosorption under industrially relevant conditions. (**A**) Comparison of the standard potential of PVF the redox potential (red line), platinum electrodeposition potential (blue line), and the measured electrochemical window of stability for selected reaction solvents. (**B**) Catalyst recycle performance in the selected industrial solvents. (**C**) Electrochemical separation performance for PGM chloroanions. (**D**) Schematic diagram of a continuously operated catalyst recovery system. (**E**) Electrosorption isotherm of Speier’s catalyst in EtOH (red) and corresponding catalyst release efficiency (blue). (**F**) PVF-CNT cycling performance over 4671 charge/discharge cycles in EtOH. Inlaid figure depicts a CV of the electrode before and after cycling. (**G**) Estimated cost to recycle Speier’s (solid lines) and Karstedt’s (dashed lines) per the concentration of catalyst exiting the reactor for three different catalyst recovery methods: PVF adsorption (red), distillation (blue), and electrodeposition (green).

Recycle performance of the PVF//CP system was tested in relevant industrial solvents, covering range of dielectric values (*53*): dimethylformamide, MeCN, methanol, acetone, tetrahydrofuran (THF), water, and EtOH. Successful recycle of Speier’s catalyst was possible for all solvents with an average uptake of 266 ± 44 mg/g of PVF and recovery efficiency of 87 ± 12% (Fig. 3B). The solvent stability window, redox potential of PVF, and the reduction potential of Speier’s catalyst were determined for each system to evaluate the impact of solvent choice. Figure 3A shows a blue region where PVF can oxidize and a yellow region representing where PVF can safely reduce, without side reactions. The orange region indicates a region where Speier’s catalyst will electrodeposit. The lowest catalyst recovery efficiency of 68% was obtained in water, where the highest electrodeposition potential was observed. For each solvent, the solubility/stability of PVF was determined by measuring faradaic capacity over several cycles using CVs (fig. S22). Protic solvents such as EtOH and methanol showed virtually no loss of capacity. While some capacity loss was apparent in *N*,*N*-dimethylformamide (DMF), MeCM, acetone, and THF, cross-linking of the PVF-CNT enhanced electrode stability by 87% on average. Our study demonstrates the compatibility of the redox-electrode platform with a range of relatively polar solvent systems, due to the suitability of these solvents with the model reaction being explored and the stability of the polymer-functionalized electrode. In the future, expansion and evaluation of these methods for other highly nonpolar organic solvents can be explored, with more robust electrode functionalization methods being potentially required.

#### Electrolyte selection

Recovery of Speier’s catalyst was found remarkably insensitive to the identity of the electrolyte species, showing consistent catalyst uptake of 230 ± 5 mg/g and 99.3 ± 0.8% regeneration efficiency across a range of commonly employed electrolytes (fig. S21C). Many industrial catalytic systems use ionic catalyst promoters and cocatalysts. For the case of hydrosilylation, aluminum chloride (*54*) and lithium chloride (*55*) are two such catalyst-stabilizing promoters. PVF adsorption remained selective to Speier’s catalyst while in the presence of 20-fold excess of competing LiCl and AlCl_3_ ionic hydrosilylation promoters, without hindering catalyst uptake (LiCl, 234 mg/g; AlCl_3_, 213 mg/g) or recovery efficiency (LiCl, 99.9%; AlCl_3_, 99.6%).

#### Generality to PGM recovery

Last, we evaluated PVF-CNT recovery performance with a range of PGM chloroanions of value: RuCl_5_NO^2−^, RhCl_6_^3−^, PdCl_4_^2−^, IrCl_6_^2−^, and PtCl_6_^2−^, many of which are precatalysts or catalysts for homogeneous reactions themselves. Electrosorption of 1 mM for each PGM salt (0.5 V versus Ag/AgCl, 30 min) resulted in a notably uptake of all target PGMs in Fig. 3C, with palladium achieving the highest (0.21 mol of metal/mol of ferrocene) and rhodium with the lowest (0.07 mol of metal/mol of ferrocene). Electrochemical release was also successful for all PGM anions, achieving a recovery efficiency of 73% with ruthenium and a 100% recovery efficiency with iridium and platinum. These results demonstrate the generality of the electrochemical recycling approach beyond Pt or Pd, being practical also for other PGM catalysts such as rhodium and iridium, which are key metal centers in homogeneous catalysis (*56*–*59*).

### Continuous flow process design and technoeconomic feasibility

#### Scale up and flow cell design

**Fig. 4.**
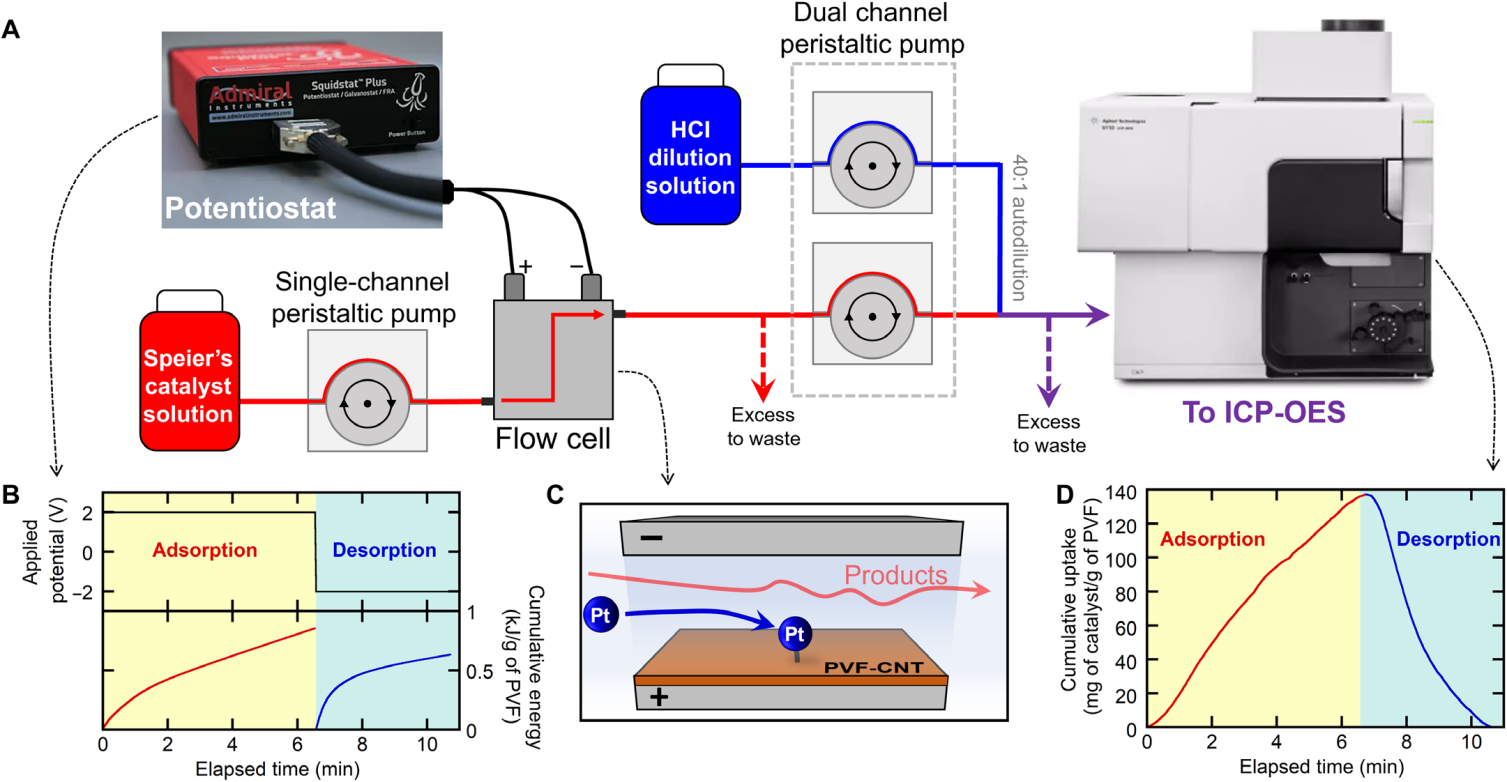
Schematic diagram of flow-cell configuration for homogeneous catalyst recovery. (**A**) Schematic of electrochemical PVF-CNT flow-by cell with inline ICP-OES for monitoring Speier’s catalyst capture and release. (**B**) Electrochemical voltage input (top) and current response (bottom) during electrosorption. (**C**) Diagram of the flow-by cell with PVF-CNT coating on anode during catalyst separation. (**D**) Real-time monitoring of Speier’s catalyst adsorption and release for flow cell.

A two-electrode continuous flow-by cell was fabricated with a 16-fold increase in electrode area. Catalyst adsorption was carried out with an applied total cell potential of +2.0 V and desorption at −2.0 V, with operation based on optimal conditions from previous three-electrode experiments. A 1 mM Speier’s catalyst in EtOH solution flowed through the flow-by cell at 1.0 ml/min. Inline ICP measurements of atomic platinum concentration was recorded downstream of the flow-by cell with 1-s resolution (Fig. 4A). The flow cell achieved a maximum cumulative uptake of 151 mg/g within 6 min and a release of 95% of adsorbed Speier’s catalyst within 4 min (Fig. 4, A and D), in agreement with batch performance. PVF energy (0.7 kJ/g) was consumed during electrosorption and 0.55 kJ/g of PVF during electrorelease (Fig. 4B). The flow cell results showed that scale-up of the PVF-CNT catalyst recycle system was feasible with no loss of performance and could even successfully concentrate the catalyst from 162 to 255 ppm upon release (fig. S27). Building from flow cell results, a preliminary technoeconomic analysis (TEA) demonstrated the feasibility of the PVF system for economically favorable Pt catalyst recovery with 99.8% less energy consumption and 85% lower overall recovery costs compared to other competing recovery methods (see Fig. 3G and details in section S4). Our TEA focuses on hydrosilylation costs using both Speier and Karstedt’s catalysts and on the feasibility of the catalyst recovery step—with a major advantage being the preservation of the catalytic activity of these complex catalysts versus destructive thermal methods that require resynthesis of the organometallic system. A more extensive TEA involving each step of product isolation and downstream processing for the broader scope of reactions will be considered.

Here, we develop redox-active electrosorbent platforms for the capture and release of key classes of homogeneous catalysts, namely, for hydrosilylation, silane etherification, Wacker oxidation, and Suzuki cross-coupling. Through functionalization of electrode surfaces with a redox metallopolymer and judicious tuning of sorption and release potentials, irreversible electrodeposition of the PGMs is avoided, and full preservation of catalytic activity is achieved for a broad scope of substrates, solvents, and competing species. The role of charged catalytic species was investigated by spectroscopic methods and electronic structure calculations during the catalyst recycling process. Translation of our redox system to continuous flow processes showed preservation of uptake capacity and regeneration efficiency compared to the batch results. Going forward, we envision the concept of electrochemical recycling to be generalized to broader classes of homogeneous catalysts. With the gradual shift toward electrification and the new paradigm for reduce, reuse, and recycle, electrochemical catalyst recovery can play a key role in ushering sustainable and resource-friendly chemical manufacturing and process synthesis.

## MATERIALS AND METHODS

### PVF-CNT electrode synthesis

PVF-CNT ink solution was prepared using previously reported methods (*60*). Eighty milligrams of poly(vinyl)ferrocene and 40 mg of vacuum-dried multiwalled CNTs (MWCNTs) were added to 10 ml of chloroform to make solution “A.” A separate solution “B” was simultaneous prepared containing 40 mg of MWCNTs in 10 ml of chloroform (*38*). Both solutions A and B were sealed and ultrasonicated at a temperature less than 15°C for 30 min. After sonication, solutions A and B are combined and sonicated a second time at a temperature less than 15°C for 30 min. PVF-CNT ink solution containing PVF (4 g/liter) and MWCNT (4 g/liter) was then applied to a current collector to produce a PVF-CNT electrode. A 0.762 mm thick 316 stainless steel sheet (McMaster Carr), 0.001-inch-thick graphene (McMaster Carr), and Toray 030 carbon paper were used as the current collector materials and cut into 1-cm by 3-cm strips; stainless steel sheets were lightly sanded with 120-grit sandpaper for better coating adherence. Fifty microliters of PVF-CNT ink was drop-coated onto the current collector and spread to cover a 1-cm by 1-cm area using a pipette tip. The PVF-CNT–coated electrode was left to dry from benchtop at room temperature and yielded a 0.4 mg of PVF-CNT coating consisting of 0.2 mg of PVF.

For the solvent versatility test in fig. S22, 1,3-benzenedisulfonyl azide was synthesized on the basis of a method in a literature (*61*). It was added to the PVF-CNT ink solution as a cross-linker [20 weight percent (wt %) of PVF] to prevent the dissolution of PVF in organic solvents (i.e., DMF, MeCN, acetone, and THF). After following the same coating procedure above, the coated electrode was put into an oven and cross-linked at 160°C for 1.5 hour.

### Analytical batch cell experimentation

Adsorption/desorption batch cell experiments were conducted with three-dimensional (3D) printed electrochemical batch cells. The 3D printed batch cells were designed in-house to maximize the electrode area to solution volume (~1 cm^2^/ml), maintained consistent geometry between working and counter electrode (parallel spacing of 1 cm^2^), and inhibited organic solvent evaporation. All printed parts were constructed with polypropylene on a Prusa Research i3 MK3S direct-drive fused deposition modeling 3D printer with a layer thickness of 0.1 mm and 100% infill. All adsorption/desorption experiments were conducted in a printed cell containing a 1-cm by 3-cm PVF-CNT working electrode, 1-cm by 3-cm plain carbon paper counter electrode, a reference electrode (either aqueous Ag/AgCl or nonaqueous AgCl), and a small stir bar. The cells were filled with 1 ml of analytical solution [1 mM Speier’s catalyst and 20 mM TBAP in EtOH, unless otherwise specified] that contacted the lower 1 cm by 1 cm of each electrode and then sealed with a 3D printed cap. Electrochemical experiments were conducted with BioLogic SP-200 single-channel potentiostat. Unless otherwise specified, +0.5 V versus Ag/AgCl was applied onto the PVF-CNT electrode for 30 min for electrosorption. Regeneration of PVF-CNT redox electrode was accomplished by applying +0.1 V versus Ag/AgCl onto the PVF-CNT electrode for 30 min in clean 20 mM TBAP solution (unless another supporting electrolyte is specified).

### Electrode cyclability tests

A cycle consisted of rapid chronopotentiometric charging at +4 A/g (polymer) (+10 A/m^2^) until the two-electrode potential reached 2.15 V, followed by rapid chronopotentiometric discharging at −4 A/g (polymer) until a potential of −2.45 V was reached, and after 4671 charge/discharge cycles, the cell energy capacity only decreased by 15% to 50.8 mA·h/g of PVF.

### Analysis of total Pt and Pd in solution

The concentration of total dissolved Pt within solution was quantified using ICP-OES (Agilent 5110). A HCl dilution solution (5 wt %) was prepared from 38% HCl (Thermo Fisher Scientific) and used to dilute calibration standards and aqueous samples. Four standard solutions were prepared for both Pt and Pd by diluting the ICP calibration standard [platinum standard for ICP TraceCERT, Pt (1000 mg/liter) in hydrochloric acid (Sigma-Aldrich), and palladium standard for ICP TraceCERT, Pd in hydrochloric acid (1000 mg/liter)] with 5 wt % of HCl dilution solution. After calibration, the linear fit was visualized, ensuring *R*^2^ of >0.999 for every measurement. Silane-containing samples were prepared for ICP-OES by drying 100 μl of sample in a vacuum oven at 100°C until complete evaporation, and the solid platinum was then digested in 1 ml of aqua regia (3 ml of HCl to 1 ml of HNO_3_) and diluted with 4 ml of deionized (DI) water after 1 hour of digestion. Each sample was measured with at least 10 replicates by spectrometer to yield a reliable averaged reading. Samples (100 μl) containing PdCl_2_(PPh_3_)_2_ for cross-coupling experiments were digested in 1 ml of aqua regia, vortex-mixed for 30 s, and diluted with 9 ml of DI water.

### Flow-by cell and inline ICP

The flow-by cell used in this work consisted of two carbon paper electrodes (active area of 4 cm by 4 cm) sealed between acrylic backing plates with a 1/32-inch Viton rubber gasket. A Teflon mesh was placed between electrodes to increase turbulence and reduce the cell’s internal volume to 1 ml. Titanium current collectors mechanically and electrically supported the PVF-CNT–coated carbon paper working electrode and plain carbon paper counter electrode. No reference electrode was used with the flow-by cell. The 4-cm by 4-cm PVF-CNT–coated carbon paper electrode was produced using the same method as batch cell electrodes, where 0.8 ml of PVF-CNT ink solution was drop-coated via pipette to fully cover a 4-cm by 4-cm carbon paper sheet.

Fresh analytical solution was continuously pumped to the flow-by cell with a Longer peristaltic pump located upstream of the cell. Downstream of the flow-by cell, the stream is autodiluted with a two-channel peristaltic pump, and the diluted stream is sent directly to the ICP-OES for immediate analysis. Autodilution is carried out by pumping the flow cell stream with a 0.5-mm-diameter peristaltic tube in the first channel, pumping 5% HCl in DI water with a 3.17-mm-diameter peristaltic tube with the second channel of the same pump, and combining the two streams. Dilution ratio (initial Pt concentration/diluted Pt concentration) was controlled by the two different tube diameters, and 40:1 dilution was maintained.

### Electrochemical stability test in various solvents

The ferrocene (II/III) couple was used for a reference potential. To determine the half-potential of the ferrocene in each organic solvent, CV of six cycles at 20 mV/s (−0.5 to 1.0 V versus Ag/Ag^+^) was used in 3 ml of 1 mM ferrocene and 20 mM TBAPF_6_ (1-cm by 1-cm carbon paper working, Pt wire counter, and nonaqueous Ag/Ag^+^ reference). The half-potential was calculated by averaging the oxidation and reduction peak potentials at the second cycle. For water, the half-potential value was adapted from literature (*62*). The half-potential of PVF on a PVF-CNT electrode was determined with the same CV setting above (−0.7 to 1.0 V versus Ag/Ag^+^) in 3 ml of 20 mM TBAPF_6_ (1-cm by 1-cm PVF-CNT working, carbon paper counter, and nonaqueous Ag/Ag^+^ reference). The half-potential was calculated by averaging the oxidation and reduction peak potentials at the fifth stable cycle. For DMF, MeCN, acetone, and THF, cross-linked PVF-CNT electrodes were used. The cumulative charge of each cycle in fig. S22 was calculated by calculating the difference between the maximum and minimum charge and then normalized by the cumulative charge of the second cycle as the very first cycles were disregarded. To obtain the potential stability window of organic solvents, current versus the counter potential (carbon paper) from the above PVF-CNT cyclic voltammograms was plotted for DMF, MeCN, acetone, and THF, while another CV (−2.5 to 2.5 V versus Ag/Ag^+^) was taken for EtOH and methanol with 20 mM TBAPF_6_ (1-cm by 1-cm carbon paper working, Pt wire counter, and nonaqueous Ag/Ag^+^ reference). The oxidation and reduction onset potential of each solvent was calculated by obtaining an intersection between the current baseline and the tangent line of oxidative and reductive current, respectively. The onset potential of Pt electrodeposition with Speier’s catalyst was determined by CV at 50 mV/s (−1.0 to 0.5 V versus Ag/Ag^+^) in 1 mM Speier’s catalyst 0.1 M TBAPF_6_ (1-cm by 1-cm carbon paper working, Pt wire counter, and nonaqueous Ag/Ag^+^ reference). The Pt electrodeposition onset potentials were calculated in the same way used for the onset potentials of solvent oxidation and reduction.

### General catalyst recycling procedure

A control reaction with as-received noble metal catalyst is reacted to completion to act as the adsorbing solution. The used catalyst is electrochemically adsorbed with the PVF-CNT cell via an oxidizing potential for 30 min. The concentration of catalyst in solution before and after adsorption was determined with ICP-OES. The catalyst-laden PVF-CNT electrode was transferred to a new solution of fresh reactants where catalyst was released via electrochemical reduction with applied potential for 30 min. The solution with recycled catalyst was given time to fully react. Specifics of the reaction conditions, depending on the catalyst system, can be found in detail in the Supplementary Materials.

## Supplementary Materials

### This PDF file includes:

Supplementary Materials and Methods Supplementary Text Figs. S1 to S69 Tables S1 to S20 References

## References

[1] C. A. Tolman, J. P. Jesson, Homogeneous catalysis. Science 181, 501–505 (1973). 17777791 10.1126/science.181.4099.501

[2] D. J. Cole-Hasmilton, Homogeneous catalysis - New approaches to catalyst separation, recovery, and recycling. Science 299, 1702–1706 (2003). 12637737 10.1126/science.1081881

[3] B. Cornils, W. A. Herrmann, *Applied Homogeneous Catalysis with Organometallic Compounds: A Comprehensive Handbook in Two Volumes* (Wiley-VCH, 1996).

[4] J. A. Hueffel, T. Sperger, I. Funes-Ardoiz, J. S. Ward, K. Rissanen, F. Schoenebeck, Accelerated dinuclear palladium catalyst identification through unsupervised machine learning. Science 374, 1134–1140 (2021). 34822285 10.1126/science.abj0999

[5] S. Park, M. Brookhart, Hydrosilylation of carbonyl-containing substrates catalyzed by an electrophilic η1-silane iridium(III) complex. Organometallics 29, 6057–6064 (2010). 21572562 10.1021/om100818y PMC3092162

[6] M. Benaglia, in *Recoverable and Recyclable Catalysts* (Wiley, ed. 1, 2009), pp. xviii, 471 p.

[7] N. D. Knofel, H. Rothfuss, J. Willenbacher, C. Barner-Kowollik, P. W. Roesky, Platinum(II)-crosslinked single-chain nanoparticles: An approach towards recyclable homogeneous catalysts. Angew. Chem. Int. Ed. 56, 4950–4954 (2017). 10.1002/anie.201700718 28371045

[8] B. Cornils, W. A. Herrmann, Concepts in homogeneous catalysis: The industrial view. J. Catal. 216, 23–31 (2003).

[9] D. Troegel, J. Stohrer, Recent advances and actual challenges in late transition metal catalyzed hydrosilylation of olefins from an industrial point of view. Coordin. Chem. Rev. 255, 1440–1459 (2011).

[10] R. J. Hofmann, M. Vlatkovic, F. Wiesbrock, Fifty years of hydrosilylation in polymer science: A review of current trends of low-cost transition-metal and metal-free catalysts, non-thermally triggered hydrosilylation reactions, and industrial applications. Polymers 9, 534 (2017). 30965835 10.3390/polym9100534 PMC6418815

[11] B. Marciniec, in *Hydrosilylation of Alkenes and Their Derivatives* (Hydrosilylation: A Comprehensive Review on Recent Advances, Springer, 2009), vol. 1, pp. 3–51.

[12] L. D. de Almeida, H. Wang, K. Junge, X. Cui, M. Beller, Recent advances in catalytic hydrosilylations: Developments beyond traditional platinum catalysts. Angew. Chem. Int. Ed. Engl. 60, 550–565 (2021). 32668079 10.1002/anie.202008729 PMC7839722

[13] C. K. Blasius, H. Wadepohl, L. H. Gade, NNN-cobalt(II) pincer complexes: Paramagnetic NMR spectroscopy in solution and application as hydrosilylation catalysts. Eur. J. Inorg. Chem. 2020, 2335–2342 (2020).

[14] W. J. Teo, C. Wang, Y. W. Tan, S. Z. Ge, Cobalt-catalyzed *Z*-selective hydrosilylation of terminal alkynes. Angew. Chem. Int. Ed. 56, 4328–4332 (2017). 10.1002/anie.201700868 28267265

[15] Y. F. Wei, S. X. Liu, H. Mueller-Bunz, M. Albrecht, Synthesis of triazolylidene nickel complexes and their catalytic application in selective aldehyde hydrosilylation. ACS Catal. 6, 8192–8200 (2016).

[16] Y. Nakajima, S. Shimada, Hydrosilylation reaction of olefins: Recent advances and perspectives. RSC Adv. 5, 20603–20616 (2015).

[17] H. B. Li, C. C. C. J. Seechurn, T. J. Colacot, Development of preformed Pd catalysts for cross-coupling reactions, beyond the 2010 nobel prize. ACS Catal. 2, 1147–1164 (2012).

[18] K. C. Nicolaou, E. J. Sorensen, in *Classics in Total Synthesis: Targets, Strategies, Methods* (Wiley-VCH, 1996), pp. xxiii, 798 p.

[19] J. Magano, J. R. Dunetz, Recent large-scale applications of transition metal-catalyzed couplings for the synthesis of pharmaceuticals. Rsc Catal. Ser., 697–778 (2015). 10.1021/cr100346g 21391570

[20] D. J. Cole-Hamilton, R. P. Tooze, in *Catalyst Separation, Recovery and Recycling: Chemistry and Process Design* (Catalysis by Metal Complexes, Springer, 2006), pp. ix, 248 p.

[21] J. Hagen, in *Industrial Catalysis: A Practical Approach* (John Wiley & Sons, 2006), pp. 1–507.

[22] A. E. Schweizer, G. T. Kerr, Thermal-decomposition of hexachloroplatinic acid. Inorg. Chem. 17, 2326–2327 (1978).

[23] P. W. N. M. v. Leeuwen, J. C. Chadwick, *Homogeneous Catalysts. Activity—Stability—Deactivation* (Wiley-VCH, 2011), pp. 1 online resource (xiii, 404 pages).

[24] G. Duca, in *Homogeneous Catalysis with Metal Complexes: Fundamentals and Applications* (Springer Series in Chemical Physics, Springer, 2012), pp. 1 online resource.

[25] J. L. Barton, Electrification of the chemical industry. Science 368, 1181–1182 (2020). 32527815 10.1126/science.abb8061

[26] D. S. Sholl, R. P. Lively, Seven chemical separations to change the world. Nature 532, 435–437 (2016). 27121824 10.1038/532435a

[27] X. Su, Electrochemical interfaces for chemical and biomolecular separations. Curr. Opin. Colloid. Interface Sci. 46, 77–93 (2020).

[28] M. E. Suss, S. Porada, X. Sun, P. M. Biesheuvel, J. Yoon, V. Presser, Water desalination via capacitive deionization: What is it and what can we expect from it? Energ. Environ. Sci. 8, 2296–2319 (2015).

[29] R. Candeago, K. Kim, H. Vapnik, S. Cotty, M. Aubin, S. Berensmeier, A. Kushima, X. Su, Semiconducting polymer interfaces for electrochemically assisted mercury remediation. ACS Appl. Mater. Interfaces 12, 49713–49722 (2020). 33079513 10.1021/acsami.0c15570

[30] X. Su, K.-J. Tan, J. Elbert, C. Ruttiger, M. Gallei, T. F. Jamison, T. A. Hatton, Asymmetric Faradaic systems for selective electrochemical separations. Energ. Environ. Sci. 10, 1272–1283 (2017).

[31] X. Su, L. Bromberg, K. J. Tan, T. F. Jamison, L. P. Padhye, T. A. Hatton, Electrochemically mediated reduction of nitrosamines by hemin-functionalized redox electrodes. Environ. Sci. Tech. Lett. 4, 161–167 (2017).

[32] F. Y. Cheng, J. Liang, Z. L. Tao, J. Chen, Functional materials for rechargeable batteries. Adv. Mater. 23, 1695–1715 (2011). 21394791 10.1002/adma.201003587

[33] K. Kim, S. J. Lee, D. Y. Kim, C. Y. Yoo, J. W. Choi, J. N. Kim, Y. Woo, H. C. Yoon, J. I. Han, Electrochemical synthesis of ammonia from water and nitrogen: A lithium-mediated approach using lithium-ion conducting glass ceramics. ChemSusChem 11, 120–124 (2018). 29105332 10.1002/cssc.201701975

[34] A. K. Dutta, F. Neese, R. Izsak, Speeding up equation of motion coupled cluster theory with the chain of spheres approximation. J. Chem. Phys. 144, 034102 (2016). 26801015 10.1063/1.4939844

[35] X. Su, A. Kushima, C. Halliday, J. Zhou, J. Li, T. A. Hatton, Electrochemically-mediated selective capture of heavy metal chromium and arsenic oxyanions from water. Nat. Commun. 9, 4701 (2018). 30409968 10.1038/s41467-018-07159-0 PMC6224381

[36] K. Kim, S. Cotty, J. Elbert, R. L. Chen, C. H. Hou, X. Su, Asymmetric redox-polymer interfaces for electrochemical reactive separations: Synergistic capture and conversion of arsenic. Adv. Mater. 32, 1906877 (2020). 10.1002/adma.201906877 31793695

[37] K. Kim, P. B. Medina, J. Elbert, E. Kayiwa, R. D. Cusick, Y. J. Men, X. Su, Molecular tuning of redox-copolymers for selective electrochemical remediation. Adv. Funct. Mater. 30, 2004635 (2020).

[38] X. Su, J. Hubner, M. J. Kauke, L. Dalbosco, J. Thomas, C. C. Gonzalez, E. Zhu, M. Franzreb, T. F. Jamison, T. A. Hatton, Redox interfaces for electrochemically controlled protein-surface interactions: Bioseparations and heterogeneous enzyme catalysis. Chem. Mater. 29, 5702–5712 (2017).

[39] J. L. Speier, J. A. Webster, G. H. Barnes, The addition of silicon hydrides to olefinic double bonds. Part II. The use of group-VIII metal catalysts. J. Am. Chem. Soc. 79, 974–979 (1957).

[40] J. E. Baeckvall, B. Akermark, S. O. Ljunggren, Stereochemistry and mechanism for the palladium(II)-catalyzed oxidation of ethene in water (the Wacker process). J. Am. Chem. Soc. 101, 2411–2416 (1979).

[41] N. Miyaura, A. Suzuki, Palladium-catalyzed cross-coupling reactions of organoboron compounds. Chem. Rev. 95, 2457–2483 (1995).

[42] R. A. Benkeser, J. Kang, The composition of speier’s catalyst. J. Organomet. Chem. 185, C9–C12 (1980).

[43] J. Stein, L. N. Lewis, Y. Gao, R. A. Scott, In situ determination of the active catalyst in hydrosilylation reactions using highly reactive Pt(0) catalyst precursors. J. Am. Chem. Soc. 121, 3693–3703 (1999).

[44] P. W. N. M. Leeuwen, Oxidation with dioxygen, in *Homogeneous Catalysis: Understanding the Art* (Springer, 2004), pp. 319–336.

[45] R. A. Fernandes, A. K. Jha, P. Kumar, Recent advances in Wacker oxidation: From conventional to modern variants and applications. Cat. Sci. Technol. 10, 7448–7470 (2020).

[46] C. Amatore, A. Jutand, Anionic Pd(0) and Pd(II) intermediates in palladium-catalyzed Heck and cross-coupling reactions. Acc. Chem. Res. 33, 314–321 (2000). 10813876 10.1021/ar980063a

[47] A. A. Thomas, S. E. Denmark, Pre-transmetalation intermediates in the Suzuki-Miyaura reaction revealed: The missing link. Science 352, 329–332 (2016). 27081068 10.1126/science.aad6981

[48] F. Schroeter, J. Soellner, T. Strassner, Cross-coupling catalysis by an anionic palladium complex. ACS Catal. 7, 3004–3009 (2017).

[49] M. Peuckert, H. P. Bonzel, Characterization of oxidized platinum surfaces by x-ray photoelectron-spectroscopy. Surf. Sci. 145, 239–259 (1984).

[50] A. Romanchenko, M. Likhatski, Y. Mikhlin, X-ray photoelectron spectroscopy (XPS) study of the products formed on sulfide minerals upon the interaction with aqueous platinum (IV) chloride complexes. Minerals 8, 578 (2018).

[51] H. M. Yasin, G. Denuault, D. Pletcher, Studies of the electrodeposition of platinum metal from a hexachloroplatinic acid bath. J. Electroanal. Chem. 633, 327–332 (2009).

[52] M. E. Baumgartner, D. R. Gabe, Palladium-iron alloy electrodeposition. Part I Single metal systems. Trans. IMF 78, 11–16 (2000).

[53] C. Capello, U. Fischer, K. Hungerbühler, What is a green solvent? A comprehensive framework for the environmental assessment of solvents. Green Chem. 9, 927–934 (2007).

[54] M. P. Sibi, Hydrogen hexachloroplatinate(IV), in *Encyclopedia of Reagents for Organic Synthesis* (2001), pp. 1–8.

[55] M. G. Voronkov, V. B. Pukhnarevich, N. I. Ushakova, I. I. Tsykhanskaya, A. I. Albanov, V. Y. Vitkovskii, Dehydrocondensation of trialkylsilanes with acetylenes and monoorganylacetylenes. Zh Obshch Khim 55, 94–100 (1985).

[56] R. L. Reyes, M. Sato, T. Iwai, K. Suzuki, S. Maeda, M. Sawamura, Asymmetric remote C–H borylation of aliphatic amides and esters with a modular iridium catalyst. Science 369, 970–974 (2020). 32820123 10.1126/science.abc8320

[57] S. Y. Hong, Y. Park, Y. Hwang, Y. B. Kim, M. H. Baik, S. Chang, Selective formation of γ-lactams via C-H amidation enabled by tailored iridium catalysts. Science 359, 1016–1021 (2018). 29496875 10.1126/science.aap7503

[58] B. A. Vaughan, M. S. Webster-Gardiner, T. R. Cundari, T. B. Gunnoe, A rhodium catalyst for single-step styrene production from benzene and ethylene. Science 348, 421–424 (2015). 25908817 10.1126/science.aaa2260

[59] J. Y. Cho, M. K. Tse, D. Holmes, R. E. Maleczka, M. R. Smith III, Remarkably selective iridium catalysts for the elaboration of aromatic C–H bonds. Science 295, 305–308 (2002). 11719693 10.1126/science.1067074

[60] Z. G. Yin, Q. D. Zheng, S. C. Chen, D. D. Cai, L. Y. Zhou, J. Zhang, Bandgap tunable Zn_1-*x*_Mg*_x_*O thin films as highly transparent cathode buffer layers for high-performance inverted polymer solar cells. Adv. Energy Mater. 4, 1301404 (2014).

[61] D. A. Baker, G. C. East, S. K. Mukhopadhyay, Synthesis and characterization of some disulfonyl azides as potential crosslinking agents for textile fibers. J. Appl. Polym. Sci. 79, 1092–1100 (2001).

[62] K. Lehmann, O. Yurchenko, G. Urban, Carbon nanowalls for oxygen reduction reaction in bio fuel cells. J. Phys. Conf. Ser. 557, 012008 (2014).

[63] J.-D. Lin, Q.-Y. Bi, L. Tao, T. Jiang, Y.-M. Liu, H.-Y. He, Y. Cao, Y.-D. Wang, Wettability-driven palladium catalysis for enhanced dehydrogenative coupling of organosilanes. ACS Catal. 7, 1720–1727 (2017).

[64] C. Xu, B. Huang, T. Yan, M. Cai, A recyclable and reusable K2PtCl4/Xphos-SO3Na/PEG-400/H2O system for highly regio- and stereoselective hydrosilylation of terminal alkynes. Green Chem. 20, 391–397 (2018).

[65] G. Kumar, J. R. Blackbur, R. G. Aldridge, W. E. Moddeman, M. M. Jones, Photoelectron spectroscopy of coordination compounds. II. Palladium complexes. Inorg Chem 11, 296–300 (1972).

[66] K. Plevova, B. Mudrakova, R. Sebesta, A practical three-step synthesis of vinylferrocene. Synthesis Stuttgart 50, 760–763 (2018).

[67] M. D. Hanwell, D. E. Curtis, D. C. Lonie, T. Vandermeersch, E. Zurek, G. R. Hutchison, Avogadro: An advanced semantic chemical editor, visualization, and analysis platform. J. Chem. 4, 17 (2012). 10.1186/1758-2946-4-17 PMC3542060 22889332

[68] A. Najibi, L. Goerigk, The nonlocal kernel in van der Waals density functionals as an additive correction: An extensive analysis with special emphasis on the B97M-V and ωB97M-V approaches. J. Chem. Theory Comput. 14, 5725–5738 (2018). 30299953 10.1021/acs.jctc.8b00842

[69] F. Weigend, R. Ahlrichs, Balanced basis sets of split valence, triple zeta valence and quadruple zeta valence quality for H to Rn: Design and assessment of accuracy. Phys. Chem. Chem. Phys. 7, 3297–3305 (2005). 16240044 10.1039/b508541a

[70] M. Garcia-Rates, F. Neese, Effect of the solute cavity on the solvation energy and its derivatives within the framework of the gaussian charge scheme. J. Comput. Chem. 41, 922–939 (2020). 31889331 10.1002/jcc.26139

[71] F. Neese, F. Wennmohs, U. Becker, C. Riplinger, The ORCA quantum chemistry program package. J. Chem. Phys. 152, 224108 (2020). 32534543 10.1063/5.0004608

[72] P. S. Albright, L. J. Gosting, Dielectric constants of the methanol-water system from 5 to 55°^1^. J. Am. Chem. Soc. 68, 1061–1063 (1946). 20985620 10.1021/ja01210a043

[73] J. Wyman, The dielectric constant of mixtures of ethyl alcohol and water from −5 to 40°. J. Am. Chem. Soc. 53, 3292–3301 (1931).

[74] M. J. Gilkey, A. V. Mironenko, D. G. Vlachos, B. J. Xu, Adipic acid production via metal-free selective hydrogenolysis of biomass-derived tetrahydrofuran-2,5-dicarboxylic acid. ACS Catal. 7, 6619–6634 (2017).

[75] S. Spicher, S. Grimme, Single-point Hessian calculations for improved vibrational frequencies and rigid-rotor-harmonic-oscillator thermodynamics. J. Chem. Theory Comput. 17, 1701–1714 (2021). 33554604 10.1021/acs.jctc.0c01306

[76] S. Grimme, Supramolecular binding thermodynamics by dispersion-corrected density functional theory. Chem. Eur. J. 18, 9955–9964 (2012). 22782805 10.1002/chem.201200497

[77] R. F. Ribeiro, A. V. Marenich, C. J. Cramer, D. G. Truhlar, Use of solution-phase vibrational frequencies in continuum models for the free energy of solvation. J. Phys. Chem. B 115, 14556–14562 (2011). 21875126 10.1021/jp205508z

[78] A. Altun, F. Neese, G. Bistoni, Effect of electron correlation on intermolecular interactions: A pair natural orbitals coupled cluster based local energy decomposition study. J. Chem. Theory Comput. 15, 215–228 (2019). 30495957 10.1021/acs.jctc.8b00915

[79] Y. Guo, C. Riplinger, U. Becker, D. G. Liakos, Y. Minenkov, L. Cavallo, F. Neese, Communication: An improved linear scaling perturbative triples correction for the domain based local pair-natural orbital based singles and doubles coupled cluster method [DLPNO-CCSD(T)]. J. Chem. Phys. 148, 011101 (2018). 29306283 10.1063/1.5011798

[80] G. L. Stoychev, A. A. Auer, F. Neese, Automatic generation of auxiliary basis sets. J. Chem. Theory Comput. 13, 554–562 (2017). 28005364 10.1021/acs.jctc.6b01041

