# Supplementary material for: Electrochemical recycling of homogeneous catalysts
Source: Sci Adv. 2022 Oct 19;8(42):eade3094. doi: 10.1126/sciadv.ade3094 (PMC9581474; doi:10.1126/sciadv.ade3094)
Supplement: Supplementary file 1 — Supplementary Materials and Methods Supplementary Text Figs. S1 to S69 Tables S1 to S20 References [file sciadv.ade3094_sm.pdf]

Science Advances
AAAS

Supplementary Materials for

Electrochemical recycling of homogeneous catalysts

Stephen Cotty *et al.*

Corresponding author: Xiao Su, x2su@illinois.edu

Sci. Adv. **8**, eade3094 (2022)
DOI: 10.1126/sciadv.ade3094

This PDF file includes:

Supplementary Materials and Methods
Supplementary Text
Figs. S1 to S69
Tables S1 to S20
References

1. Instruments and Materials

Karstedt's catalyst solution was obtained from Gelest Inc., and poly(vinylferrocene) was obtained from Polysciences, Inc. All other chemicals were obtained from Sigma Aldrich, VWR, Fisher Scientific Acros, Alfa Aesar or TCI, and used as received, unless otherwise stated. The nuclear magnetic resonance (NMR) spectra were obtained using a UNITY 500 NB (500 MHz; 5mm Varian 1H PFG Z probe) (Varian, USA). MestReNova14.1.0 was used to analyze all spectra. Electrospray-ionization mass spectrometry (ESI-MS) was performed with a Waters Q-TOF Ultima ESI or a Waters GCT Premier orthogonal acceleration time-of-flight (oa-TOF) mass spectrometer. The surface morphologies and elemental mapping images of the electrodes were obtained using a scanning electron microscope (SEM; Hitachi S-4700) operated at an accelerating voltage of 10 kV, equipped with energy dispersive X-ray spectroscopy (EDS; iXRF) with the accelerating voltage of 15 kV. The chemical states of iron and platinum on the electrodes were characterized using X-ray photoelectron spectroscopy (XPS; Kratos Axis ULTRA) with monochromatic Al Kα X-ray source (210 W). The XPS results were analyzed using CASA XPS software (UIUC license). The spectra were fitted into their components following subtraction of a Shirley background from the region of interest. Parameters for curve-fitting of Pt 4f, Pd 3d, and Fe 2p were determined from reported literature(49, 50, 63). The ToF-SIMS spectra were obtained using a PHI TRIFT III (Physical Electronics, USA) equipped with a liquid metal ion gun that bombards gold ions. For

the detection of negative Pt complexes, the second ion beam of Cs^{+} was used at 150-250 nA with an acceleration voltage of 2kV. Flash chromatography was performed using a Büchi Pure C-810 chromatography system with Büchi Pureflex Ecoflex silica cartridges as stationary phase.

2. Experimental Procedures

Pt(0) and Pt(II) species uptake calculation. ICP was used to determine the concentration (RSD <1%) change of total platinum (0,II, and IV) in solution after adsorption and desorption. ICP was also used to determine the total Pt on the working and counter electrode via aqua regia digestion of the electrode after adsorption – this allowed accurate measure of the fraction of Pt accumulation on the working electrode (vs the counter electrode). XPS analysis was used to determine the oxidation state of accumulated Pt on the working and counter electrodes, and the relative fraction of Pt(II) in a single sample was accurately determined with the Pt(0), Pt(II), and Pt(IV) peak areas. Therefore, The uptake of Pt(II) on both working and counter electrode was calculated using the following equation:

$$q_{WE}^{Pt(II)} = q_{tot}^{Pt} * \left[\frac{\Delta m_{WE}^{Pt}}{\Delta m_{WE}^{Pt}+\Delta m_{CE}^{Pt}} * \frac{A_{WE}^{Pt(II)}}{A_{WE}^{Pt(0)}+A_{WE}^{Pt(II)}+A_{WE}^{Pt(IV)}} + \frac{\Delta m_{CE}^{Pt}}{\Delta m_{WE}^{Pt}+\Delta m_{CE}^{Pt}} * \frac{A_{CE}^{Pt(II)}}{A_{CE}^{Pt(0)}+A_{CE}^{Pt(II)}+A_{CE}^{Pt(IV)}}\right] \quad (1)$$

Where:

q_{tot}^{Pt} is the total uptake of atomic Pt – from ICP

Δm_{WE}^{Pt}, Δm_{CE}^{Pt} are the changes of atomic Pt mass found on the working and counter electrodes after digestion in aqua regia – from ICP

$A_{WE}^{Pt(0)}$, $A_{WE}^{Pt(II)}$, $A_{WE}^{Pt(IV)}$ are the uncoupled oxidation peak areas for Pt(0,II,and IV) observed on the working electrode (same for the counter electrode, CE) – from XPS

Relative selectivity factor calculation for catalysts. The relative selectivity factor, S, is defined as the selectivity of catalyst-ferrocene binding relative to all other possible competing anions in solution and is calculated with the following equation:

$$S = \frac{[comp]_i}{[cat]_i} * \frac{[cat]_f}{[comp]_f} \quad (2)$$

Where:

$[comp]_i$ is the initial concentration of competing ions in solution

$[cat]_i$ is the initial concentration of catalyst species in solution

$[comp]_f$ is concentration of competing ions adsorbed onto the PVF electrode

$[cat]_f$ is the concentration of catalyst adsorbed onto the PVF electrode

Initial concentrations of catalyst and competing ions are known for each system, however the ratio of catalyst to competing ions adsorbed onto the electrode surface was determined with a range of surface and solution techniques: XPS, SEM-EDS, ICP-OES, and TOF-SIMS.

Calculation of the energy factor of electrodeposition versus electrosorption. From Figure S19e, the energy of Speier's catalyst capture was calculated from potentiostatic data as follows:

$E_{-0.6v}$ = 5.179 kJ/g-PVF
$E_{+0.5V}$ = 0.162 kJ/g-PVF

Factor of electrodeposition compared to electrosorption energy:

$$E_{-0.6v} / E_{+0.6V} = 32.0$$

3. Reaction Recycling Procedures

Silane etherification reaction procedure. Speier's catalyst was synthesized by adding 50 mg of hexachloroplatinic acid (CPA) to 1 ml of anhydrous isopropanol and stirred for 30 minutes. Due to the highly hygroscopic nature of dry CPA, it was handled and massed within a glovebox. The 50 g/L Speier's catalyst solution was stored in a UV resistant vial and kept refrigerated to inhibit the chance of degradation. Typical Silane etherification reactant solution consisted of 1 mL triethylsilane (TES) 2 mL ethanol, and 20 mM of tetrabutylammonium perchlorate (TBAP). The concentration of silane within the reactant solution was maintained for all experiments to 2.09 mol/L. The silane etherification reaction was carried out within 3D-printed polypropylene electrochemical cells containing 1.2 mL reactant solution at room temperature with stirring, and the typical reaction time was 30 minutes. The reaction was initialized by one of two methods; the first method was by direct addition of catalyst solution (called the control or initialization reaction), and the second method of reaction initialization was by electrochemical release of catalyst from PVF-CNT electrode (called the experiment or cycled reaction). For a control reaction, either 50 mg-Pt/L Speier's catalyst or 100 mg-Pt/L Karstedt's catalyst (2% Pt in xylene as received) was used. As the Silane etherification reaction progressed, hydrogen gas was released, and bubbles were visibly seen and used as a general heuristic for reaction progress. Reaction products were identified using both H^1NMR, GC-MS, and ESI-LCMS, and reaction yield was calculated from H^1NMR data. For select experiments, reaction kinetics were determined by taking periodic H^1NMR aliquots. NMR data are consistent with literature values *(63)*.

Triethyl ethoxysilane was isolated from the reaction mixture by removing the solvent under reduced pressure and subsequently purified by distillation at 156 mbar. Yielding 3.059 g (38%) $CH_3CH_2OSiEt_3$ as a colorless liquid for a reaction with fresh catalyst, and 1.160 g (35%) for a reaction using recycled catalyst.

$CH_3CH_2OSiEt_3$

H^1 NMR ($CDCl_3$, 500MHz) δ (ppm): 3.65 (q, J = 7.2 Hz, 2H, CH2), 1.15 (t, J = 7.2 Hz, 3H, CH3), 0.93 (t, J = 8.5 Hz, 9H, SiEt), 0.57 (q, J = 8.5 Hz, 6H, SiEt).

HR-ESI m/z: $[M]^+$ calcd. for $C_8H_{20}SiO$, 160.1283; found: 160.1285.

CH_3OSiEt_3

H^1 NMR ($CDCl_3$, 500MHz) δ (ppm): 3.36 (s, 3H, H_3CO-), 0.96 (t, J = 8.0 Hz, 9H, SiCH2CH3) 0.57 (q, J = 8.0Hz, 6H, $SiCH_2CH_3$).

i-$PrOSiEt_3$

H^1 NMR ($CDCl_3$, 500MHz) δ (ppm): 4.12 (septet, J = 6 Hz, 1H), 1.28 (d, J = 6 Hz, 6 H), 1.14 (m, 9H, overlapped with Et_3SiH), 0.75 (m, 6H, overlapped with Et_3SiH).

$CH_3CH_2OSiMe_2Ph$

H^1 NMR ($CDCl_3$, 500MHz) δ (ppm): 7.60-7.55 (m, 2H, Ph), 7.40 – 7.35 (m, 3H, Ph), 3.68 (q, J= 7.14 Hz, 2H, CH_3CH_2), 1.19 (t, J = 7.14 Hz, 3H, CH_3CH_2), 0.39 (s, 6H, SiMe).

Hydrosilylation reaction procedure. Typical hydrosilylation reaction solution consisted of 1 mL TES as the silane, 1 ml of Phenylacetylene as the olefin, 1 ml of acetonitrile as solvent, and 20 mM TBAP as supporting electrolyte and competing ion. Reactant solution was made fresh for each experiment and stored in refrigerator when not in use. Hydrosilylation reactions were carried out in a Teflon-capped amber glass vial (containing 1.2 mL of reactant solution) at 50°C with stirring for 24 hours. Like the silane etherification procedure, hydrosilylation was initiated either by direct addition of catalyst (control reaction) or electrochemical release of captured catalyst from PVF-CNT electrode (cycled reaction). In the case of the control reaction, either 100 ppm Speier's catalyst or 200 ppm Karstedt's catalyst was used. For the cycled reaction, the reaction is first initialized in a 3D-printed electrochemical cell where catalyst is released, and after 30 minutes the

solution is transferred to a Teflon-capped amber glass vial for the remaining 23.5 hours. Reaction products were identified using both H^1 NMR and ESI-LCMS, and reaction yield was calculated from ^1H NMR data. NMR data are consistent with literature values *(64)*.

^1H NMR (500 MHz, $CDCl_3$) δ (ppm): 0.63 (q, J = 7.4 Hz, 6H, CH_2CH_3), 0.97 (t, J = 7.4 Hz, 9H, CH_2CH_3), 6.45 (d, J = 19.3 Hz, 1H, =CH), 6.92 (d, J = 19.3 Hz, 1H, =CH), 7.15-7.19 (m, 1H, Ph-H), 7.22-7.35 (m, 3H, Ph-H), 7.45-7.49 (m, 1H, Ph-H).

ESI m/z: $[M]^+$ calcd. for $C_{14}H_{22}Si$, 218.1491; found: 218.1496

For all hydrosilylation electrochemical recycling experiments, a non-aqueous Ag/Ag+ reference electrode was used. Optimal adsorption and desorption values obtained with reference to Ag/AgCl were translated to non-aqueous reference using the observed standard potential of PVF from CV as an intermediate reference.

Triethyl(phenylvinyl)silane was isolated from the reaction mixture by diluting the reaction mixture with diethylether. The organic phase was washed with water and brine. Afterward, the organic phase was dried with $MgSO_4$ and the solvent was evaporated after filtration. The product was purified by flash chromatography (silica column and Hexane/CH_2Cl_2 gradient). Yielding 810 mg (81%) of a mixture of 78% triethyl(2-phenylvinyl)silane and 22% triethyl(1-phenylvinyl)silane as a slightly yellow liquid for a reaction with fresh catalyst, and 670 mg (67%) of a mixture of 76% triethyl(2-phenylvinyl)silane and 24% triethyl(1-phenylvinyl)silane for a reaction using recycled catalyst.

^1H NMR (CD_2Cl_2, 500MHz) δ (ppm): 7.47 (d), 7.36-7.17 (m), 6.94 (d,), 6.48 (d), 5.89(d), 5.61(d), 1.03 (t), 0.96 (t), 0.71 (q)

HR-ESI m/z: $[M]^+$ calcd. for $C_{14}H_{22}Si$, 218.1491; found: 218.1496.

Wacker oxidation reaction procedure. For control reactions using as-received catalyst, a 12 mL 7:1 (by vol) methanol/water solution containing 10 mM $PdCl_2$, 200 mM $CuCl_2$, 50 mM chlorobenzene and 500 mM 2-vinylnapthalene was prepared. A reactor containing the reactant solution was heated to 80°C, purged and pressurized up to 5 bar with pure oxygen and stirred at 800 rpm. The reaction ended after 8 hr. For an electrochemically recycled reaction, 10.1 mL 7:1 (by vol) methanol/water solution containing 1.49 mM recycled $PdCl_2$, 29.8 mM $CuCl_2$, 50 mM chlorobenzene, 20 mM LiCl and 500 mM 2-vinylnaphthalene was prepared. The reactant solution

was purged with pure oxygen and sealed in a pressure reaction vessel. The stirred vessel was heated to 80°C, purged, and pressurized to 5 bar with pure oxygen. The reaction ended after 24 hr.

For both control reaction and electrochemically recycled reaction, recovery of the catalyst used 6 mL of the product solution (10 mM $PdCl_2$, 200 mM $CuCl_2$, 50 mM chlorobenzene and products in 7:1 (by vol) methanol/water) for adsorption and 6 mL of 20 mM LiCl for desorption. Each 6 mL solution was divided into three 3D-printed electrochemical batch cells (2 mL for each cell). Three PVF-CNT electrodes coated on both sides (0.4 mg of PVF-CNT total) were put together as one working electrode while three carbon paper electrodes were used as one counter electrode along with Ag/AgCl reference. After the catalyst electrosorption at 0.6 V vs. Ag/AgCl for 20 min, the same three electrode configuration containing the three catalyst-laden PVF-CNT electrodes was transferred into 2 mL of 20 mM LiCl in 7:1 (by vol) methanol/water. The adsorbed catalyst was released at 0.1 V vs. Ag/AgCl for 20 min. This adsorption-desorption cycle was repeated 3 times for each 2 mL cell. The ICP-OES showed that 25.8% $PdCl_2$ (20.1 ppm Pd) was recovered from the control reaction solution. To obtain sufficient amount of an isolated product from the electrochemically recycled reaction, the Pt-recovered solution was scaled up by diluting the recovered catalyst solution with 7:1 (by vol) methanol/water solution to make 10.1 mL of 1.49 mM $PdCl_2$. Afterwards, $CuCl_2$, chlorobenzene, and 2-vinylnaphthalene were added to make the reactant solution composition mentioned above.

2-Acetonaphtone was isolated from the reaction mixture by diluting the reaction mixture with diethylether. The organic phase was washed with water and brine. Afterward, the organic phase was dried with $MgSO_4$ and the solvent was evaporated after filtration. The product was purified by flash chromatography (silica column and Hexane/CH_2Cl_2 gradient). Yielding 215 mg (41%) 2-acetonaphtone as a slightly yellow liquid for a reaction with fresh catalyst, and 210 mg (40%) for a reaction using recycled catalyst.

^{1}H NMR (CD_2Cl_2, 500MHz) δ (ppm): 8.48 (s, 1H), 8.01 (m, 2H), 7.91 (m, 2H), 7.52 (m, 2H), 2.70(s, 3H).

HR-ESI m/z: $[M+H]^+$ calcd. for $C_{12}H_{11}O$, 171.0810; found: 171.0809.

Suzuki cross-coupling reaction procedure. 250 mM 4-bromoacetophenone, 350 mM phenylboronic acid, 500 mM sodium acetate, and 250 mM tetrabutylammonium bromate were added to 10:1 (by vol) ethanol/water, and the reactant solution was purged with nitrogen. For

control reactions with as-received catalyst, 0.1mM $PdCl_2(PPh_3)_2$ was added from a 10 mM stock solution in DMF. For electrochemically recycled reactions, a catalyst laden PVF-CNT electrode, reference electrode, and carbon paper counter electrode were added to the Suzuki reactant solution and a constant potential of 0.1V vs Ag/AgCl was applied for 30 minutes. After catalyst was added to reactant solution, a stir bar was added, and the reaction vial was sealed. Timing of the reaction began when the sealed reaction vial was placed in a stirred oil bath at 80°C.

4-Acetylbiphenyl was isolated from the reaction mixture by diluting the reaction mixture with diethylether. The organic phase was washed with water and brine. Afterward, the organic phase was dried with $MgSO_4$ and the solvent was evaporated after filtration. The product was purified by flash chromatography (silica column and Hexane/CH_2Cl_2 gradient). Yielding 120 mg (61%) 4-acetylbiphenyl as a colorless solid for a reaction with fresh catalyst, and 66 mg (67%) for a reaction using recycled catalyst.

^{1}H NMR (CD_2Cl_2, 500MHz) δ (ppm): 8.02 (d, 2H), 7.72 (d, 2H), 7.66 (d, 2H), 7.48 (t, 2H), 7.41 (t, 1H), 2.61(s, 3H).

HR-ESI m/z: $[M+H]^+$ calcd. for $C_{14}H_{13}O$, 197.0966; found: 197.0964.

Catalyst recycling procedure. A catalyst recycling experiment begins with an NMR sample of the reactant solution followed by an initialization reaction where known amount of catalyst is manually pipetted into a reactant mixture. For silane etherification, the reaction is considered complete once hydrogen bubbling completely stops (typ. 5 to 10 minutes), and for hydrosilylation, the reaction is always stopped after 4 hours. Once the initialization reaction was stopped, reaction time was recorded, a second NMR sample was taken, and an ICP sample was taken. The initialization reaction product solution was then added to a three-electrode electrochemical cell where catalyst was adsorbed by an applied potential of +0.5V vs Ag/AgCl to the PVF-CNT electrode for 30 minutes at room temperature and with stirring. After adsorption, the electrodes were removed (PVF-CNT working, carbon paper counter, and reference) and a second ICP sample was taken. A second electrochemical cell was then filled with 1 mL of reactant solution and the electrodes (with catalyst adsorbed) were added. The second reaction, known as the cycled reaction, was initialized by an applied potential of +0.1V vs Ag/AgCl to the PVF-CNT electrode for 30 minutes. After catalyst desorption, a third ICP sample and third NMR sample is taken of the solution once the reaction completes, concluding the experiment. Product identification is done

with ESI-LC-MS and NMR analysis of both initialization reaction and cycled reaction. Reaction yield was determined by quantitatively comparing NMR samples of before and after reaction.

Silane etherification reaction electrode cycling experiment procedure. Electrode durability was tested by running the catalyst recycling procedure (above) where Speier's catalyst is electrochemically recycled in a TES etherification reaction with ethanol. This catalyst recycling experiment was repeated in its entirety multiple times reusing the same PVF-CNT working and carbon paper counter electrodes in each cycle. After the PVF-CNT working and carbon paper counter electrodes were cycled through 7 iterations of catalyst recycle, the electrodes were kept for XPS analysis. To make each recycle test consecutive, only 7 cycles were possible in a single day.

Table S1. Reaction summary for control reactions with fresh catalyst and electrochemically recycled catalyst reactions.

Reaction	Type	Catalyst Concentration (ppm)	Conversion (%)	Reaction Time (hr)	Turnover Number (-)	Turnover Frequency (1/hr)	Isolated Yield (%)
Silane Etherification	Control	52.21	97.5	0.083	5490	65884	38
	recycle	9.27	97.3	0.5	30859	61718	35
Hydrosilylation	control	103.2	97.3	4	2772	693	81
	recycle	25	66.2	12	7785	649	67
Wacker Oxidation	control	1998	100	8	49	6.13	41
	recycle	365	100	24	145	6.05	40
Suzuki Coupling	control	17.18	62.5	8	964	121	61
	recycle	4.82	51.6	24	2837	118	67

Table S2. Electrochemical performance for each catalyst-reaction system.

Reaction	Reactants	Catalyst	Catalyst Uptake (mg/g)	Recovery efficiency (%)	Specific Energy (kJ/g-PVF)	Energy per recovered catalyst (kJ/g-catalyst)
Silane etherification	MeOH-TES	Speier	88	86	0.46	5.18
	IPA-TES	Speier	56	99	0.19	3.40
	DAOH-TES	Speier	64	99	0.34	5.31
	EtOH-DMePhSi	Speier	154	96	0.35	2.28
	EtOH-TES	Speier	116	98	0.20	1.73
	EtOH-TES	Karstedt's	267	67	0.35	1.33
Hydrosilylation	PhA-TES	Speier	136	99	0.57	4.20
	PhA-TES	Karstedt's	279	79	0.66	2.36
Wacker Oxidation	vinylnapthalene-O2	$PdCl_2$	833	92	0.97	1.16
Suzuki cross-coupling	$PhB(OH)_2$-AcPhBr	$PdCl_2(PPh_3)_2$	11.4	96	0.73	64.65

Specific energy of catalyst recycle was calculated from electrochemical data with the following equation:

$$\hat{E}_{PVF} = \frac{\int_{t_i}^{t_f} (E_{we}(t) - E_{ce}(t)) * i(t) * dt}{m_{PVF}}$$

Where,

$\hat{E}_{PVF}$ is the energy of catalyst recycle normalized by the mass of PVF on the working electrode.

$E_{we}(t)$ is the working electrode potential as a function of time from electrochemical data.

$E_{ce}(t)$ is the counter electrode potential as a function of time from electrochemical data.

$i(t)$ is the current as a function of time from electrochemical data.
m_{PVF} is the mass of PVF on the working electrode.
t is time.

Energy per recovered catalyst was calculated with the following equation:

$$\hat{E}_{cat} = \frac{\hat{E}_{PVF}}{q}$$

Where,
$\hat{E}_{cat}$ is the energy of catalyst recycle per mass of catalyst recovered.
$\hat{E}_{PVF}$ is the energy of catalyst recycle normalized by the mass of PVF on the working electrode.
q is catalyst uptake.

4. Technoeconomic Analysis Supplementary Discussion

A comparative technoeconomic analysis of the PVF adsorption system was performed vs competing catalyst recovery techniques, namely distillation and electrodeposition (**Figure 3g** and **Figure S1**). Distillation is the predominant catalyst recovery technique in industry, and electrodeposition is a major metal recovery process in hydrometallurgy. Our system was modeled as a fully continuous separation process (**Figure 3d**), where two electrochemical recycling units operate in alternating fashion. For hydrosilylation using 20 L/min product stream with 100 mg/L of Speier's catalyst as the basis, the cost of redox-mediated electrosorption was conservatively estimated at $49,912/yr (**Table S5**), being the most economical catalyst recovery method by a significant margin. Distillation and electrodeposition had 460% and 660% higher costs, respectively. In addition, electrosorption consumed 99.78% and 99.84% less energy than distillation and electrodeposition respectively. The low energy demand of electrosorption (0.38 kWh) could be easily met with renewable sources, such as single solar cell with a footprint of 60 cm x 60 cm (0.38 m2) *(72)*. Finally, the active catalyst can often destroyed during distillation and electrodeposition, raising the costs (**Table S4**) (26). Synthesizing new Karstedt's catalyst was estimated to cost 271% more per year than the total cost of sourcing PVF, clearly demonstrating the economic advantage of catalyst recycling. In sum, electrochemical recovery of homogeneous catalysts is remarkably superior to current methods from an economic, energy, and sustainability standpoint.

The given constraints for our technoeconomic analysis were the following: Flow rate, V. = 20 L/min; a recovery fraction, X = 0.999; a platinum concentration, C_{Pt} = 100 mg/L (a range of concentrations from 0.1ppm to 1000 ppm was investigate). The PVF-CNT electroadsorption system was modeled with as a Freundlich isotherm using platinum uptake data over a range of concentrations (200 ppm to 6.25 ppm Speier's catalyst) depicted in **Figure 2e** where the complete model with parameters is shown below.

$$q = b * (C_{Pt})^m \qquad where\ m = 0.4389\ and\ b = 16.166\frac{mg}{L}$$

Through discrete iteration of multiple small flow cells connected in series, we found that the model could be simplified to a singular PVF separation unit where uptake is calculated using the log-mean average of the inlet and outlet concentrations.

$$C_{Pt,LM} = -\frac{C_{Pt} * X}{\ln(1-X)} = 14.46\frac{mg}{L}$$

From here total uptake, q_{tot}, was calculated.

$$q_{tot} = b * \left(C_{Pt,LM}\right)^m = 52.22\ \frac{mg}{g}$$

Power consumption was calculated with experimental energy consumption values. Taking an average from the data in **Figure S2e**, **Figure S7**, **FigureS9**, **Figure 3b**, and **Figure 4**, the total energy consumption per cycle, E_{cyc}, was 0.6 kJ/g-PVF.

$$PVF\ energy\ cost = \frac{\dot{V} C_{Pt} X}{q_{tot}} E_{cyc} * \left(0.12\frac{\$}{kWh}\right) = \$402.21\ per\ year$$

The electrode cost was determined from cost estimates of PVF synthesis as the high cost to purchase PVF (>$500/gram) reflects the current lack of demand. A more accurate estimate of large scale PVF cost was determined by scaling up laboratory scale synthesis procedures found in literature *(65)* starting from ferrocene. Chemical pricing of all precursors was done with Sigma Aldrich, and common industrial solvents (ethanol, IPA, chloroform) were priced with their market value. The price of PVF was conservatively estimated at $3.03 per gram of PVF.

Table S3. **PVF Cost estimate data.**

Compound	Units	Quantity	Cost	Unit Cost	Sigma Product ID
Ferrocene	g	5.00	$0.56	0.1124	8.03978
CH_2Cl_2	ml	559.06	$0.29	0.000532	[Market value]
acetyl chloride	ml	2.01	$0.04	0.0181	8222529025
$AlCl_3$	g	3.94	$0.04	0.0112	801081950
methanol	ml	103.83	$0.33	0.00316	[Market Value]
NaBH4	g	3.69	$1.12	0.303	452882-10KG
DMAP	mg	116.97	$0.06	0.000506	107700-5KG
Et_3N	ml	8.02	$0.08	0.00955	471283-200L
methanesulfonyl chloride	ml	0.99	$0.15	0.154	471259-1L
Azobisisobutyronitrile 98%	g	0.02	$0.03	1.33	441090
benzene	ml	4.67	$0.24	0.0515	319953-4L
petroleum ether	ml	186.79	$3.62	0.0193	184519-18L-CS
**Polyvinylferrocene**	**g**	**2.17**	**$6.57**	**$3.03 per gram**	

Electrodes were constructed with equal-mass MWCNT ($0.60 per gram CNT from Cheaptubes). PVF-CNT electrodes were assumed to wear out after 5000 cycles (N_{cyc}) from data in **Figure 5a**, which is a conservative estimation. The yearly electrode replacement cost was calculated as follows:

$$PVF\ electrode\ cost = \frac{\dot{V}C_{Pt}X}{q_{tot}N_{cyc}} * \left(3.03 + 0.60\frac{\$}{g\ PVF}\right) = \$14{,}600.48\ per\ year$$

The cost to make up for unrecovered platinum was calculated with the market value of platinum, $33,210 per kilogram Pt.

$$Pt\ makeup\ cost = \dot{V}C_{Pt}(1-X) * \left(33210\frac{\$}{kg}\right) = \$34{,}910.35\ per\ year$$

Lastly, the energy, electrode, and Pt makeup costs were summed to give the total yearly cost of catalyst recovery: **$49,913.04 per year** for **PVF electrosorption** of either Speier's catalyst or Karstedt's catalyst.

For electrodeposition, experimental and literature values were used to calculate the energy cost of recovering platinum ($254,751.24 per year). Energy consumption at 3 platinum concentrations was modeled and scaled up based on the PVF electrosorption model. The log-mean concentration was used to determine energy consumption. Platinum makeup cost was the same as PVF adsorption, $34,910.35 per year.

Energy consumption for distillation ($183,650.10 per year) was calculated using heat capacity, boiling point temperature, and heat of vaporization values (calculated using Advanced

Chemistry Development (ACD/Labs) Software V11.02) alongside product composition values of hydrosilylation products with NMR. An 80% heating efficiency was given to account for heat lost to surroundings. Unlike electrosorption and electrodeposition, distillation removes all solvent from platinum, but losses in solids handling are accounted by assuming a 0.9999 fractional recovery. Platinum makeup costs were $3,491.03 per year.

Both electrodeposition and distillation result in the destruction of catalyst and recovery of metallic platinum. Therefore, the cost to regenerate Speier's catalyst and Karstedt's catalyst was estimated. First, solid platinum is digested in aqua regia, yielding aqueous chloroplatinic acid solution ($22.55 per kg Pt). The water is boiled off yielding dry chloroplatinic acid salt ($2.70 per kg Pt). Speier's catalyst is formed by adding 66.7 g of chloroplatinic acid to 1L of isopropyl alcohol ($14.15 per kg Pt), and the final cost to regenerate Speier's catalyst was $39.40 per kilogram Pt. To make Karstedt's catalyst, a procedure in literature was followed that called for Speier's catalyst, sodium bicarbonate, and 1,3-divinyltetramethyldisiloxane ($89.33 per kg Pt), and the final cost to regenerate Karstedt's catalyst was $128.73 per kg Pt. For both electrodeposition and distillation, the cost to regenerate Speier's catalyst was $41,417.28 per year and Karstedt's catalyst was $135,320.98 per year.

Table S4. Cost breakdown of catalyst regeneration estimate.

Component	Consumption	Units	Unit cost ($/units)	Cost ($/kg-Pt)
HCl	18.75	L	0.8	15
HNO_3	6.25	L	1.21	7.55
Heat energy	22.5	kWh	0.12	2.70
Isopropanol	11.79	kg	1.2	14.15
		**Speier's catalyst cost ($/kg-Pt):**		**39.40**
Sodium bicarbonate	1376.67	g	0.00025	0.34
1,3-divinyltetramethyldisiloxane	2966.33	g	0.03	88.99
		**Karstedt's catalyst cost ($/kg-Pt):**		**128.73**

Table S5. Technoeconomic cost summary of different recycling methods for Speier's catalyst (SC) and Karstedt's catalyst (KC) given a hydrosilylation product flow rate of 20 L/min and a base platinum catalyst concentration of 100 mg/L.

Catalyst Recycle Method	Power (kW)	Energy Cost ($/yr)	Electrode Replacement Cost ($/yr)	Catalyst regeneration cost ($/yr)	Lost Platinum Makeup Cost ($/yr)	Total Catalyst Recovery Cost ($/yr)
PVF-adsorption	0.38	$402	$14,600	-	$34,910	$49,912
Distillation	173.6	$183,650	-	(SC) $41,417<br>(KC) $135,321	$3,491	(SC) $228,558<br>(KC) $322,462
Electrodeposition	240.8	$254,751	-	(SC) $41,417<br>(KC) $135,321	$34,910	(SC) $331,079<br>(KC) $424,982

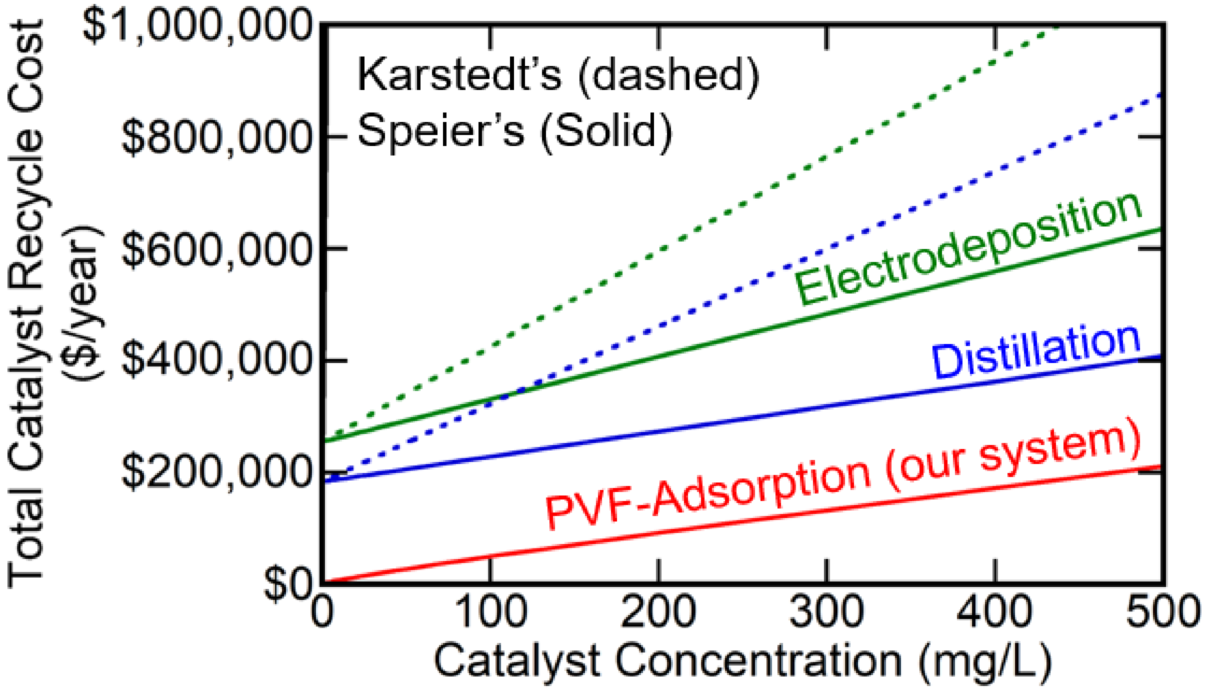

Figure S1. Technoeconomic analysis of PVF-adsorption (red), distillation (blue), and electrodeposition (green). For PVF-adsorption, the total yearly cost of the system linearly decreases with concentration and approaches zero dollars, where distillation and electrodeposition never do.

5. Computational Details

The initial coordinates for all the structures are generated using Avogadro 1.2.0 *(66)* Geometry optimizations are carried out at the ωB97X-D3(BJ)*(67)*/aug-def2-TZVP(68)/CPCM *(69)* level of theory with the def2-ECP(68) pseudopotential for metals, as implemented in ORCA 4.2.1 *(70)* software. No BSSE corrections were computed. The RIJCOSX(34) method with the def2-TZVP auxiliary basis set is used to approximate two-electron integrals and accelerate hybrid DFT calculations. The structures are converged with a tighter convergence threshold for the gradient ($1 \cdot 10^{-4}$ a.u., less than the default value $3 \cdot 10^{-4}$ a.u.), which turned out to be necessary for accurate results. Other ORCA parameters are default. We utilize the CPCM implicit solvent model with the following dielectric constants (default values in ORCA): 36.6 (acetonitrile), 24.3 (ethanol), 40.26 (7:1 methanol/water mixture *(71)*), 29.95 (10:1 ethanol/water mixture *(72)*). Dispersion and cavitation solvation terms are not included in the CPCM model and are expected to cancel out in energy differences. It has been previously shown *(73)* that electrostatics-only solvation yields accurate pK_a values and solvation trends in nonaqueous solvents and solvent mixtures. Multiple initial geometries of non-covalent complexes (formed between Fc^+ and catalysts/anions) are prepared by placing the catalysts/anions around Fc^+ at specific positions where the H atoms of Fc^+ are ~2.5 Å away from halide/oxygen atoms to maximize attractive electrostatic interactions. The lowest-energy structures are used in binding energy calculations.

Gibbs free energies and enthalpies of adduct formation from constituents at 298 K and 1 mol/L standard state are computed by taking energy differences. We employ the quasi-rigid-rotor harmonic-oscillator (qRRHO) model *(74, 75)* to calculate zero-point vibrational energy, enthalpy temperature corrections, and vibrational entropy. Two types of qRRHO models are tested to calculate the contribution of low vibrational modes to vibrational entropy: (i) vibrational modes less than 100 cm^{-1} are scaled to 100 cm^{-1} as proposed by Cramer and Truhlar (qRRHO(T)) *(76)* (ii) contributions of low-lying modes (<100 cm^{-1}) to vibrational entropy are replaced with corresponding rotational entropy contributions (Grimme's approach) (qRRHO(G)) *(75)*. The binding free energies obtained using both models are reported in **Tables S10-S13** Harmonic vibrational frequencies are computed using the same computational setup as during geometry optimization but without the resolution-of-identity (RI) approximation. The rotational and translational contributions to enthalpy and entropy are removed for the systems that are immobilized in the experiment (polymer-bound ferrocenium cation with and without an attached

complex). This procedure is essential for matching experimental binding affinity trends with computations. For comparison, binding energies obtained with and without the removal of translational and rotational contributions are provided in **Table S14-S16**. All binding free energies/enthalpies are corrected by $RTln(24.5)$=1.90 kcal/mol per molecule to account for changing the standard state from gas (1 bar) to liquid solution (1 mol/L). Enthalpies are computed as $H = G + TS$. Thermochemical calculations were carried out using GoodVibes program package (*F1000Research* 2020, **9**(Chem Inf Sci):291).

Local energy decomposition (LED) *(77)* analysis is carried out at the DLPNO-CCSD(T)/ def2-TZVP level of theory using optimized geometries obtained at the DFT level of theory *(78)*. Auxiliary basis sets are generated automatically as implemented in ORCA software *(79)*. The default normal PNO settings are used for LED analysis. DLPNO-CCSD(T) recovers up to 99.9 % of the canonical CCSD(T) correlation energy (with TightPNO settings), and its LED analysis is particularly suitable for studying non-covalent interactions *(80)*. The CPCM solvent model is not supported in LED.

Decomposition of DLPNO-CCSD(T) energy in LED analysis.

The binding energy in LED analysis can be written as:

$$\Delta E = \Delta E_{geo-prep} + \Delta E_{int}$$

where $\Delta E_{geo-prep}$ is the energy required to distort the geometry of monomers from relaxed conformation to that of in complex, ΔE_{int} is the interaction energy, which can be further decomposed into,

$$\Delta E_{int} = E_{el-prep}^{ref} + E_{elstat} + E_{exch} + \Delta E_{int}^{C-T} + \Delta E_{int}^{corr-CCSD}$$

where $\Delta E_{el-prep}^{ref}$ is the energy needed to bring the electronic structure of the separated monomers into the one that is optimal for interaction, E_{elstat} and E_{exch} refers to the electrostatic and exchange interactions between the interacting monomers. ΔE_{int}^{C-T} is the triples correction contribution to binding energy. $\Delta E_{int}^{corr-CCSD}$ is the correlation energy from CCSD calculation which can be represented as

$$\Delta E_{int}^{corr-CCSD} = E_{disp}^{CCSD} + \Delta E_{non-disp}^{CCSD}$$

The term "disp" refers to London dispersion interaction. The rest of the correlation energy is referred as non-dispersion (non-disp) interaction. $\Delta E_{non-disp}^{CCSD}$ can be further decomposed into

$$\Delta E_{non-disp}^{CCSD} = \Delta E_{el-prep}^{corr} + E_{corr}^{CT(X\rightarrow Y)} + E_{corr}^{CT(X\leftarrow Y)}$$

where $\Delta E_{el-prep}^{corr}$ refers to dynamic electronic preparation energy and is included with non-dispersion energy in this work and E_{corr}^{CT} refers to charge transfer interaction between monomer X and Y, arising from double excitations.

Table S6. Various catalytic systems studied in this work and their respective solvent medium with their respective dielectric constant.

	Species involved in interaction with Fc^{+}	Solvent
Spier catalyst system	$[PtCl_3(C_3H_6)]^-$<br>$[PtCl_6]^{2-}$<br>$[ClO_4]^-$	Acetonitrile (ε=36.6)<br>Ethanol (ε=24.3)
Wacker catalyst system	$[PdCl_3(CH_2CHC_6H_5)]^-$<br>$[PdCl_4]^{2-}$<br>Cl^-	7:1 Methanol/Water mixture (ε=40.26)
Suzuki coupling catalyst system	$[PdCl_2(PPh_3)_2]^{2-}$<br>$[PdCl(PPh_3)_2]^-$<br>$[PdBr_2(PPh_3)_2]^{2-}$<br>$[PdBr(PPh_3)_2]^-$<br>Cl^-<br>Br^-	10:1 Ethanol/Water mixture (ε=29.95)

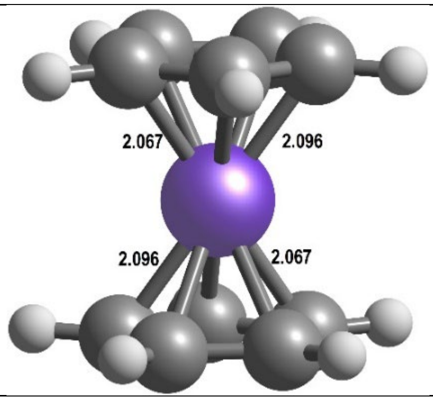

Fc^+ - Front view

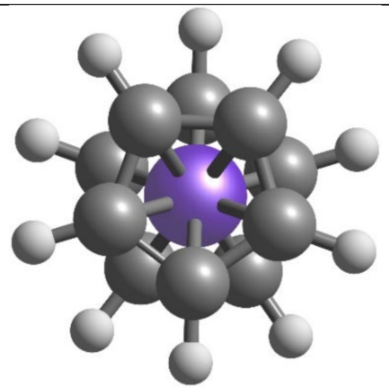

Fc^+ - Top view

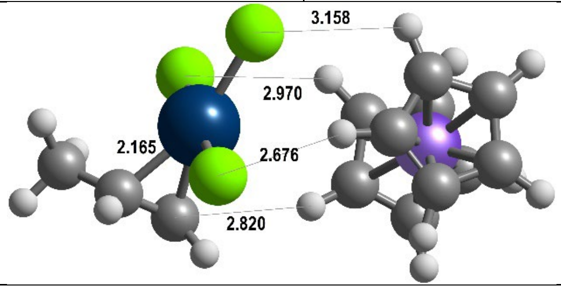

Fc^+ - $[PtCl_3(C_3H_6)]^-$

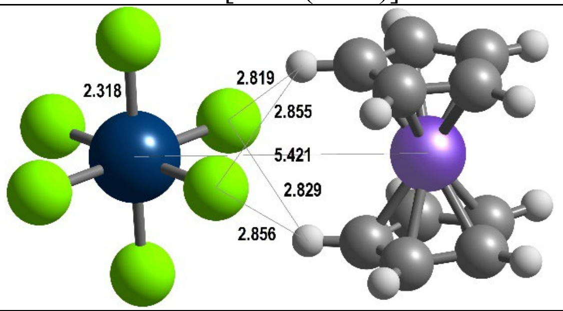

Fc^+ - $[PtCl_6]^{2-}$

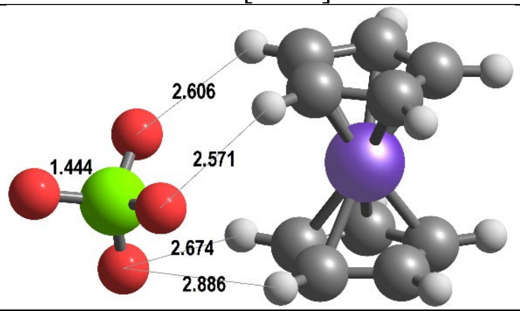

Fc^+ - $[ClO_4]^-$

Figure S2: Geometries of Fc^+ with bound Speier's catalyst in an anionic form, its most probable reactive form with a bound alkene substrate, and a perchlorate anion.

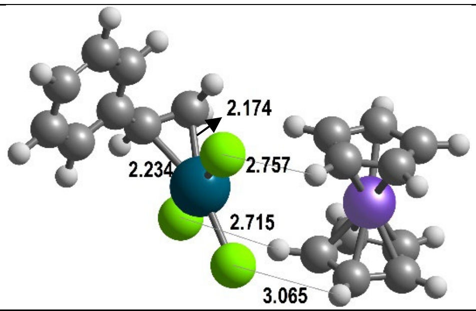

Fc^+ - $[PdCl_3(CH_2CHC_6H_5)]^-$

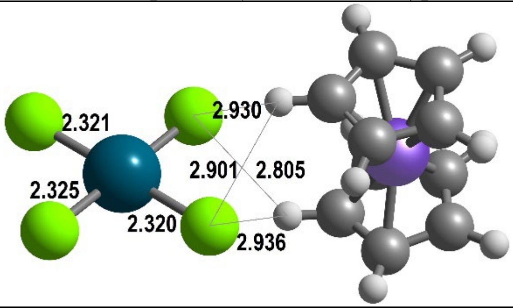

Fc^+ - $[PdCl_4]^{2-}$

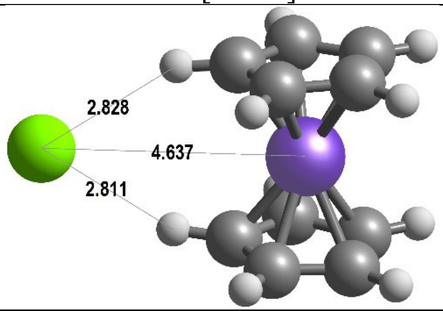

Fc^+ - Cl^-

Figure S3: Geometries of Fc+ with bound Wacker catalyst, its reactive form with the bound substrate, and chloride anion.

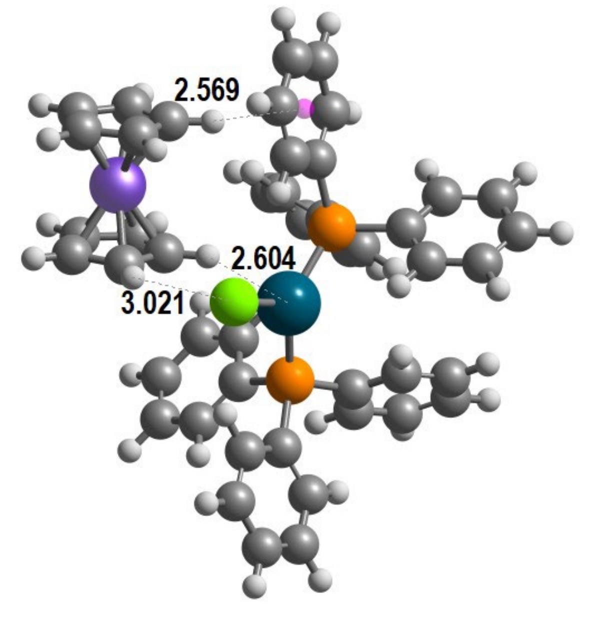

Fc^{+} - $[PdCl(PPh_3)_2]^-$

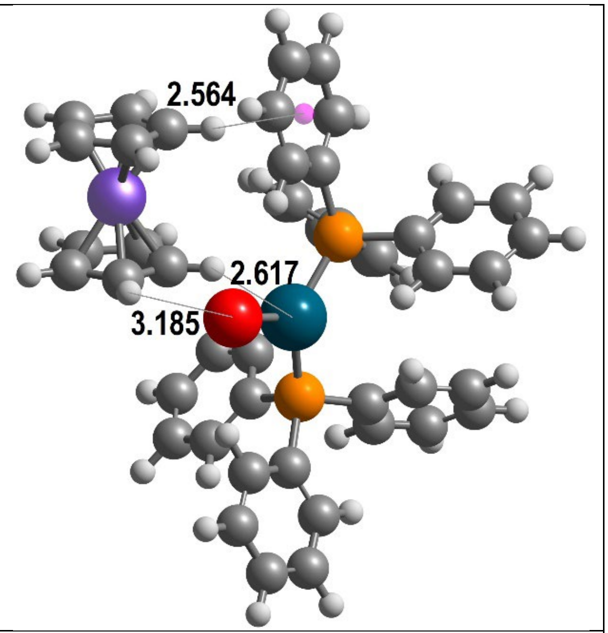

Fc^{+} - $[PdBr(PPh_3)_2]^-$

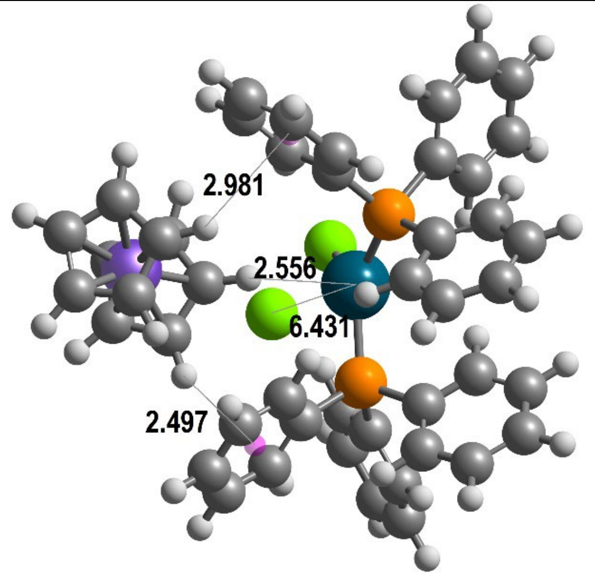

Fc^{+} - $[PdCl_2(PPh_3)_2]^-$

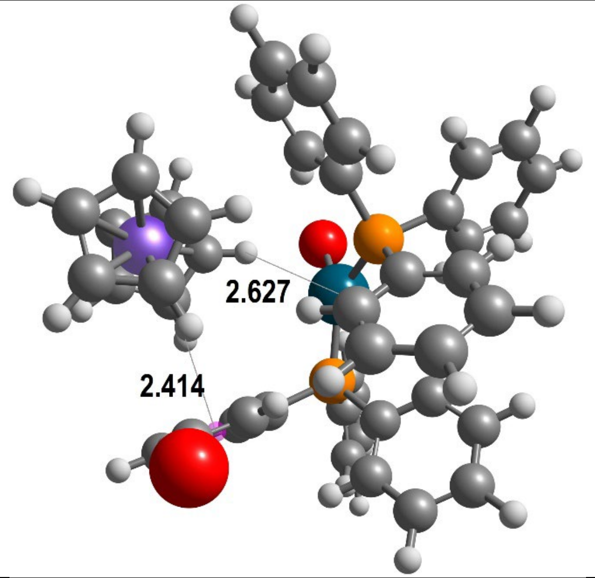

Fc^{+} - $[PdBr_2(PPh_3)_2]^-$

Figure S4: Geometries for Suzuki coupling catalysts binding with ferrocenium.

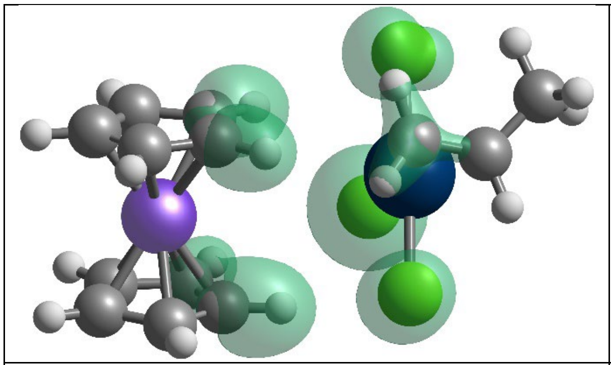

Fc^{+} - $[PtCl_3(C_3H_6)]^{-}$

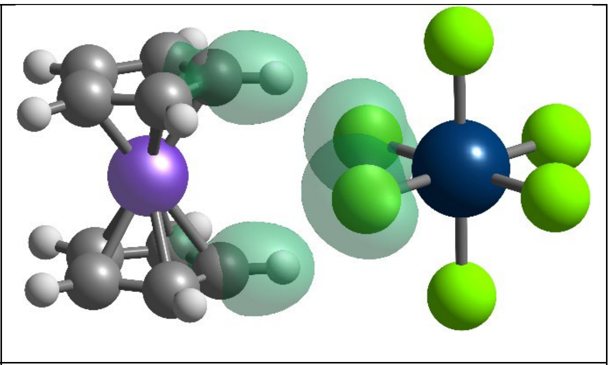

Fc^{+} - $[PtCl_6]^{2-}$

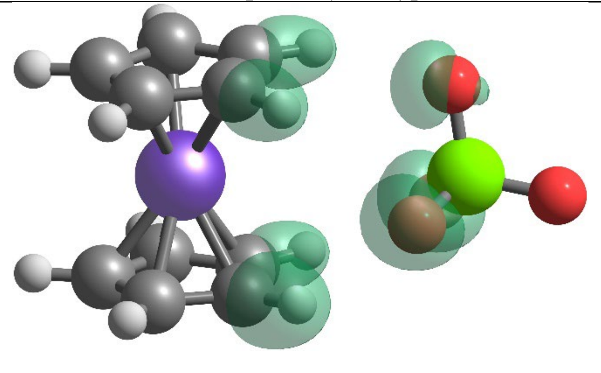

Fc^{+} - $[ClO_4]^{-}$

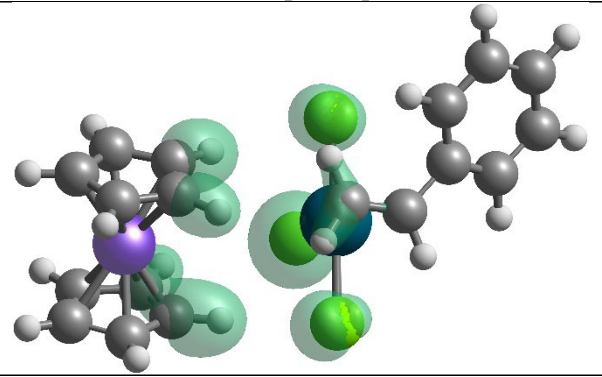

Fc^{+} - $[PdCl_3(CH_2CHC_6H_5)]^{-}$

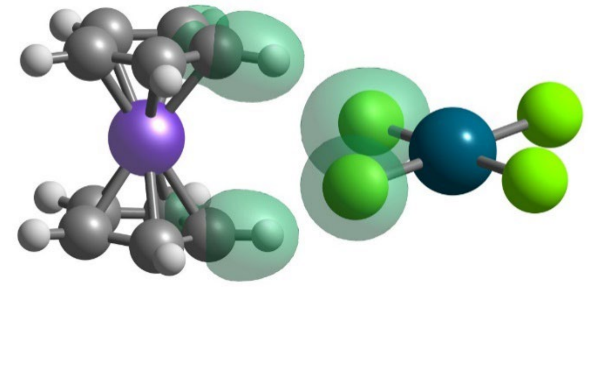

Fc^{+} - $[PdCl_4]^{2-}$

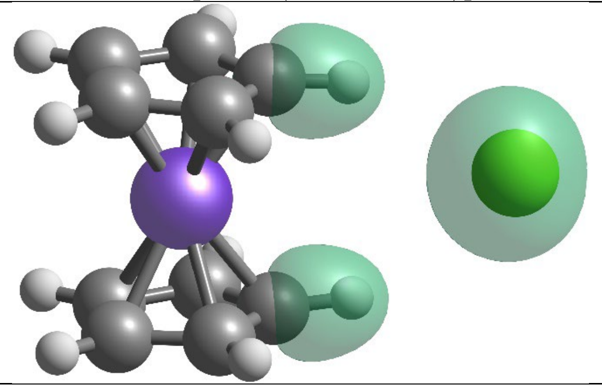

Fc^{+} - Cl^{-}

Figure S5. Dispersion energy density contour diagram for the interaction of Ferrocenium cation with $[PtCl_3(C_3H_6)]^{-}$, $[PtCl_6]^{2-}$, $[ClO_4]^{-}$, $[PdCl_3(CH_2CHC_6H_5)]^{-}$, $[PdCl_4]^{2-}$ and Cl^{-}.

Table S7. Binding free energy and enthalpy of Fc^+ with spier catalyst system (kcal/mol).

	ΔG (Acetonitrile)	ΔH (Acetonitrile)	ΔG (Ethanol)	ΔH (Ethanol)
Fc^+ - $[PtCl_3(C_3H_6)]^-$	0.146	-8.004	-1.149	-9.646
Fc^+ - $[PtCl_6]^{2-}$	0.976	-8.365	-1.517	-10.731
Fc^+ - $[ClO_4]^-$	2.460	-5.332	1.748	-6.862

Table S8. Binding free energy and enthalpy of Fc^+ with wacker catalyst system (kcal/mol).

	ΔG (Methanol/Water)	ΔH (Methanol/Water)
Fc^+ - $[PdCl_3(CH_2CHC_6H_5)]^-$	1.831	-8.700
Fc^+ - $[PdCl_4]^{2-}$	1.664	-8.328
Fc^+ - Cl^-	4.198	-5.547

Table S9. Binding free energy and enthalpy of Fc^+ with wacker catalyst system (kcal/mol).

	ΔG (Ethanol/Water)	ΔH (Ethanol/Water)
Fc^+ - $[PdCl_2(PPh_3)_2]^{2-}$	-5.857	-13.855
Fc^+ - $[PdCl(PPh_3)_2]^-$	-7.398	-16.016
Fc^+ - $[PdBr_2(PPh_3)_2]^{2-}$	-12.199	-14.408
Fc^+ - $[PdBr(PPh_3)_2]^-$	-15.443	-16.487
Fc^+ - Cl^-	-1.915	-5.111
Fc^+ - Br^-	-0.377	-5.834

Table S10. Local energy decomposition (LED) of interaction energy between Fc+ and catalysts at DLPNO-CCSD(T)/def2-TZVP level of theory. For comparison, the binding energy computed at DLPNO-CCSD(T) and DFT level of theory with the same basis set are also provided.

Decomposition of Binding Energy (kcal/mol)										
	$E_{geo-prep}$	$E_{el-prep}^{ref}$	E_{elstat}	E_{exch}	E_{disp}^{CCSD}	$\Delta E_{non-disp}^{CCSD}$	ΔE_{int}^{C-T}	ΔE (Total Binding Energy)	$\Delta E^{CCSD(T)} = E_{dimer} - \sum E_{monomer}$	$\Delta E_{\omega B97X-D3BJ}^{DFT}$ (without solvation correction)
Fc^+ - $[PtCl_3(C_3H_6)]^-$	0.19	1.13	-22.75	-6.82	-7.70	-22.70	-0.54	-59.19	-59.68	-63.99
Fc^+ - $[PtCl_6]^{2-}$	0.88	1.49	-31.68	-7.814	-7.77	-19.53	-0.59	-65.01	-65.91	-77.14
Fc^+ - $[ClO_4]^-$	0.94	1.27	-97.61	-4.10	-4.69	-15.61	-0.24	-119.38	-120.01	-134.95
Fc^+ - $[PdCl_3(CH_2CHC_6H_5)]^-$	0.14	1.12	-23.37	-6.84	-7.13	-24.18	-0.59	-60.85	-61.15	-76.82
Fc^+ - $[PdCl_4]^{2-}$	0.98	1.76	-28.92	-7.93	-5.29	-18.38	-0.49	-58.29	-58.99	-68.31
Fc^+ - Cl^-	1.01	1.59	-103.96	-4.12	-2.20	-16.88	-0.33	-124.89	-125.39	-138.10

*The geometries used for energy decomposition and CCSD(T) calculations are obtained at DFT level of theory.

Table S11. Binding free energy of Fc^+ with spier catalyst system (kcal/mol) with qRRHO(T) and qRRHO(G) models.

	$\Delta G_{qRRHO(T)}$ (Acetonitrile)	$\Delta G_{qRRHO(G)}$ (Acetonitrile)	$\Delta G_{qRRHO(T)}$ (Ethanol)	$\Delta G_{qRRHO(G)}$ (Ethanol)
Fc^+ - $[PtCl_3(C_3H_6)]^-$	0.146	3.172	-1.149	2.877
Fc^+ - $[PtCl_6]^{2-}$	0.976	4.935	-1.517	3.561
Fc^+ - $[ClO_4]^-$	2.460	5.122	1.748	3.873

Table S12. Binding free energy of Fc^+ with wacker catalyst system (kcal/mol) with qRRHO(T) and qRRHO(G)models.

	$\Delta G_{qRRHO(T)}$ (Methanol/Water)	$\Delta G_{qRRHO(G)}$ (Methanol/Water)
Fc^+ - $[PdCl_3(CH_2CHC_6H_5)]^-$	1.831	2.140
Fc^+ - $[PdCl_4]^{2-}$	1.664	2.877
Fc^+ - Cl^-	4.198	1.971

Table S13. Binding free energy of Fc^+ with wacker catalyst system (kcal/mol) with qRRHO(T) and qRRHO(G)models.

	$\Delta G_{qRRHO(T)}$ (Ethanol/Water)	$\Delta G_{qRRHO(G)}$ (Ethanol/Water)
Fc^+ - $[PdCl_2(PPh_3)_2]^{2-}$	-5.857	-2.339
Fc^+ - $[PdCl(PPh_3)_2]^-$	-7.398	-4.215
Fc^+ - $[PdBr_2(PPh_3)_2]^{2-}$	-12.199	-8.977
Fc^+ - $[PdBr(PPh_3)_2]^-$	-15.443	-11.648
Fc^+ - Cl^-	-1.915	0.251
Fc^+ - Br^-	-0.377	2.377

Table S14. Binding free energy of Fc^+ with Speier catalyst system (kcal/mol) with and without the removal of translational and rotational energies and entropies for bound systems.

	$\Delta G_{qRRHO(T)}$ (without trans + rot) (Acetonitrile)	$\Delta G_{qRRHO(T)}$ (with trans+rot) (Acetonitrile)	$\Delta G_{qRRHO(T)}$ (without trans + rot) (Ethanol)	$\Delta G_{qRRHO(T)}$ (with trans+rot) (Ethanol)
Fc^+ - $[PtCl_3(C_3H_6)]^-$	0.146	2.741	-1.149	3.149
Fc^+ - $[PtCl_6]^{2-}$	0.976	3.872	-1.517	2.518
Fc^+ - $[ClO_4]^-$	2.460	3.876	1.748	4.797

Table S15. Binding free energy of Fc^+ with Wacker catalyst system (kcal/mol) with and without the removal of translational and rotational energies and entropies for bound systems.

	$\Delta G_{qRRHO(T)}$ (without trans + rot) (Methanol/Water)	$\Delta G_{qRRHO(T)}$ (with trans+rot) (Methanol/Water)
Fc^+ - $[PdCl_3(CH_2CHC_6H_5)]^-$	1.831	4.682
Fc^+ - $[PdCl_4]^{2-}$	1.664	6.632
Fc^+ - Cl^-	4.198	2.460

Table S16. Binding free energy of Fc^+ with Suzuki catalyst system (kcal/mol) with and without the removal of translational and rotational energies and entropies for bound systems

	$\Delta G_{qRRHO(T)}$ (without trans + rot) (Ethanol/Water)	$\Delta G_{qRRHO(T)}$ (with trans+rot) (Ethanol/Water)
Fc^+ - $[PdCl_2(PPh_3)_2]^{2-}$	-5.857	-1.596
Fc^+ - $[PdCl(PPh_3)_2]^-$	-7.398	-2.997
Fc^+ - $[PdBr_2(PPh_3)_2]^{2-}$	-12.199	-7.854
Fc^+ - $[PdBr(PPh_3)_2]^-$	-15.443	-10.853
Fc^+ - Cl^-	-1.915	-1.124
Fc^+ - Br^-	-0.377	0.857

Table S17. Decomposition of interaction energy (in percentage) into meaningful energy components relevant to non-covalent interaction between Fc^+ and catalysts at DLPNO-CCSD(T)/def2-TZVP level of theory.

	Decomposition of Interaction Energy in %			
	Electrostatic	Exchange/Pauli	Dispersion	Non-Dispersion
Fc^+ - $[PtCl_3(C_3H_6)]^-$	38.4	11.5	13.0	38.4
Fc^+ - $[PtCl_6]^{2-}$	48.7	12.0	11.95	30.0
Fc^+ - $[ClO_4]^-$	81.8	3.4	3.9	13.0
Fc^+ - $[PdCl_3(CH_2CHC_6H_5)]^-$	38.4	11.2	11.7	39.7
Fc^+ - $[PdCl_4]^{2-}$	49.6	13.6	9.1	31.5
Fc^+ - Cl^-	83.2	3.3	1.8	13.5

Table S18. Dispersion and charge-transfer interaction energy between Fc^+ and catalysts, obtained from LED analysis.

	E_{disp}^{CCSD}	$E_{corr}^{CT(Fc^+\rightarrow X)}$	$E_{corr}^{CT(Fc^+\leftarrow X)}$
Fc^+ - $[PtCl_3(C_3H_6)]^-$	-7.70	-0.53	-5.69
Fc^+ - $[PtCl_6]^{2-}$	-7.77	-0.31	-9.67
Fc^+ - $[ClO_4]^-$	-4.69	-0.08	-5.15
Fc^+ - $[PdCl_3(CH_2CHC_6H_5)]^-$	-7.13	-0.36	-6.65
Fc^+ - $[PdCl_4]^{2-}$	-5.29	-0.52	-6.70
Fc^+ - Cl^-	-2.20	-0.07	-7.59

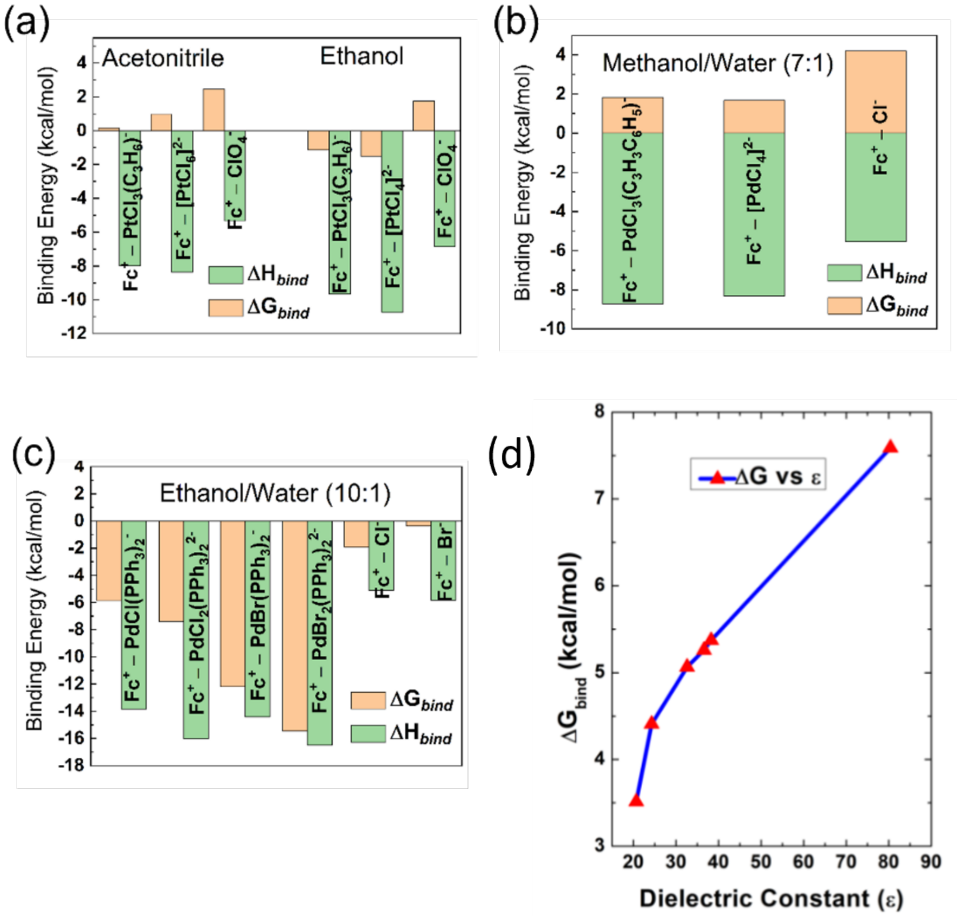

Figure S6: Binding energy of Fc^+ with (a) Speier's catalyst system (b) Wacker catalyst system (c) Suzuki coupling catalyst system. (d) Binding energy of Fc^+ with Speier's catalyst with respect to solvent dielectric constant.

6. Additional SEM/EDS Imagery

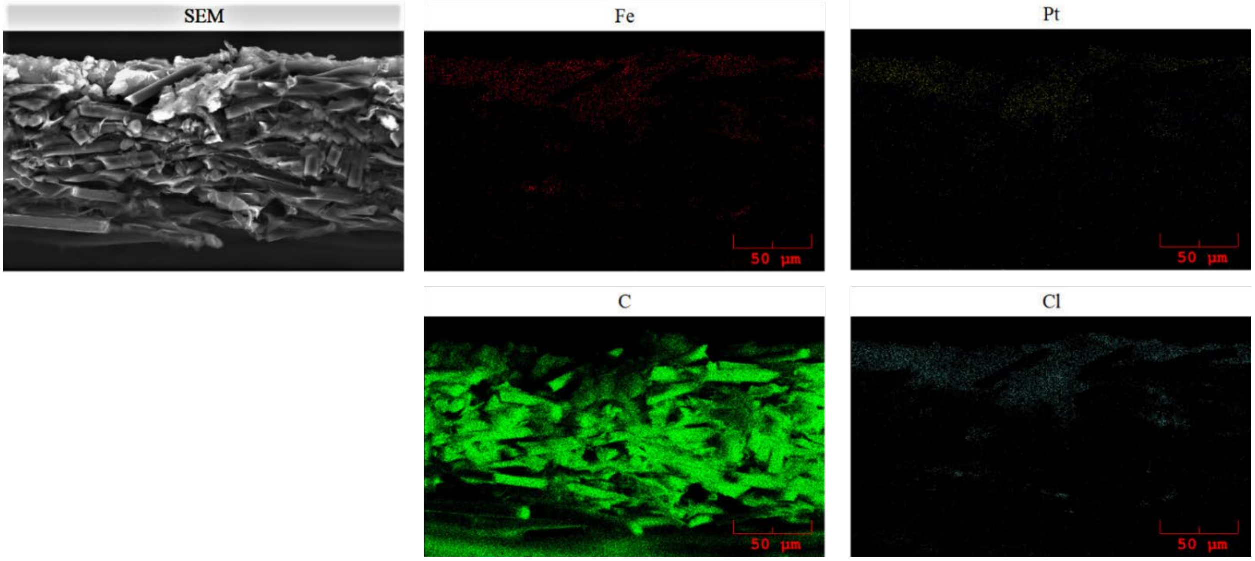

Figure S7. SEM/EDS map of cross section of PVF-CNT electrode. The side coated with PVF-CNT (top) shows more Pt.

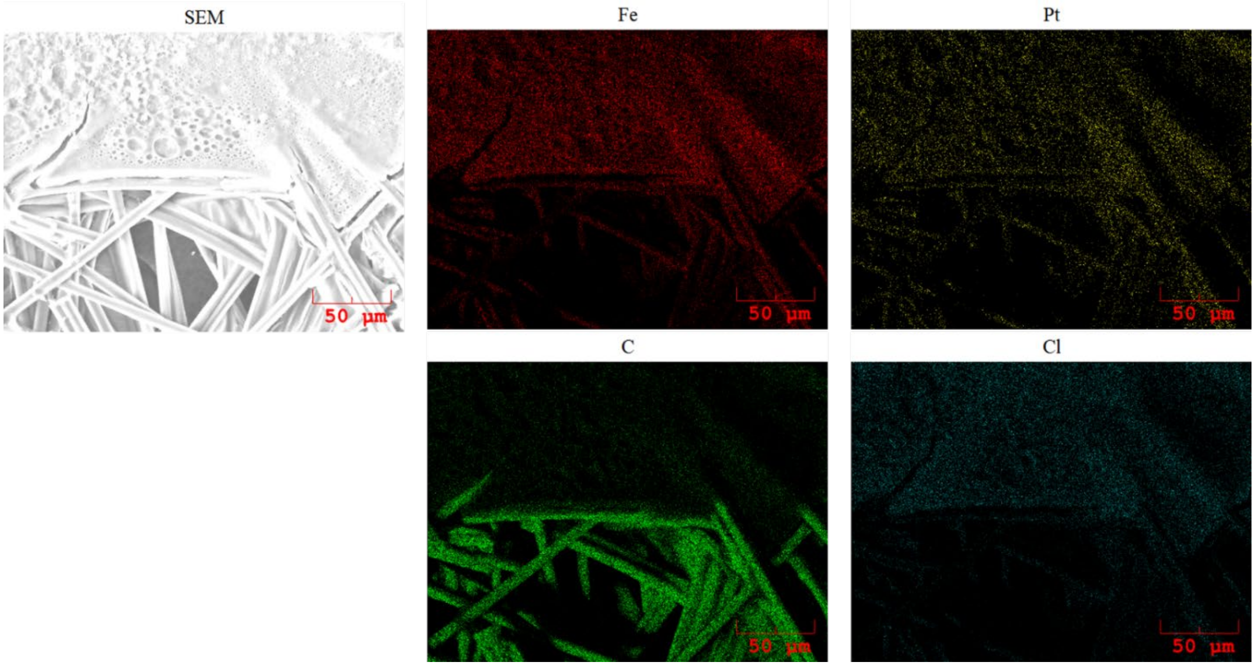

Figure S8. SEM EDS map of PVF-CNT electrode at the coating boundary.

Table S19. EDS elemental analysis of PVF-CNT working electrode after adsorption of 50ppm Karstedt's catalyst from silane etherification products (triethylsilane and ethanol).

Element Line	Net Counts	Weight %	Atom %
C K	35470	68.1	85.5
O K	1353	6.7	6.3
F K	147	0.5	0.4
Si K	1940	1.4	0.7
Cl K	10076	7.7	3.3
Fe K	5753	13.6	3.7
Pt M	1595	2.0	0.2
Total		100.0	100.0

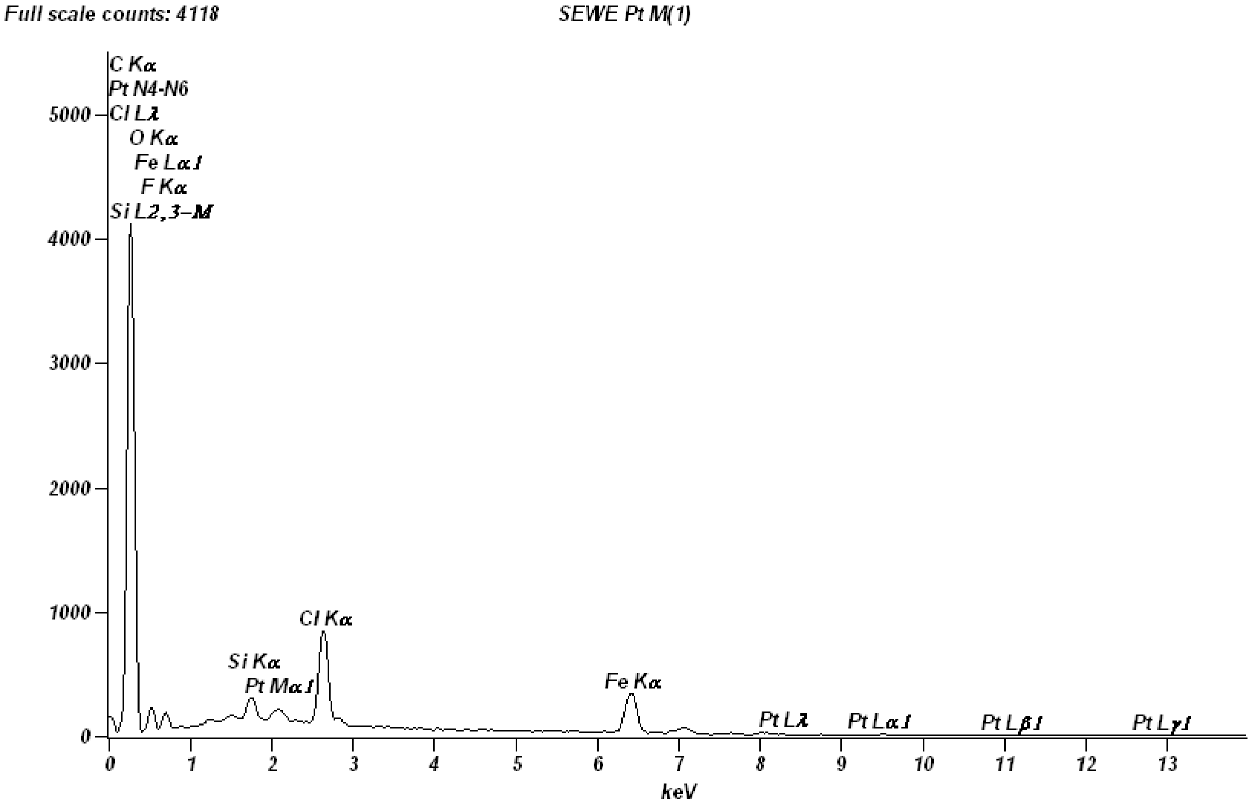

Figure S9. EDS spectrum of PVF-CNT electrode after adsorption of Karstedt's catalyst from silane etherification products.

Table S20. EDS elemental analysis of PVF-CNT working electrode after adsorption of 50ppm Karstedt's catalyst from hydrosilylation products (triethylsilane and phenylacetylene).

Element Line	Net Counts	Weight %	Atom %
C K	35470	68.1	85.5
O K	1353	6.7	6.3
F K	147	0.5	0.4
Si K	1940	1.4	0.7
Cl K	10076	7.7	3.3
Fe K	5753	13.6	3.7
Pt M	1595	2.0	0.2
Total		100.0	100.0

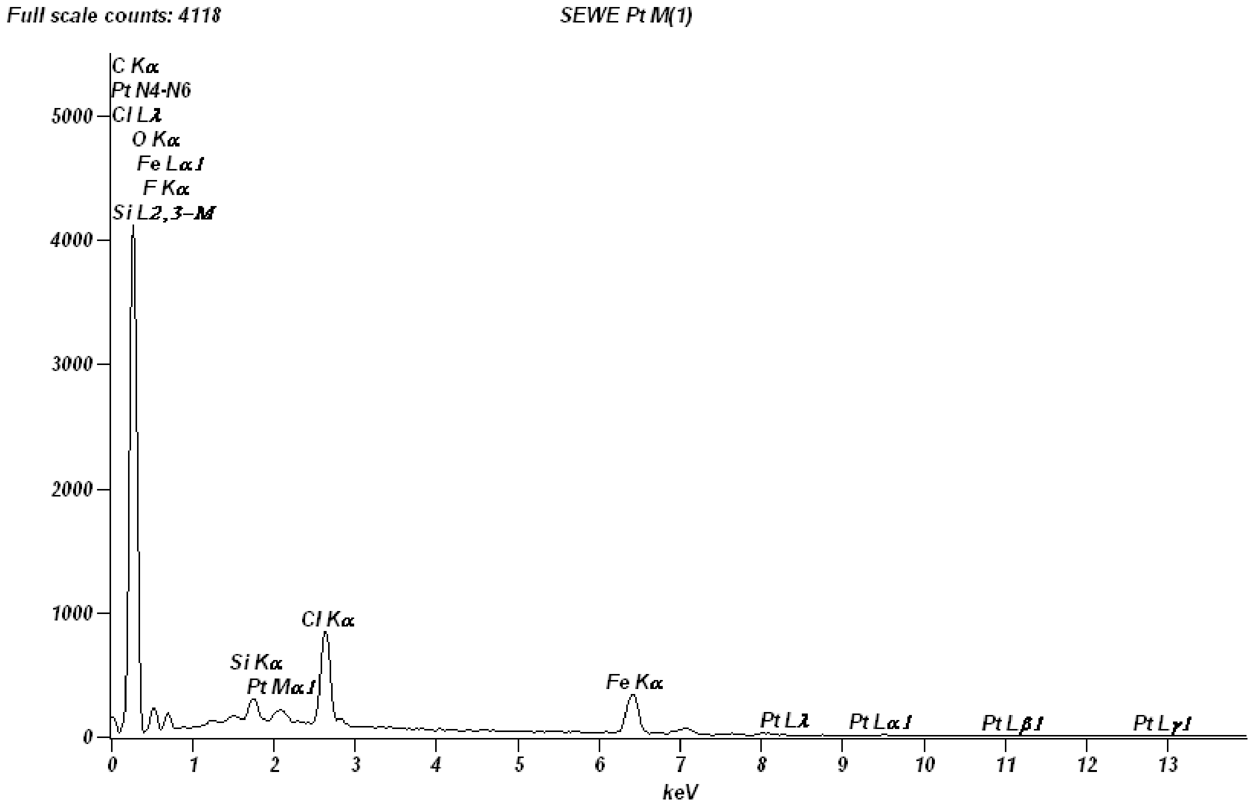

Figure S10. **EDS spectrum of PVF-CNT electrode after adsorption of Karstedt's catalyst from hydrosilylation products.**

7. Additional XPS Data

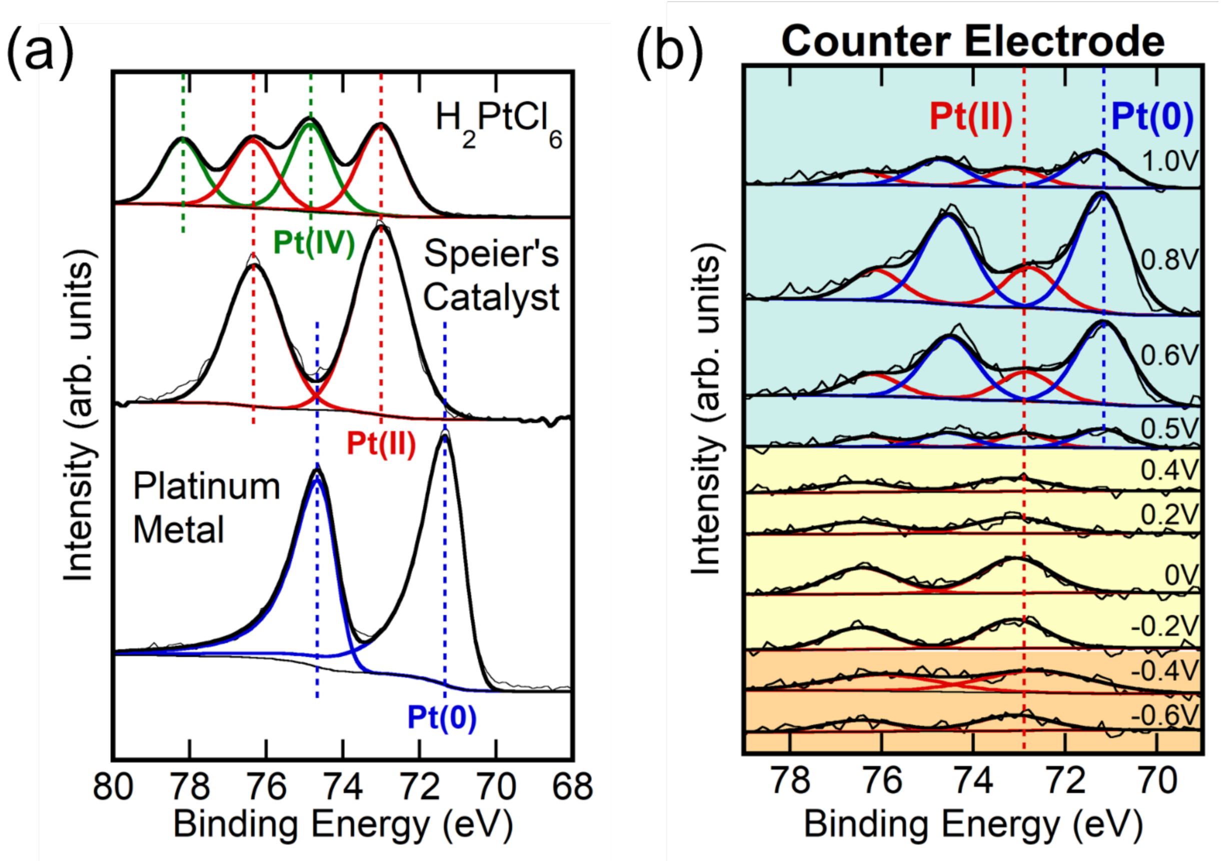

Figure S11. Platinum XPS 4f Spectra. (a) Platinum XPS 4f spectra of various platinum species controls. Chloroplatinic acid (top) shows a Pt(IV) peak at 75eV and a Pt(II) peak at 73eV due to light degradation. Speier's catalys shows only a Pt(II) peak, and pure platinum metal shows an asymmetrical Pt(0) peak at 71eV. (b) Platinum XPS 4f spectra of carbon paper electrodes after adsorption over a range of applied potentials (applied potential is the PVF-CNT working electrode versus Ag/AgCl reference). Electrodeposited Pt(0) begins to form at potentials above 0.5V (blue region). Only trace Pt(II) is found at a working potential below 0.4V (yellow and orange regions).

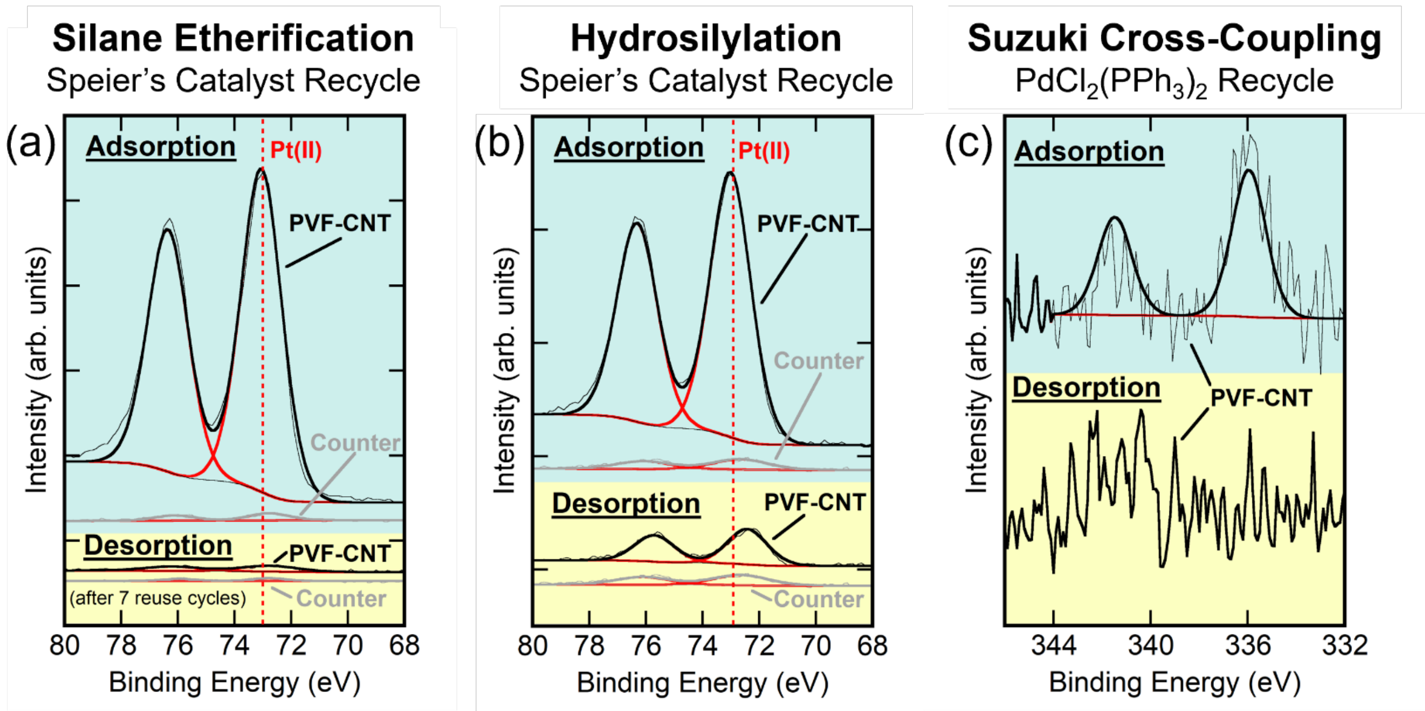

Figure S12. XPS spectra of PVF-CNT electrodes (black lines) and counter electrodes (grey lines) after adsorption (blue region) and desorption (yellow region) for the following reaction-catalyst systems: (a) Speier's catalyst Silane etherification of Triethylsilane and ethanol. Note that XPS analysis was done on electrodes after 7 consecutive recycle procedures. (b) Speier's catalyst Hydrosilylation of Triethylsilane and phenylacetylene. (c) $PdCl_2(PPh_3)_2$ catalyst Suzuki cross-coupling of phenylboronic acid and 4-bromoacetophenone.

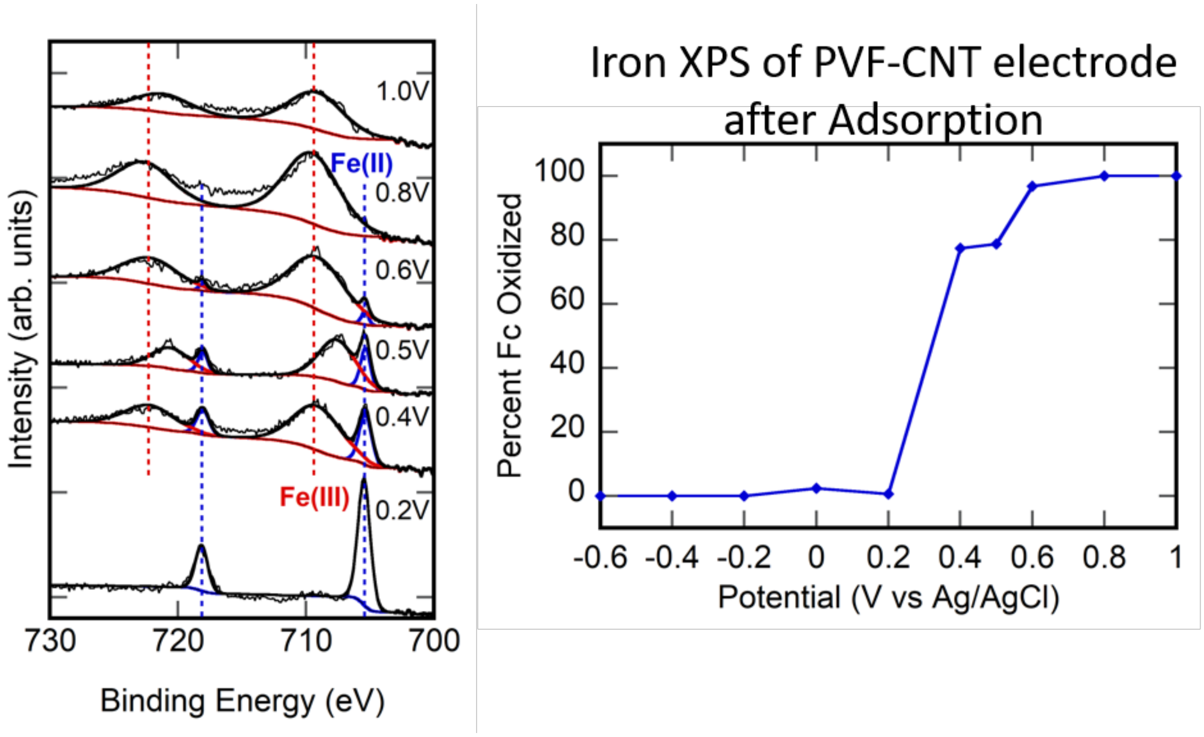

Figure S13. Raw Fe XPS spectra and its interpretation. a) Iron XPS spectra of PVF-CNT electrodes after adsorption at various potentials (0.2V to 1V vs Ag/AgCl). The only Iron source on electrodes is the Fe center of ferrocene. Fe(II) (seen at 705eV) represents reduced ferrocene, and Fe(III) (seen at 709eV) represents oxidized ferrocenium. b) quantitative representation of the iron oxidation state from XPS data for PVF-CNT electrodes after adsorption at various potentials. Iron rapidly oxidizes beyond 0.4V vs Ag/AgCl.

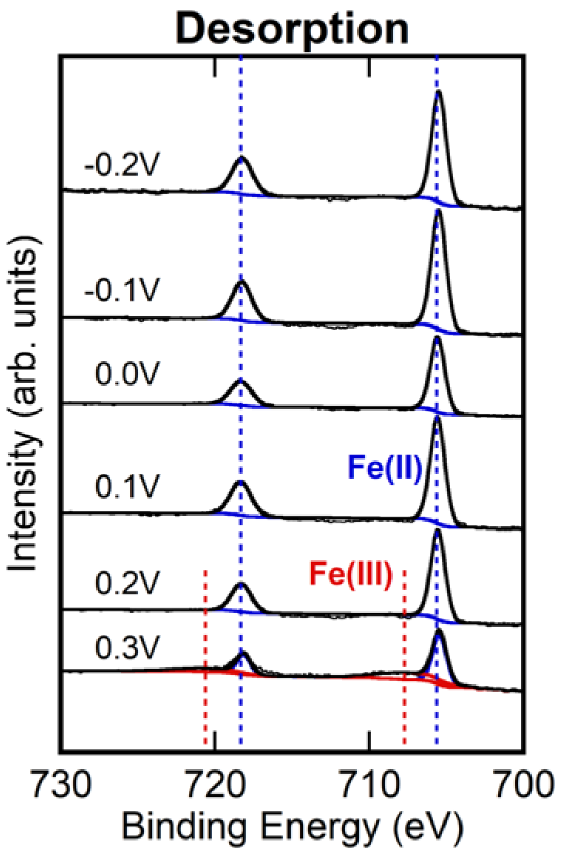

Figure S14. Iron XPS spectra of PVF-CNT working electrodes after desorption of Speier's catalyst in ethanol. Fe(II) peak at 705eV represents fully reduces ferrocene sites, and Fe(III) peak at 708eV represents oxidized ferrocenium. Only faint trace of ferrocenium is observed at +0.3V vs Ag/AgCl, and full ferrocene reduction is observed for all other potentials.

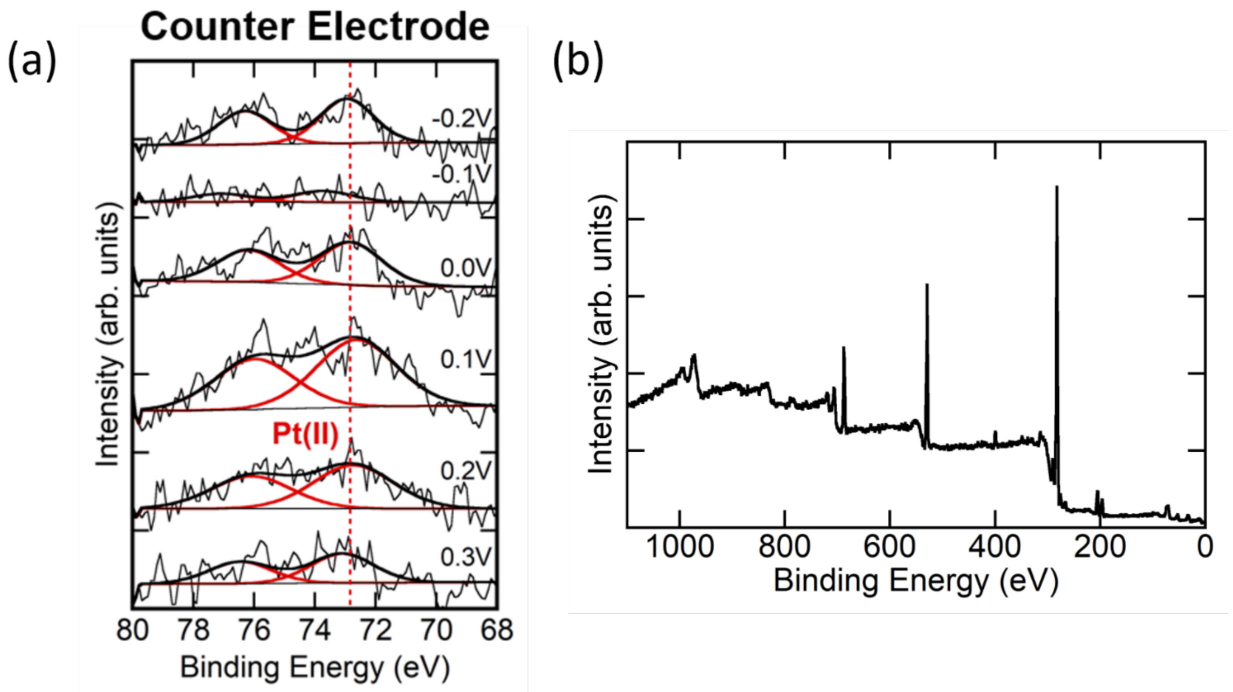

Figure S15. Platinum XPS analysis of the counter electrode. (a) 4f XPS spectra for the carbon paper counter electrodes after Speier's catalyst desorption at various desorption potentials (adsorption was at +0.5V vs Ag/AgCl). Pt(II) is the only species present meaning no Pt reduction occurred regardless of potential. The Working electrode at +0.3V is the only sample that shows a strong Pt(II) signal indicating incomplete release of adsorbed catalyst. (b) XPS Survey spectra of PVF-CNT electrode after adsorption of Speier's catalyst in Hydrosilylation.

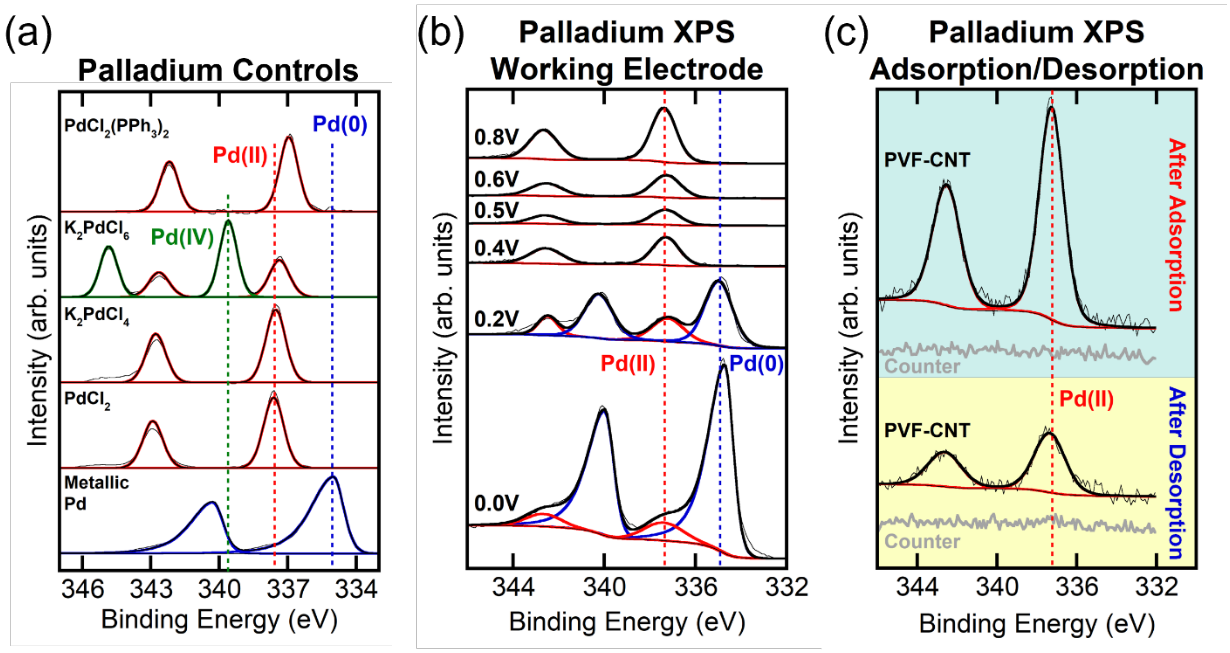

Figure S16. Palladium XPS spectra. (a) dried palladium salt controls. Metallic Pd shows an asymmetrical peak at 335 eV corresponding to Pd(0). PdCl2, K2PdCl4 and PdCl2(PPh3)2 showed a peak at 337.5 eV corresponding to Pd(II). K2PdCl6 showed a peak at 339.5 eV corresponding to Pd(IV). (b) Palladium XPS spectra of PVF-CNT working electrodes after adsorption over a range of applied potentials (vs Ag/AgCl). Above 0.4V, only Pd(II) is observed on the electrode. Below 0.2V, electrodeposited Pd(0) begins to appear. (c) Comparison of both PVF-CNT working electrode (black) and counter electrode (grey) after adsorption (blue background) at 0.6V and desorption (yellow background) a t+0.1V. No Pd was ever observed on the carbon paper counter electrode, and only Pd(II) was observed after adsorption and desorption.

8. Additional Electrochemical Data

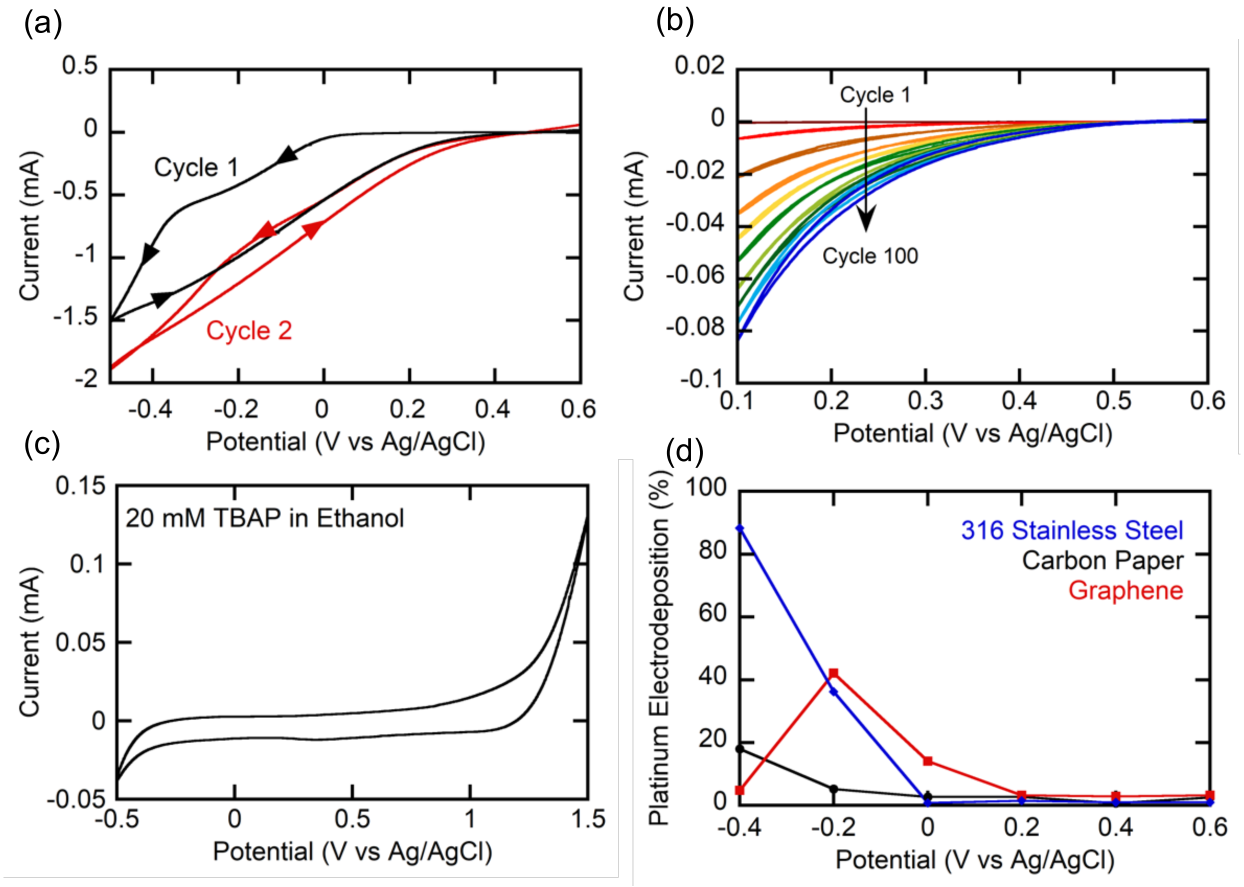

Figure S17. Electrochemical stability tests and controls. (a) Cyclic voltammogram of chloroplatinic acid (5 mM) in water with 20 mM NaClO4 supporting electrolyte. The first cycle shows the onset of Pt electrodeposition at -0.2V and cycles in a counterclockwise "rotation" signifying deposited Pt on electrode surface catalyzes further Pt electrodeposition. This is further evident on subsequent cycles where the overpotential of platinum deposition is lowered and occurs around +0.2V vs Ag/AgCl. (b) Cyclic voltammogram of 5 mM chloroplatinic acid in water with 20 mM NaClO4 supporting electrolyte. A narrow potential window is chosen (between 0.1V and 0.6V vs Ag/AgCl) and cycled 100 times. Platinum electrodeposition is significantly inhibited by the narrow potential window. (c) Voltammogram of ethanol stability window. 50 mV/s scan rate with carbon paper working and counter electrodes. Ethanol electro-degradation is observed at potential >1.3V and <-0.4V vs Ag/AgCl. (d) Current collector material study where the goal was to find a material that inhibits platinum electrodeposition over the widest range of potentials. Toray 030 carbon paper with 5% Teflon coating was chosen.

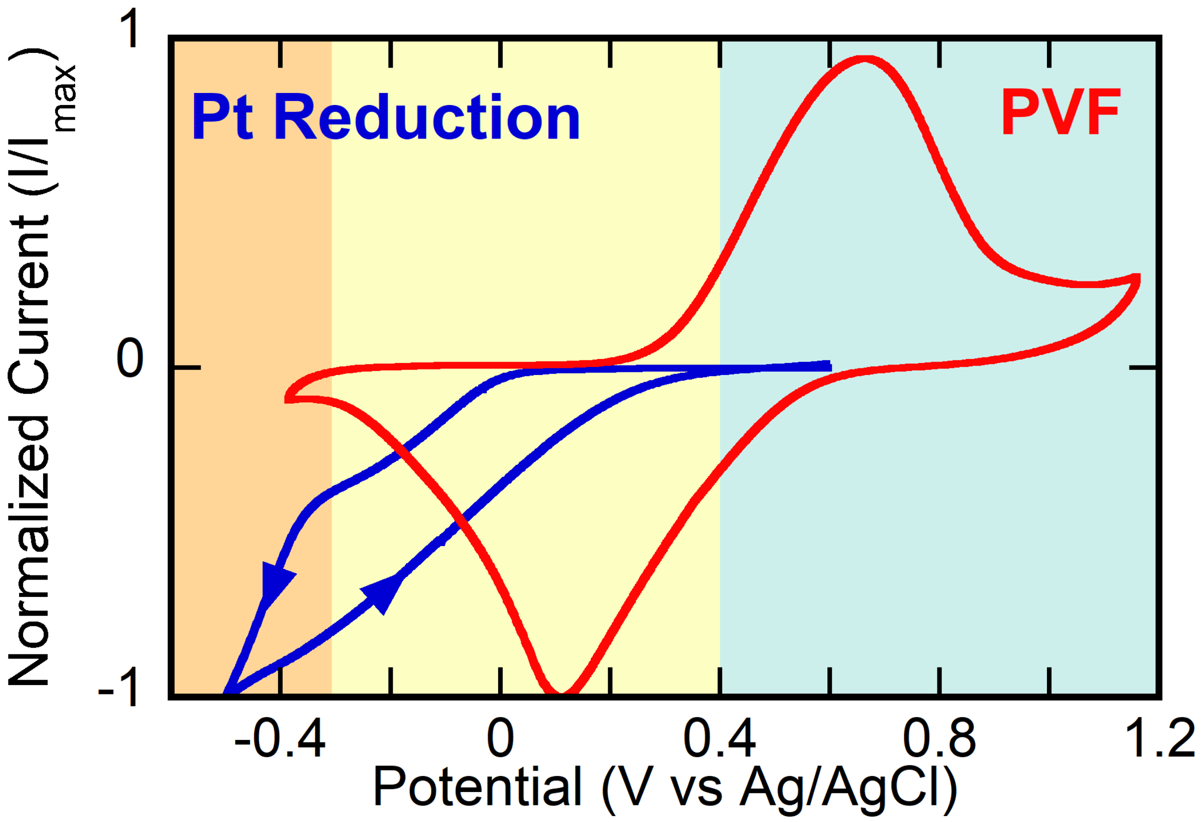

Figure S18. Cyclic voltammogram of PVF-CNT electrode in ethanol solution of 20 mM NaClO4 (red) and carbon paper electrode in 5mM chloroplatinic acid in ethanol (blue).

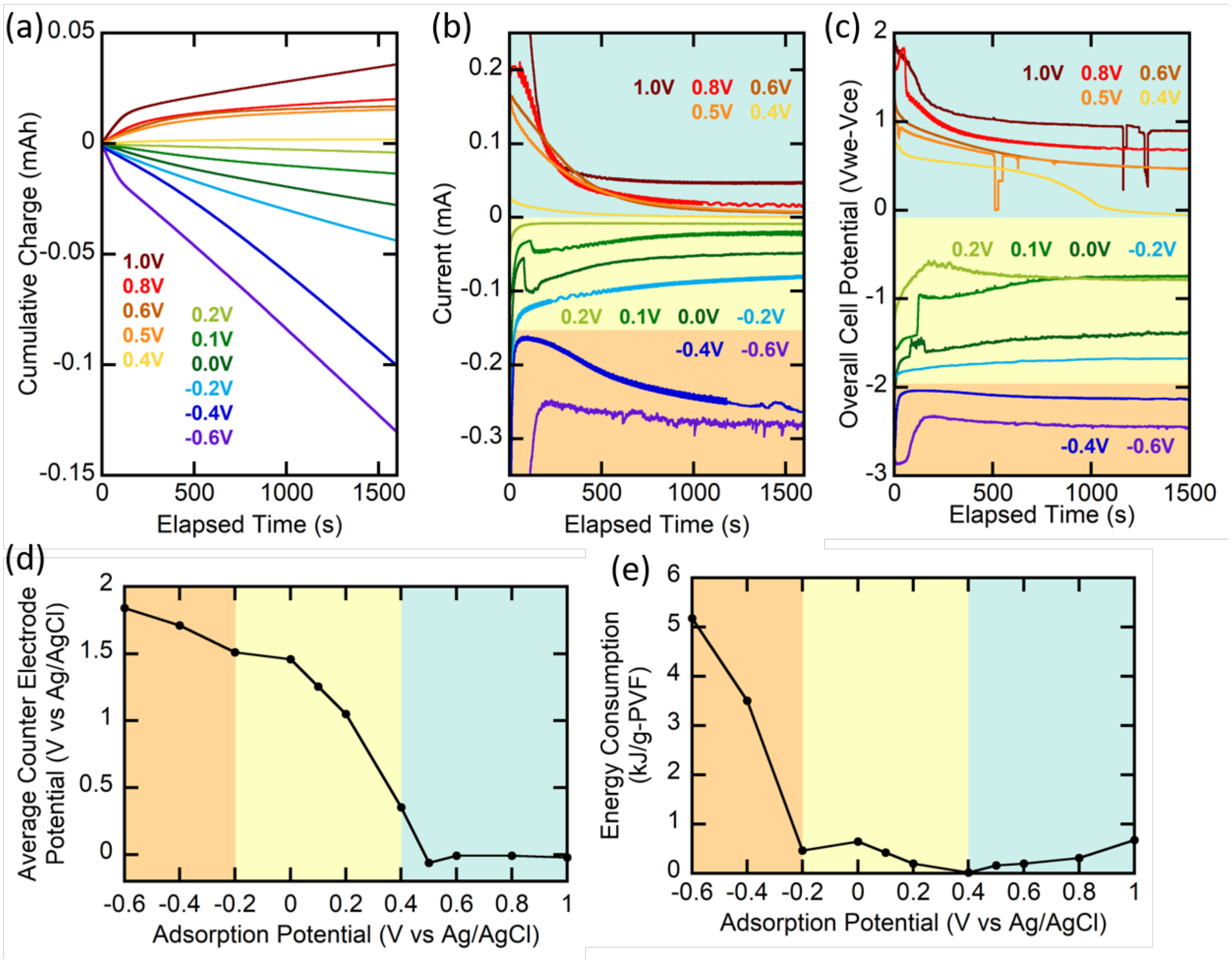

Figure S19. Chronoamperometric data from 1 mM Speier's catalyst adsorption experiments in ethanol over a range of applied potentials. (a) Cumulative charge, (b) current, and (c) overall cell potential vs time for each applied potential. The (d) average counter electrode potential and (e) energy consumption versus applied potential is shown.

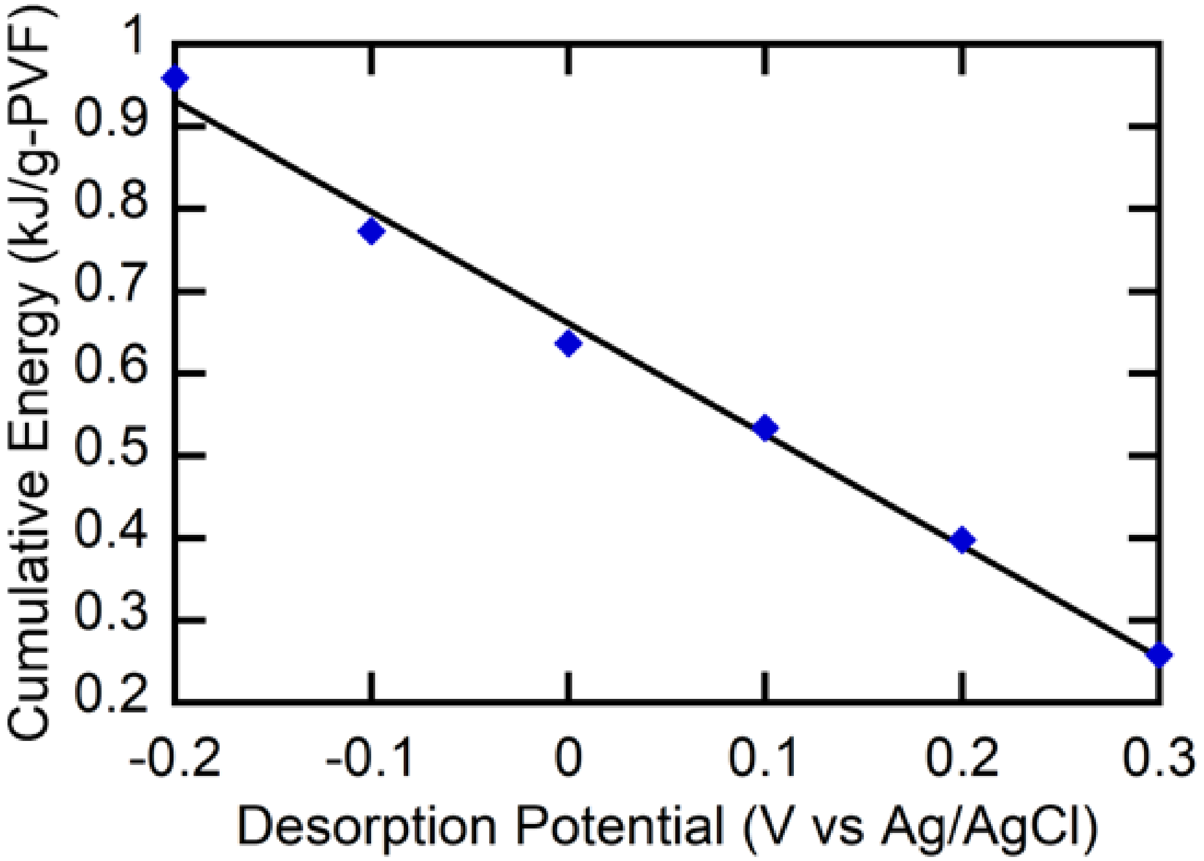

Figure S20. The energy consumed during Speier's catalyst desorption normalized by PVF mass at various desorption potentials.

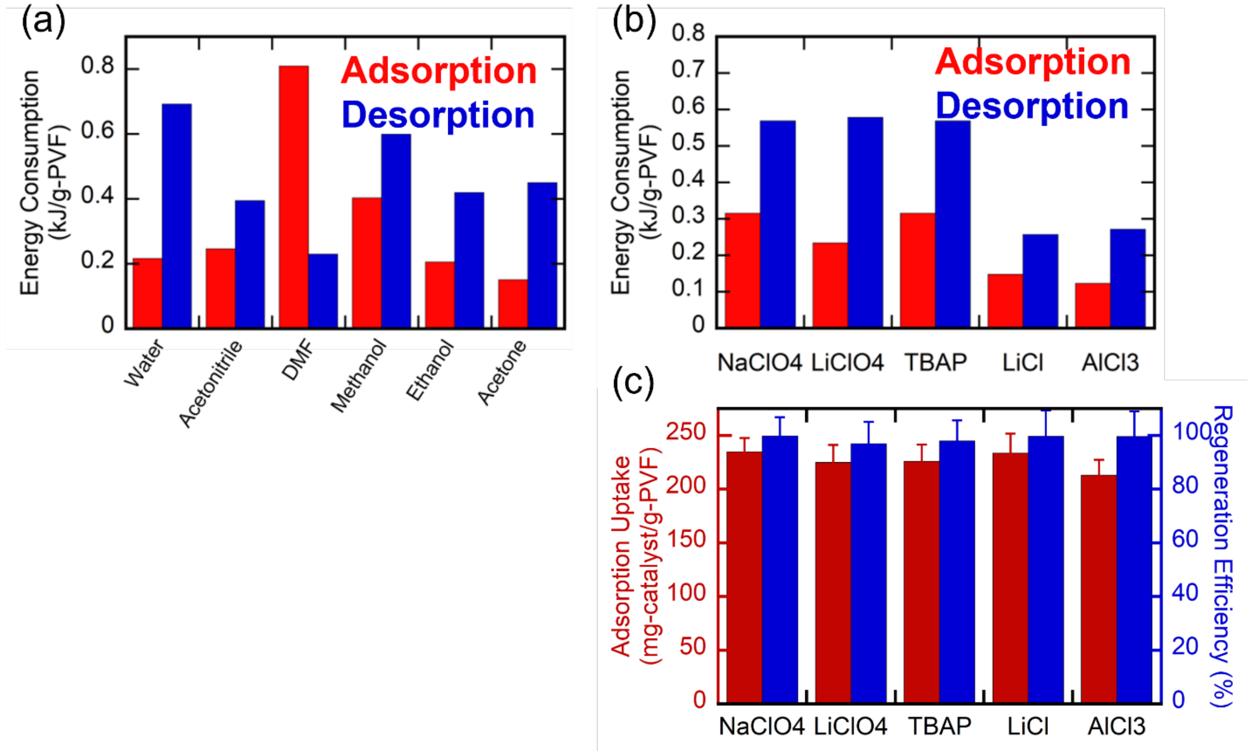

Figure S21. Energy consumption during adsorption (red) and desorption (blue) of Speier's catalyst for various (a) solvents and (b) electrolytes, as well as (c) adsorption and regeneration efficiencies under various different electrolytes.

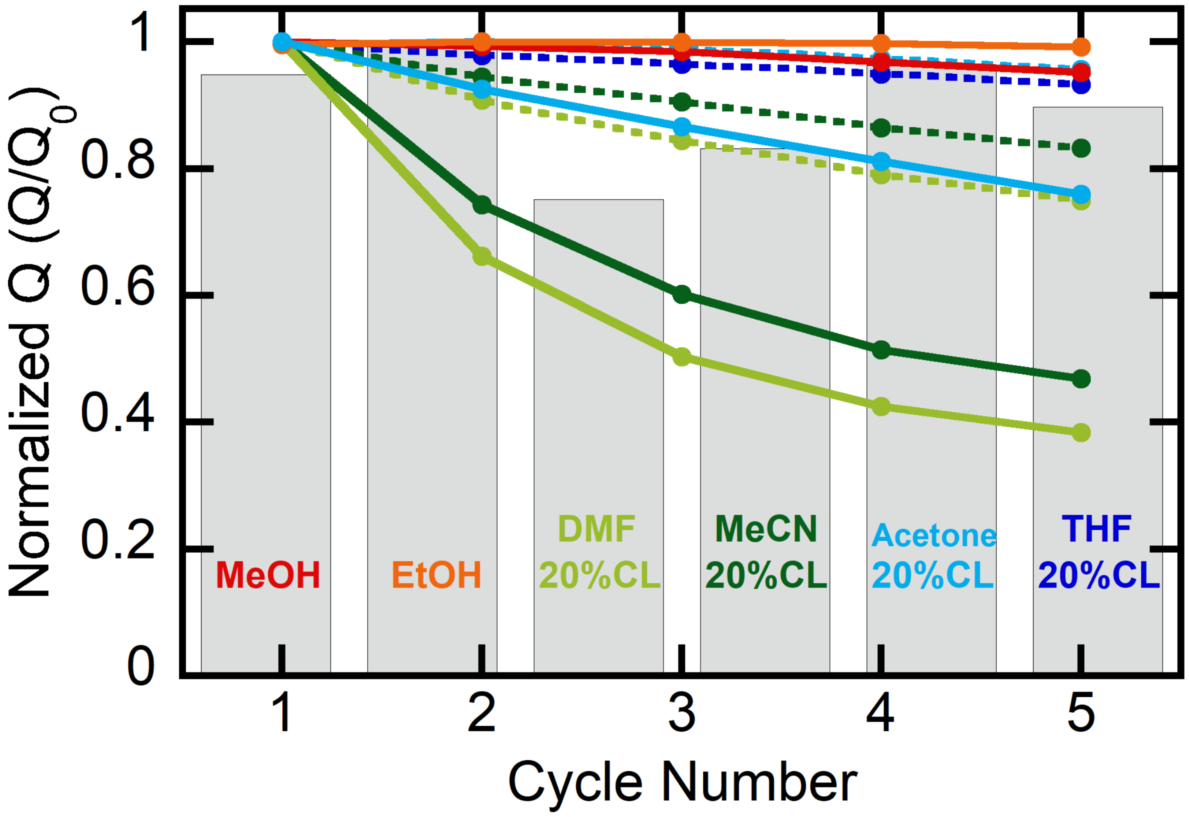

Figure S22. Normalized accumulative charge Q (Q/Q_0) over cyclic voltammetry cycles. The very first cycles were disregarded. Solid lines are with 0 w% crosslinker and dotted lines with 20 w% crosslinker (20%CL). The grey bars represent the normalized accumulative charge at the fifth cycle.

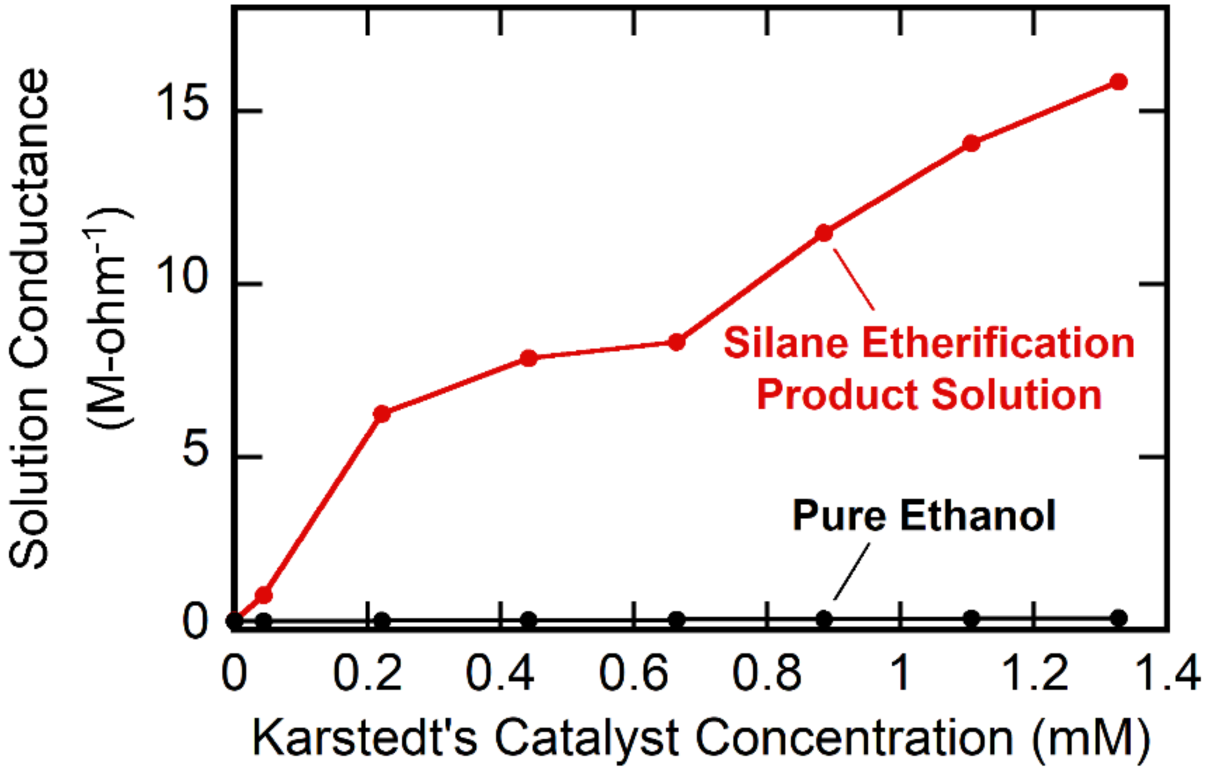

Figure S23. Solution conductance of two different solutions as Karstedt's catalyst is added and left to equilibrate. Ethanol (in black) shows very little increase in solution conductivity as catalyst is added. Silane etherification product solution (in red) initially containing 2:1 ethanol and triethylsilane shows a sharp linear increase in solution conductivity as Karstedt's catalyst is added and allowed to equilibrate. Conductivity measurements were taken with two carbon paper electrodes in parallel 1cm apart with each electrode having an exposed area of 1cm by 1cm.

9. UV-VIS Data

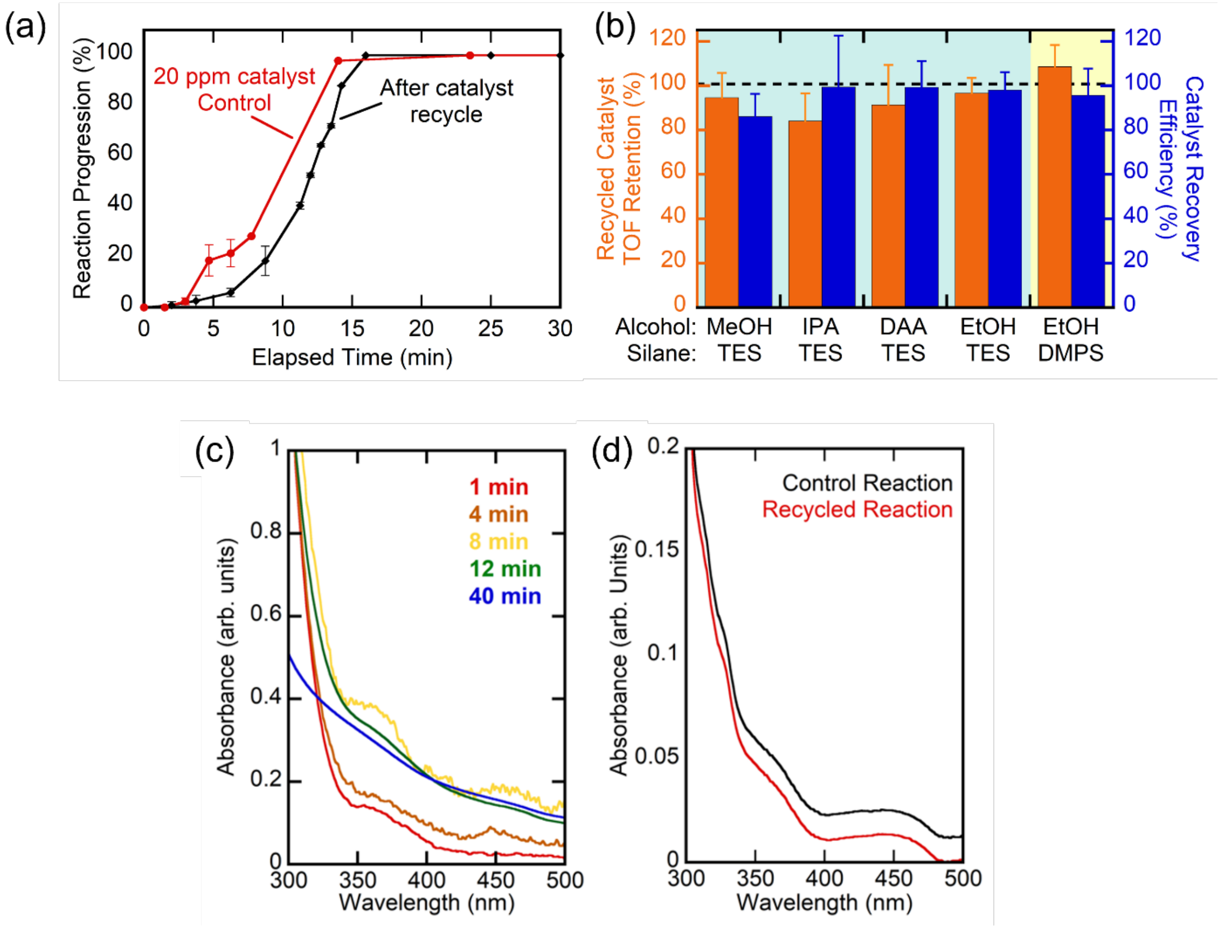

Figure S24. Electrochemical catalyst recovery. a) Comparison of the reaction kinetics of silane etherification (using EtOH and TES) with electrochemically recycled Speier's catalyst to a control using as-received catalyst. b) Catalyst recovery performance from silane etherification reactions using various reactant substrates. A series of (alcohols methanol (MeOH), isopropanol (IPA), diacetone alcohol (DAA), and ethanol (EtOH)) were used along with different silanes (triethylsilane (TES) and dimethylphenylsilane (DMPS)). c) UV-VIS spectroscopy spectra of triethylsilane etherification reaction with ethanol catalyzed with 30 ppm Speier's catalyst. At the 8-minute line (yellow) the reaction has ended. The noise observed at 1,4, and 8 minutes is due to hydrogen bubble formation. UV-VIS spectra is featureless except for a peak at 360 nm and a faint peak at 460 nm. The 360 nm peak has been observed in literature(43) as the active platinum catalyst species for hydrosilylation. The 360 nm peak is most distinctly observed in our work when silane etherification reaction progresses. d) Comparison of UV-VIS spectra directly before adsorption from the control reaction (black) and directly after desorption into fresh reactants (red).

10. Wacker catalyst recycle optimization

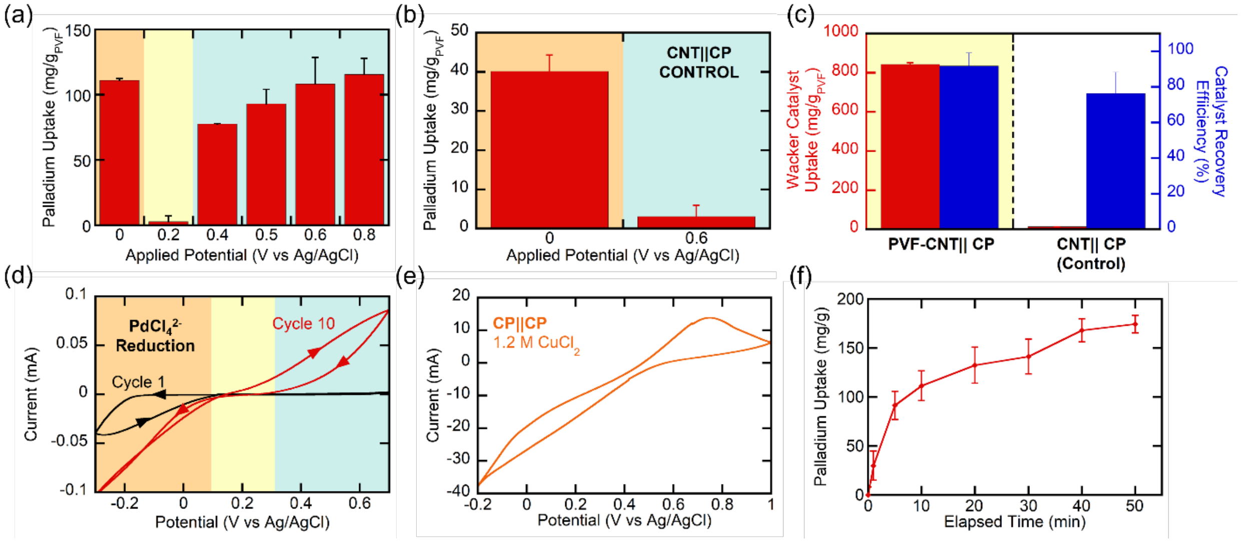

Figure S25. All experiments used an initial Palladium solution contained 1mM $PdCl_2$ and 20 mM $CuCl_2$ in 7:1 methanol/water. a) Palladium uptake over a range of applied potentials with PVF-CNT as the working electrode. b) Palladium uptake without PVF on the working electrode at 0.0V and 0.6V vs Ag/AgCl. c) Uptake and recovery efficiency of in situ wacker catalyst recycle with PVF-CNT (left) and without PVF (right). d) voltammogram of K_2PdCl_4 in methanol showing palladium electrodeposition and stripping. e) voltammogram of $CuCl_2$ showing redox behavior. f) Palladium uptake kinetics with PVF-CNT electrode at 0.6V over the span of 50 minutes.

11. Cross-coupling catalyst recycle optimization

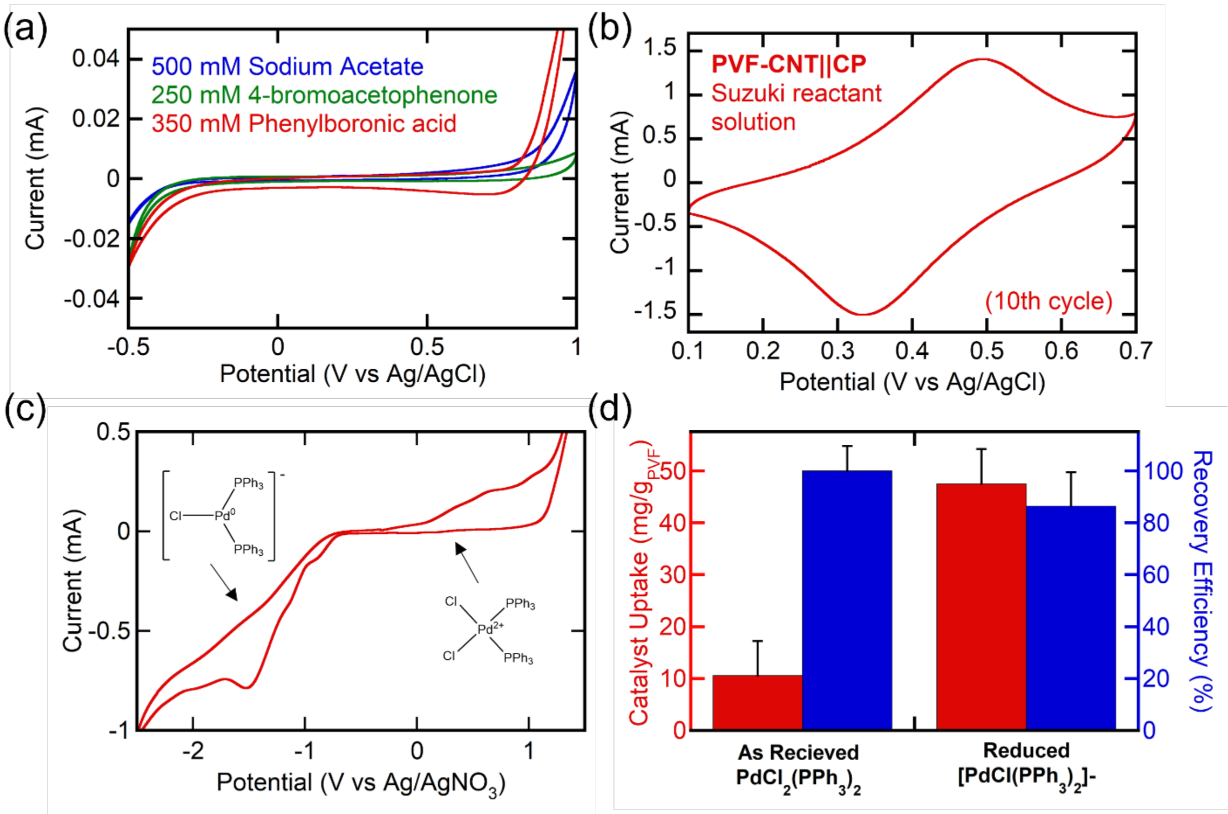

Figure S26. Stability and control results for palladium based recovery. a) cyclic voltammogram electrochemical stability study of each individual Suzuki cross-coupling component. No redox behavior is observed for any component. b) cyclic voltammogram of PVF-CNT electrode in Suzuki coupling reaction solution to verify PVF electrode stability in cross-coupling environment. c) cyclic voltammogram of 2 mM PdCl2(PPh3)2 and 300 mM TBABF4 in THF. Pd reduction to a stable anionic Pd(0) complex is observed at -1.5V vs Ag/AgNO3. d) Comparison of uptake and regeneration efficiency between as received PdCl2(PPh3)2 and after being reduced to an anionic Pd(0) form. Higher uptake is observed when in anionic form.

12. Additional flow cell figures

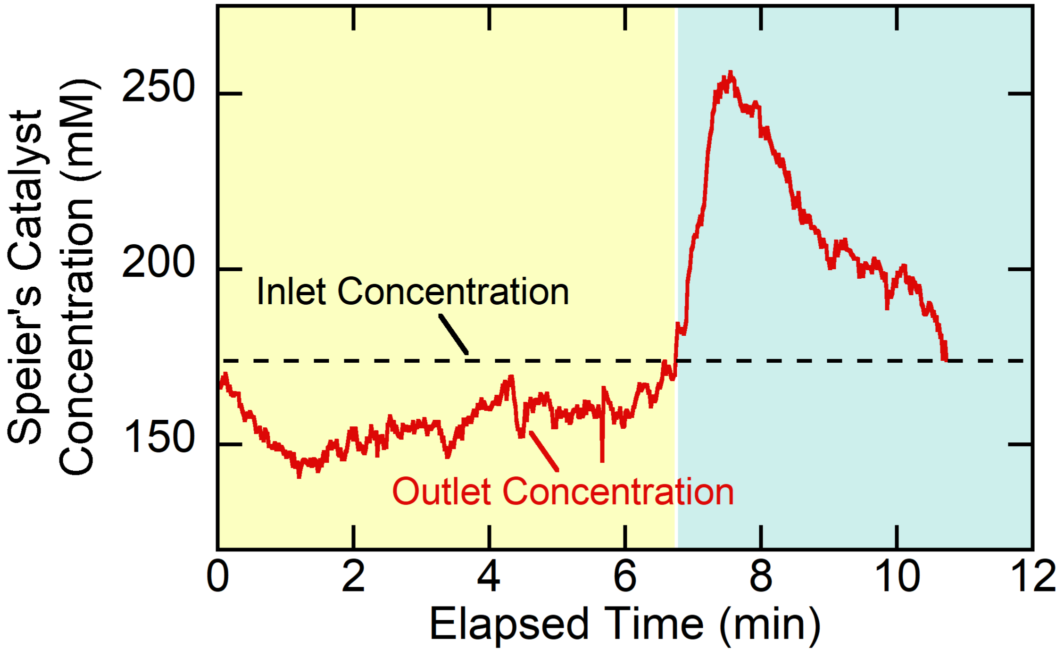

Figure S27. Inlet (black, dashed) and outlet (red, solid) Speier's catalyst concentrations from flow-by cell. From 0 to 6.4 minutes, and oxidizing potential was applied and catalyst was adsorbed – shown by the outlet concentration being lower than the inlet concentration. From 6.4 minutes to 10.4 minutes, a reducing potential was applied and catalyst was released – showen by the outlet concentration being higher than the inlet concentration.

13. ¹H-NMR Spectra

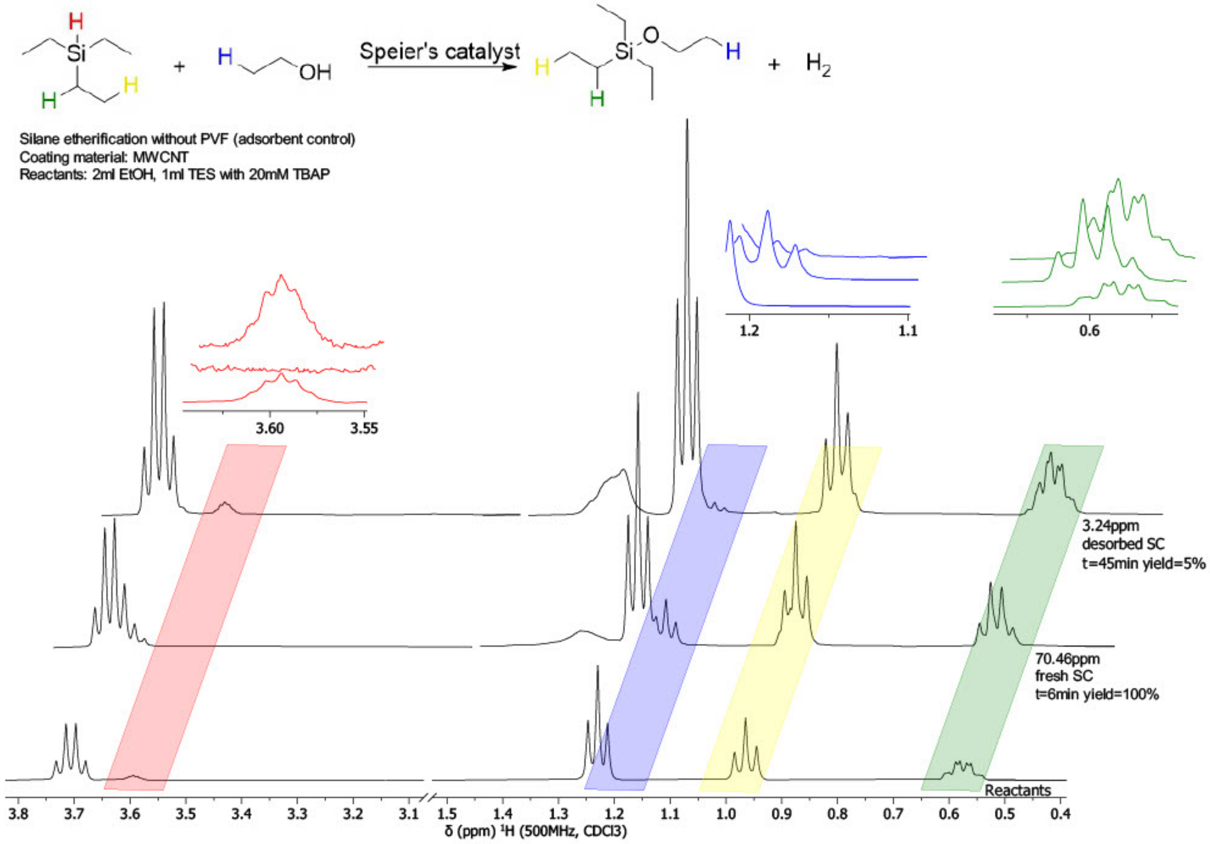

Figure S28. A control catalyst recycling experiment where no PVF-CNT coating was used on electrodes. Subsequently, negligible reaction progress is observed.

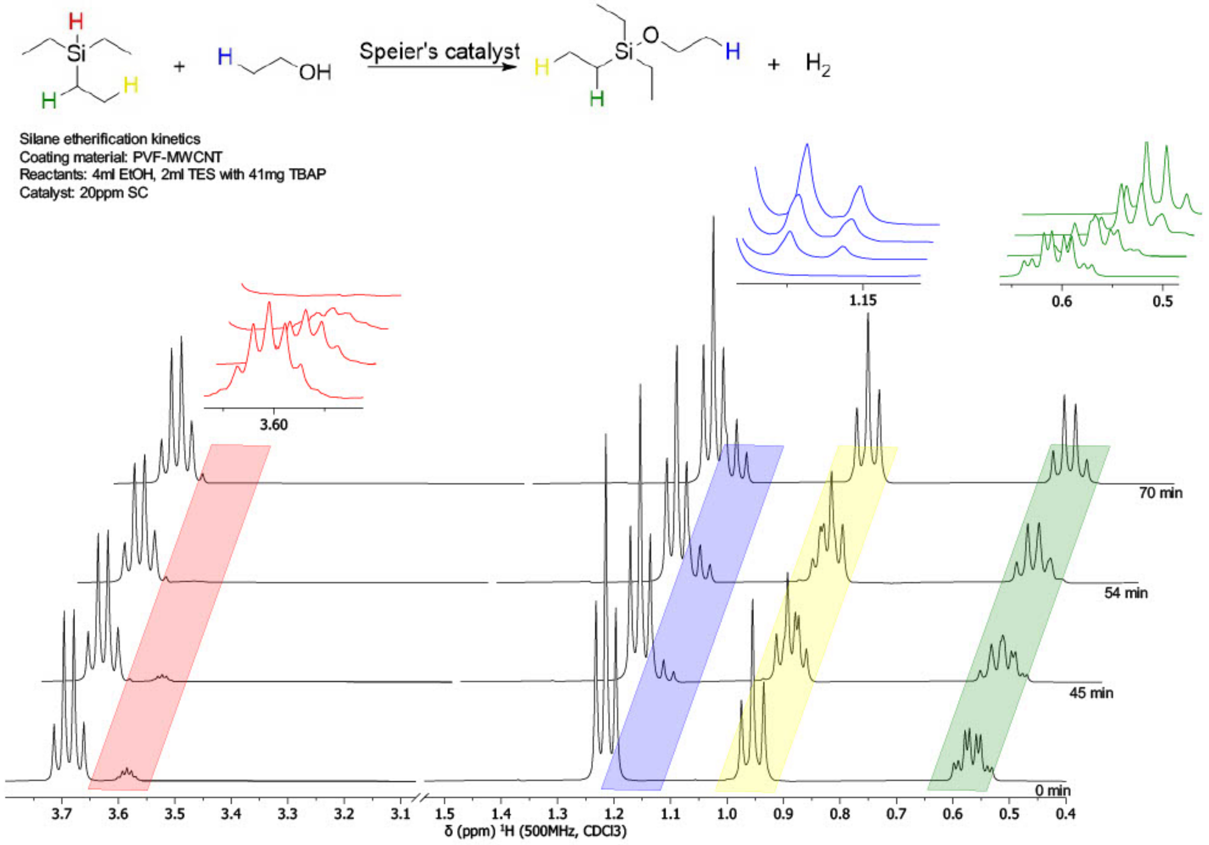

Figure S29. Silane etherification kinetics with Speier's catalyst.

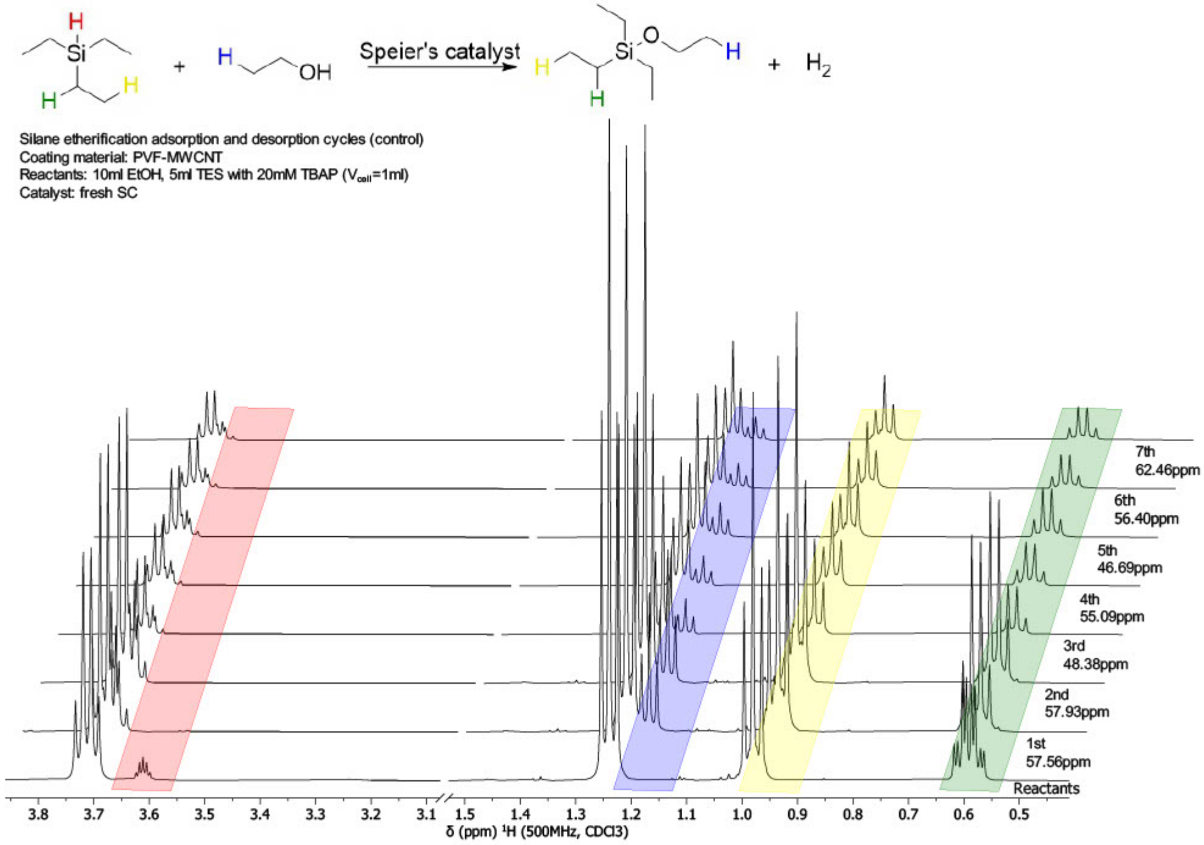

Figure S30. Electrode cycling experiment control reactions.

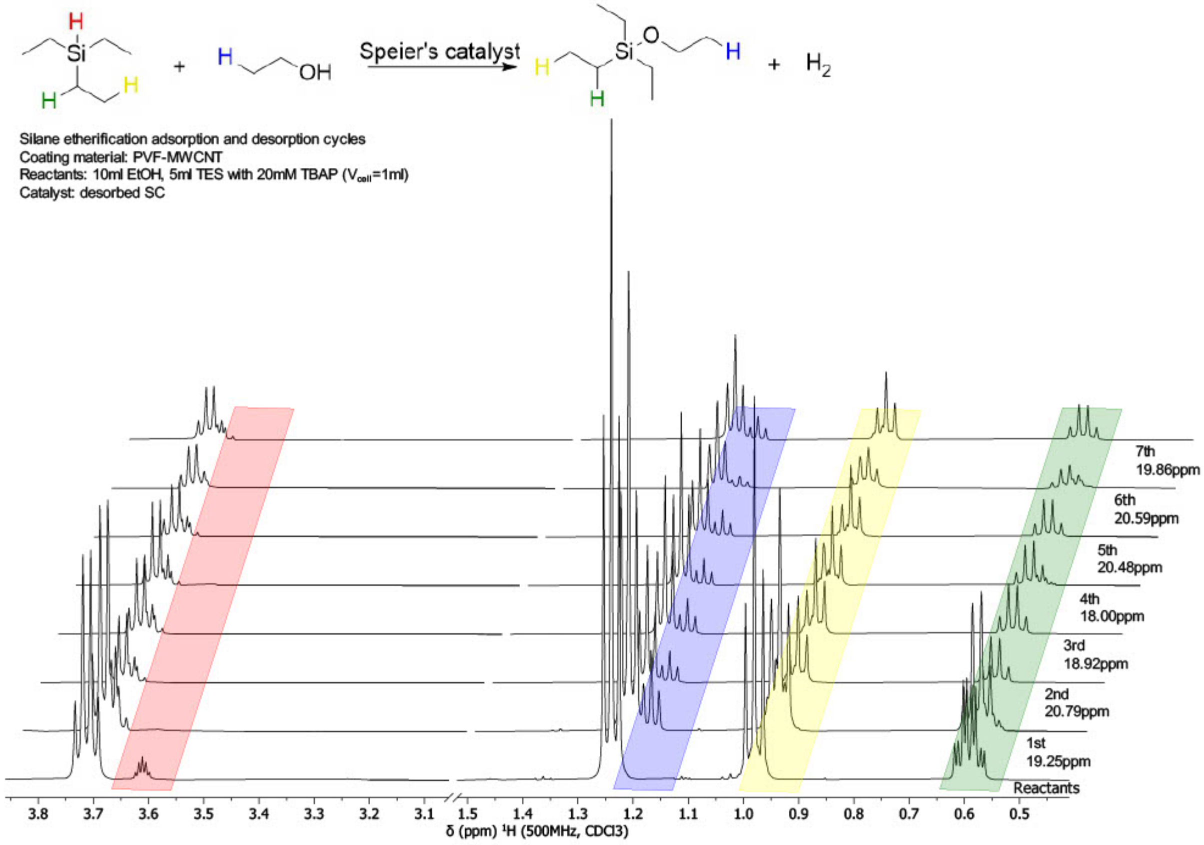

Figure S31. Electrode cycling experiment cycled reactions.

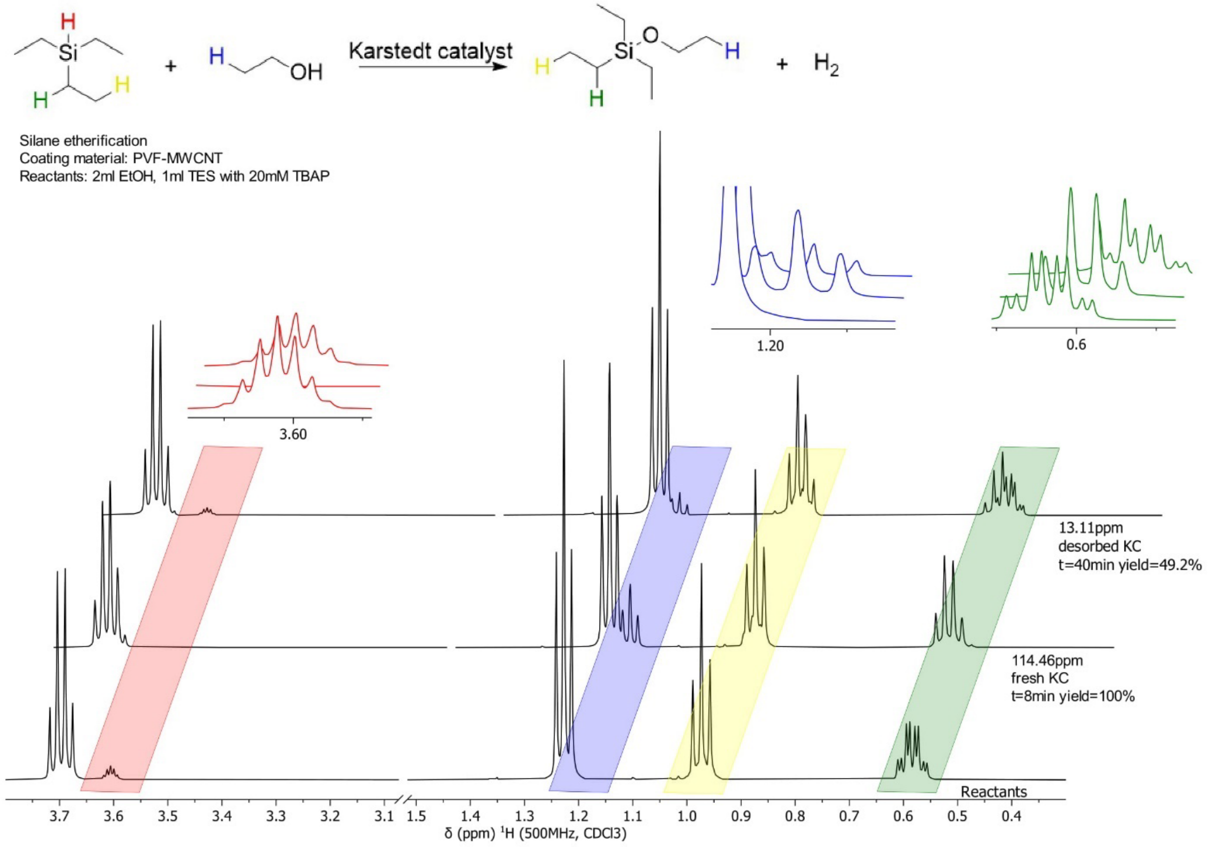

Figure S32. Silane etherification recycling with Karstedt's catalyst.

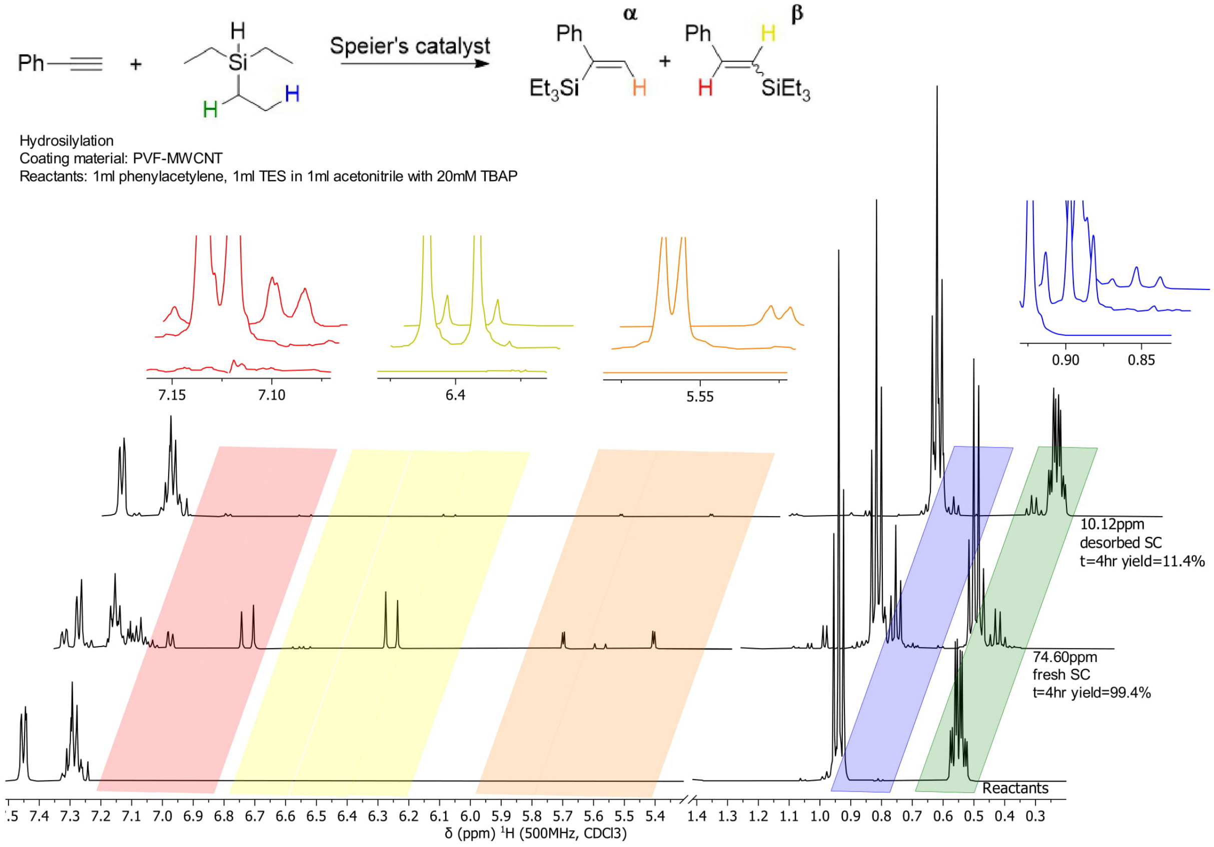

Figure S33. Hydrosilylation reaction cycling with Speier's catalyst.

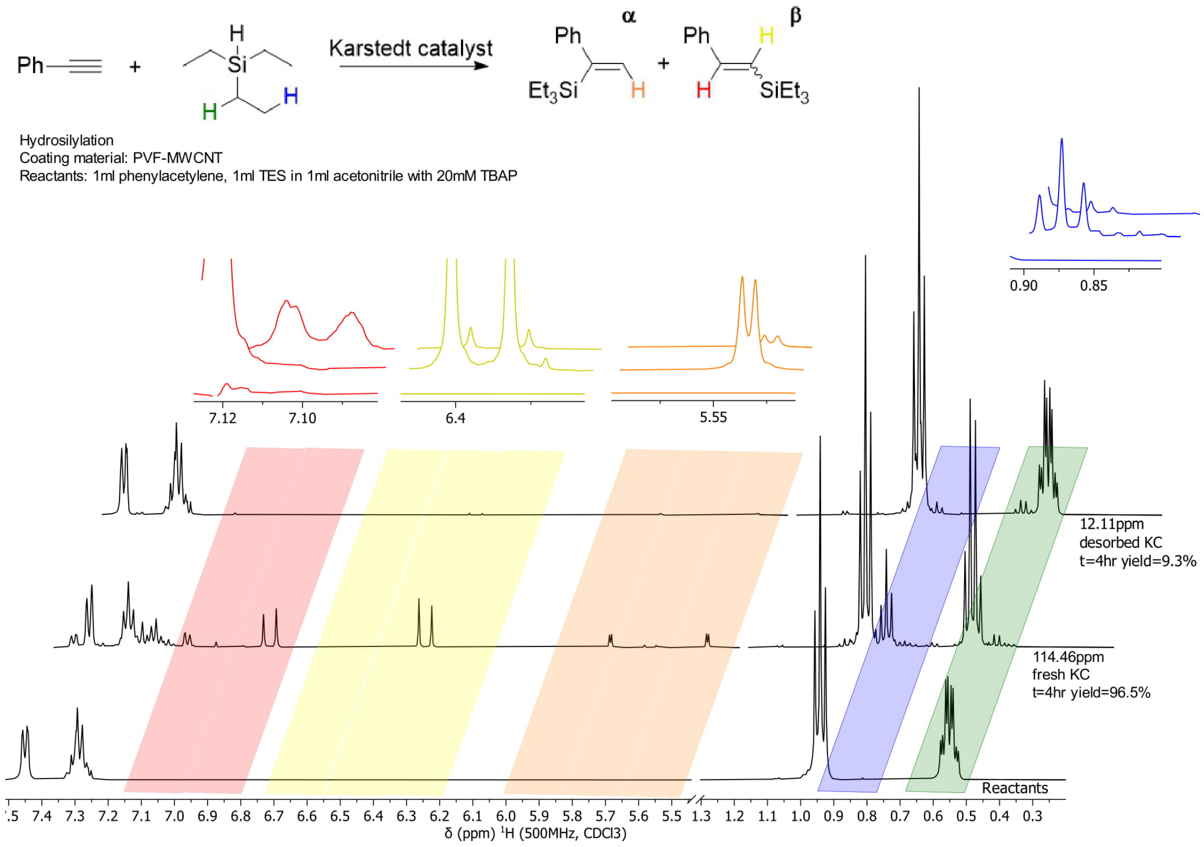

Figure S34. Hydrosilylation reaction cycling with Karstedt's catalyst.

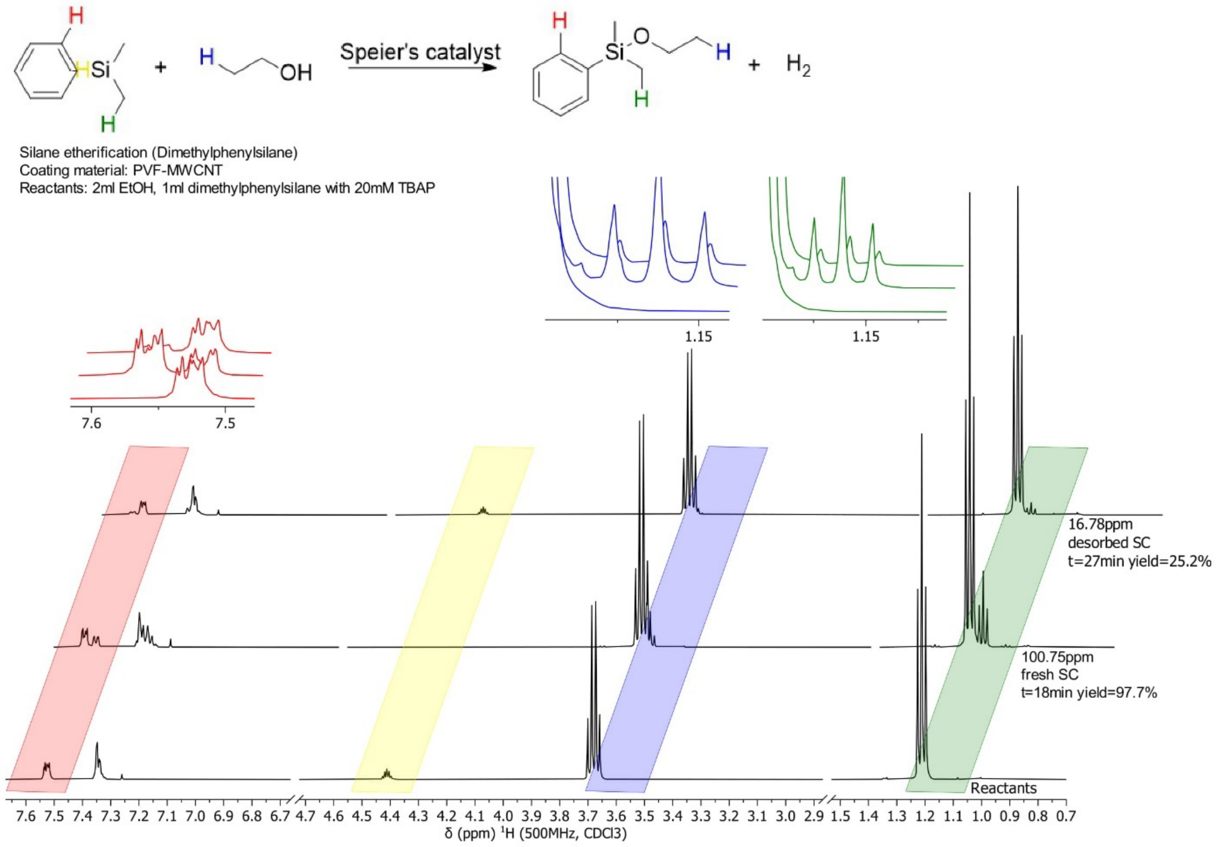

Figure S35. Silane etherification reaction cycling with Speier's catalyst – silane used was dimethylphenylsilane.

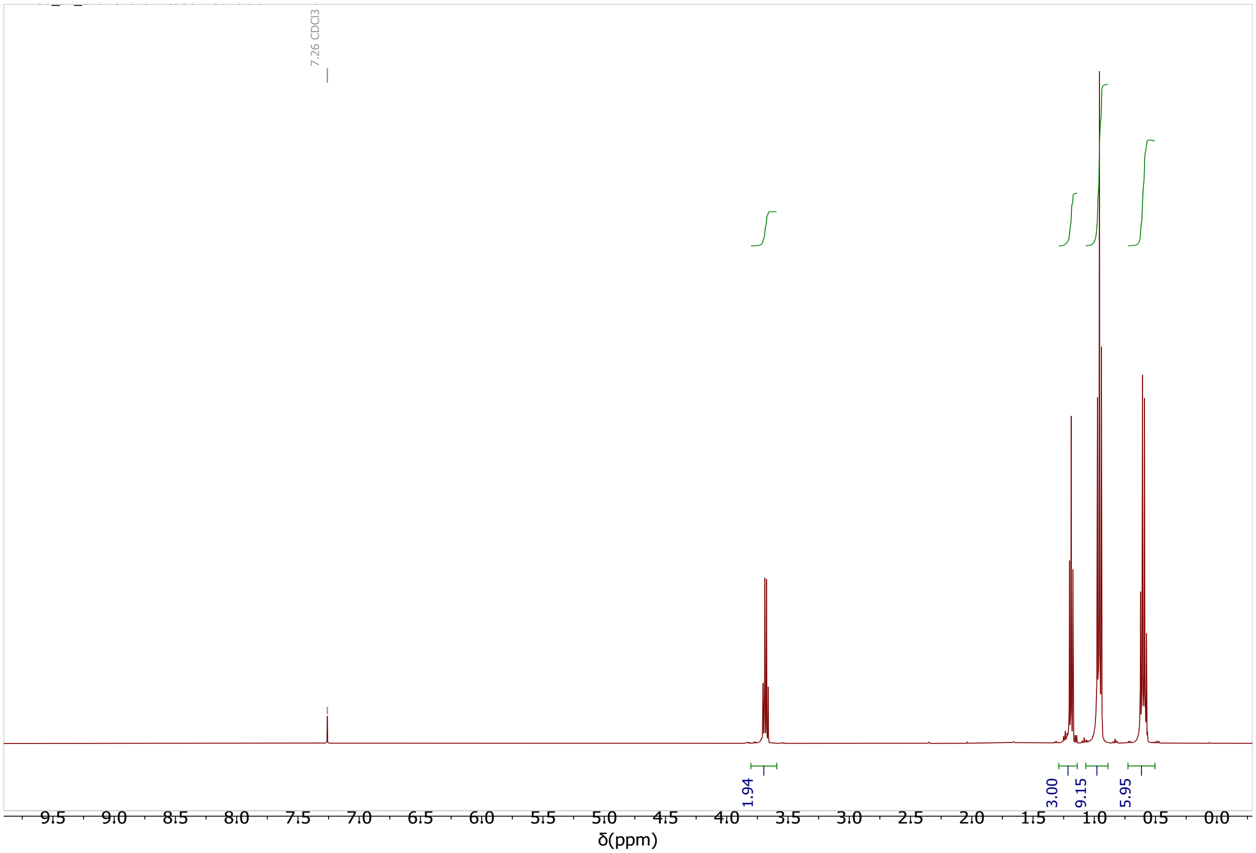

Figure S36. **^{1}H-NMR spectra of isolated triethyl ethoxysilane synthesized using fresh catalyst (500 MHz, $CDCl_3$).**

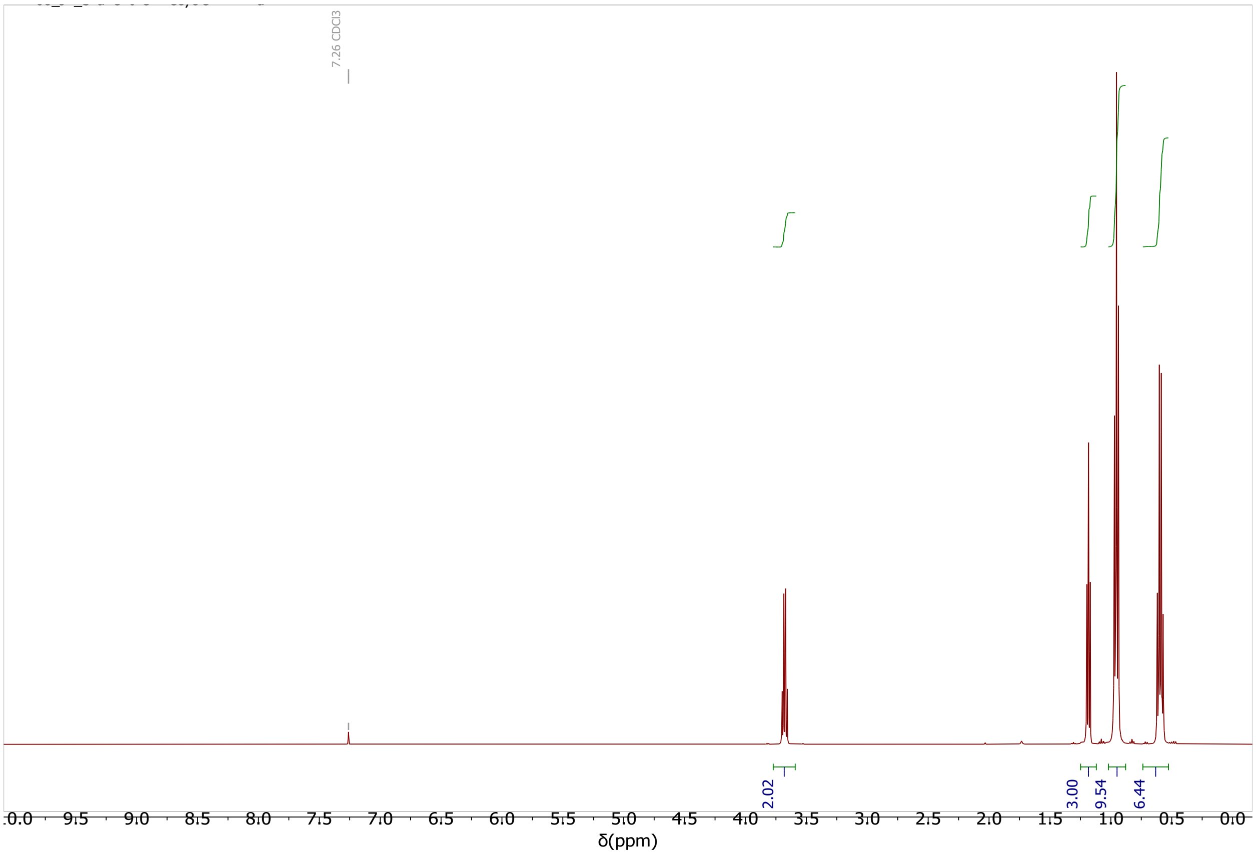

Figure S37. **^{1}H-NMR spectra of isolated triethyl ethoxysilane synthesized using recycled catalyst (500 MHz, $CDCl_3$).**

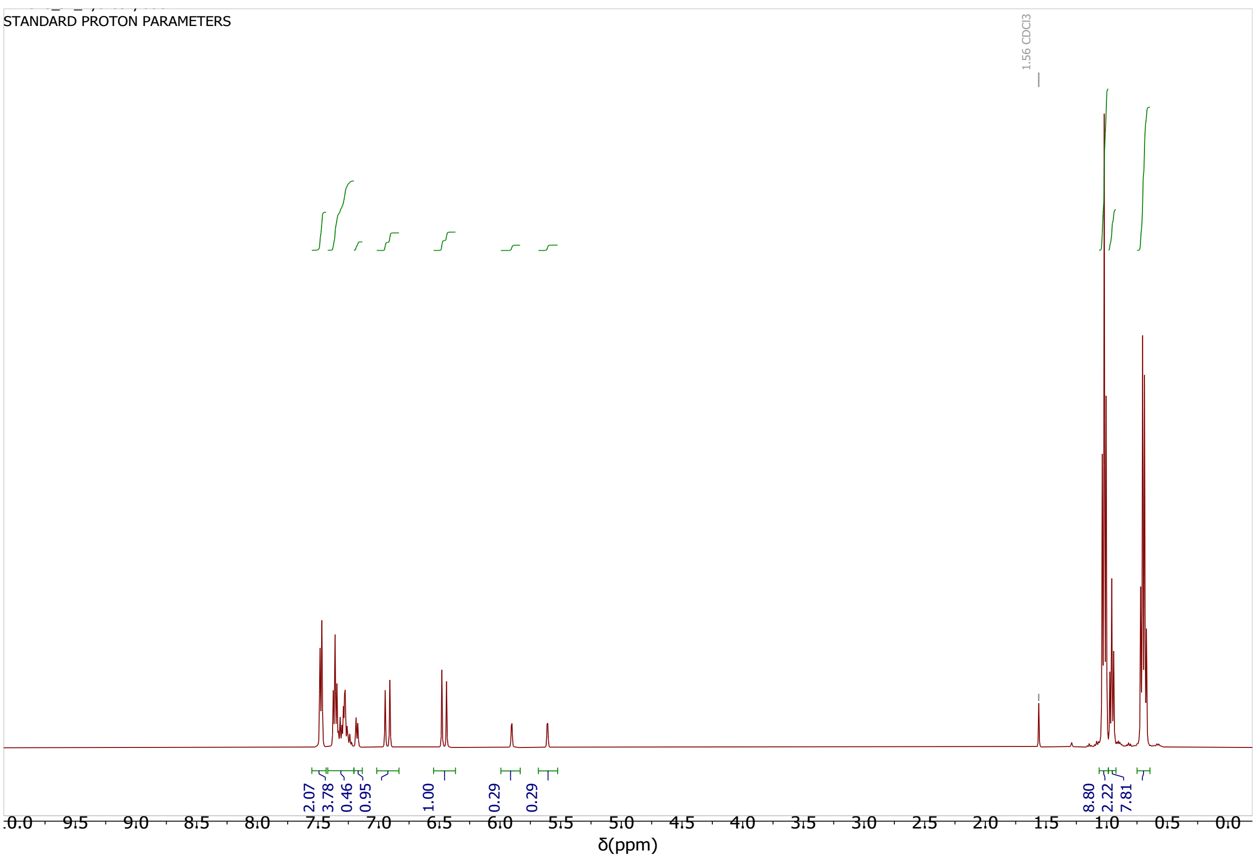

Figure S38. **^{1}H-NMR spectra of the isolated mixture of triethyl(1-phenylvinyl)silane and triethyl(2-phenylvinyl)silane synthesized using fresh catalyst (500 MHz, CD_2Cl_2).**

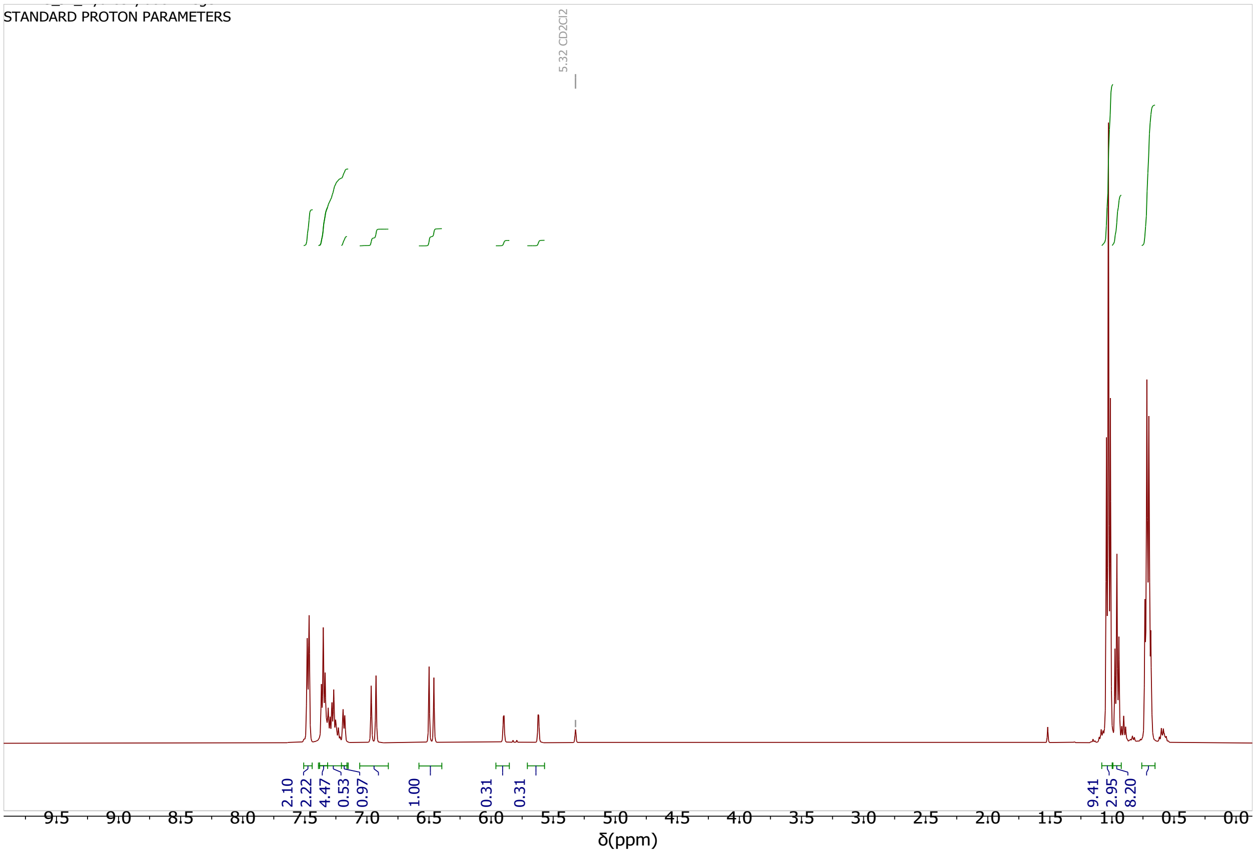

Figure S39. **^{1}H-NMR spectra of the isolated mixture of triethyl(1-phenylvinyl)silane and triethyl(2-phenylvinyl)silane synthesized using recycled catalyst (500 MHz, CD_2Cl_2).**

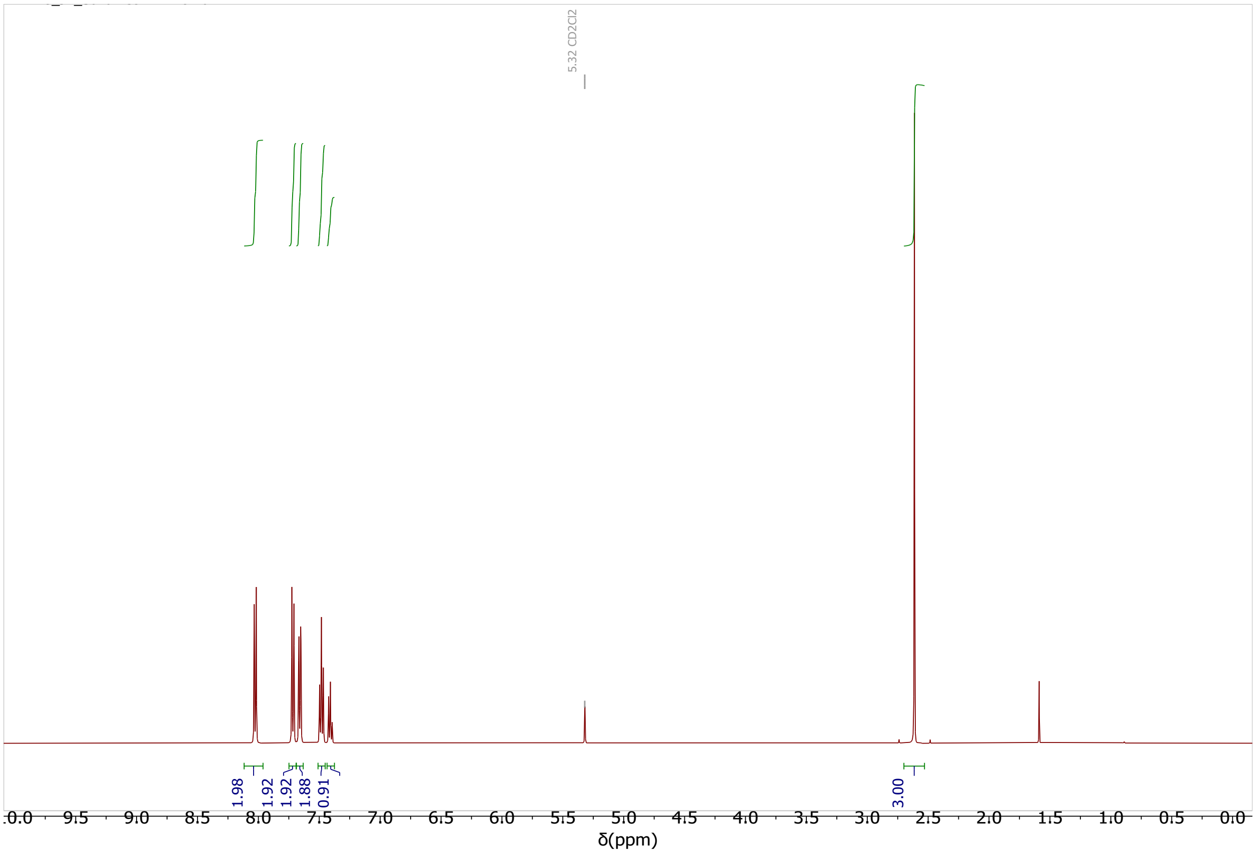

Figure S40. ^{1}H-NMR spectra of isolated 4-acetylbiphenyl synthesized using fresh catalyst (500 MHz, $CDCl_3$).

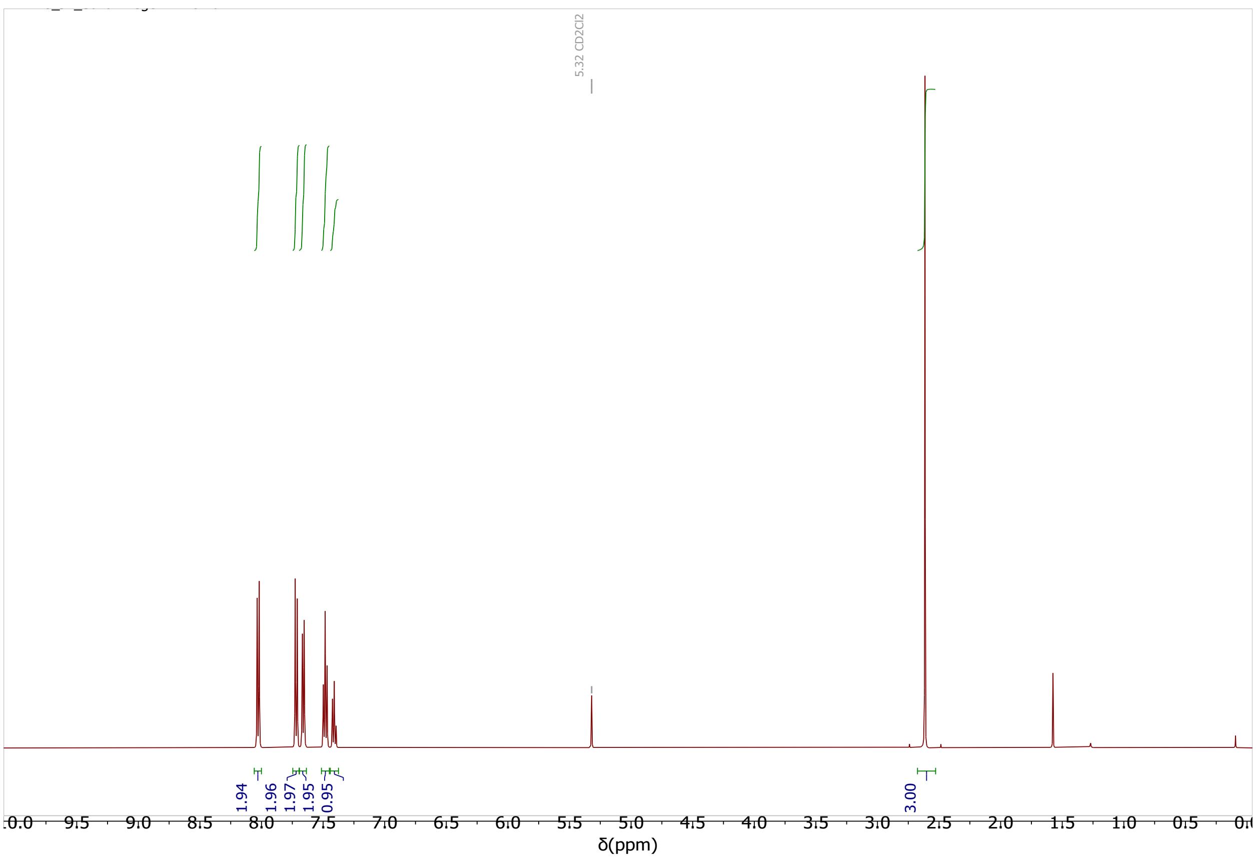

Figure S41. ^{1}H-NMR spectra of isolated 4-acetylbiphenyl synthesized using recycled catalyst (500 MHz, CD_2Cl_2).

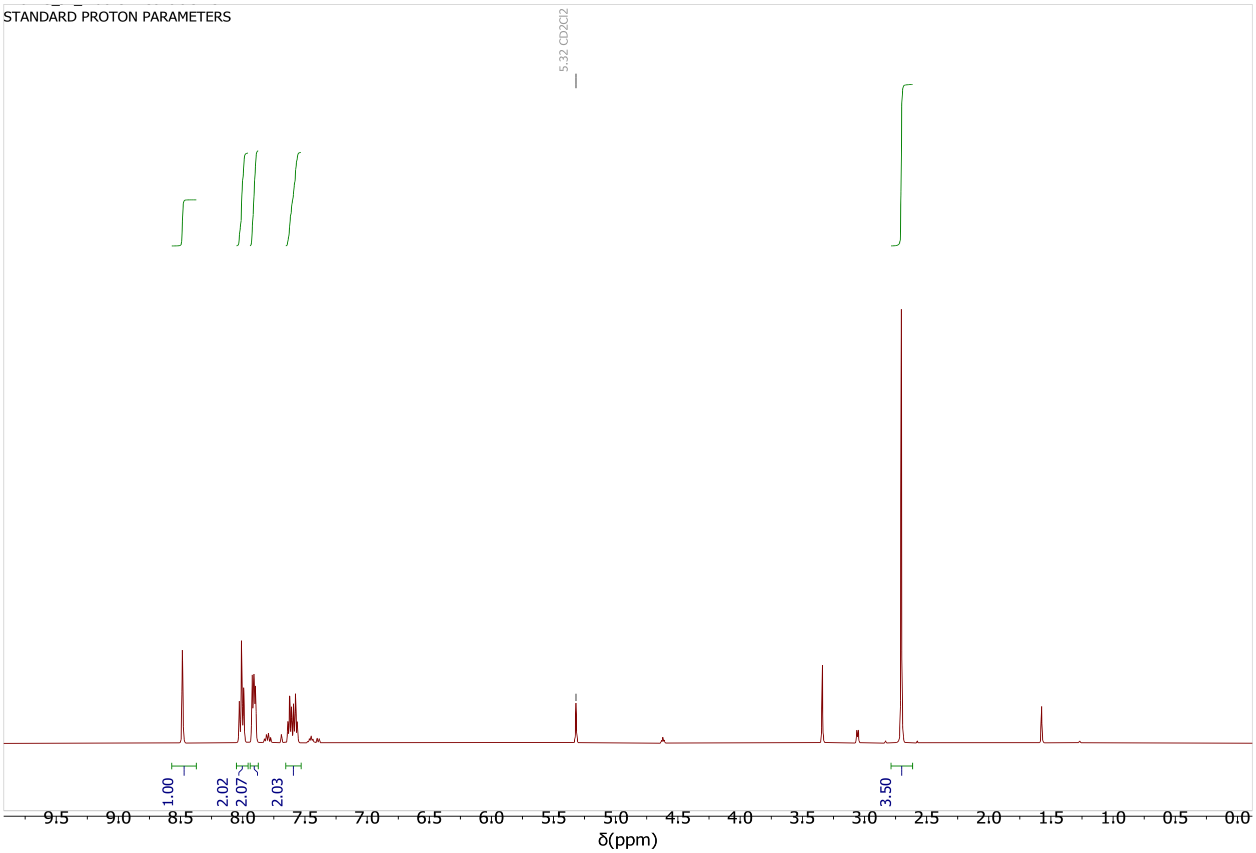

Figure S42. ^{1}H-NMR spectra of isolated 2-acetonaphtone synthesized using fresh catalyst (500 MHz, CD_2Cl_2).

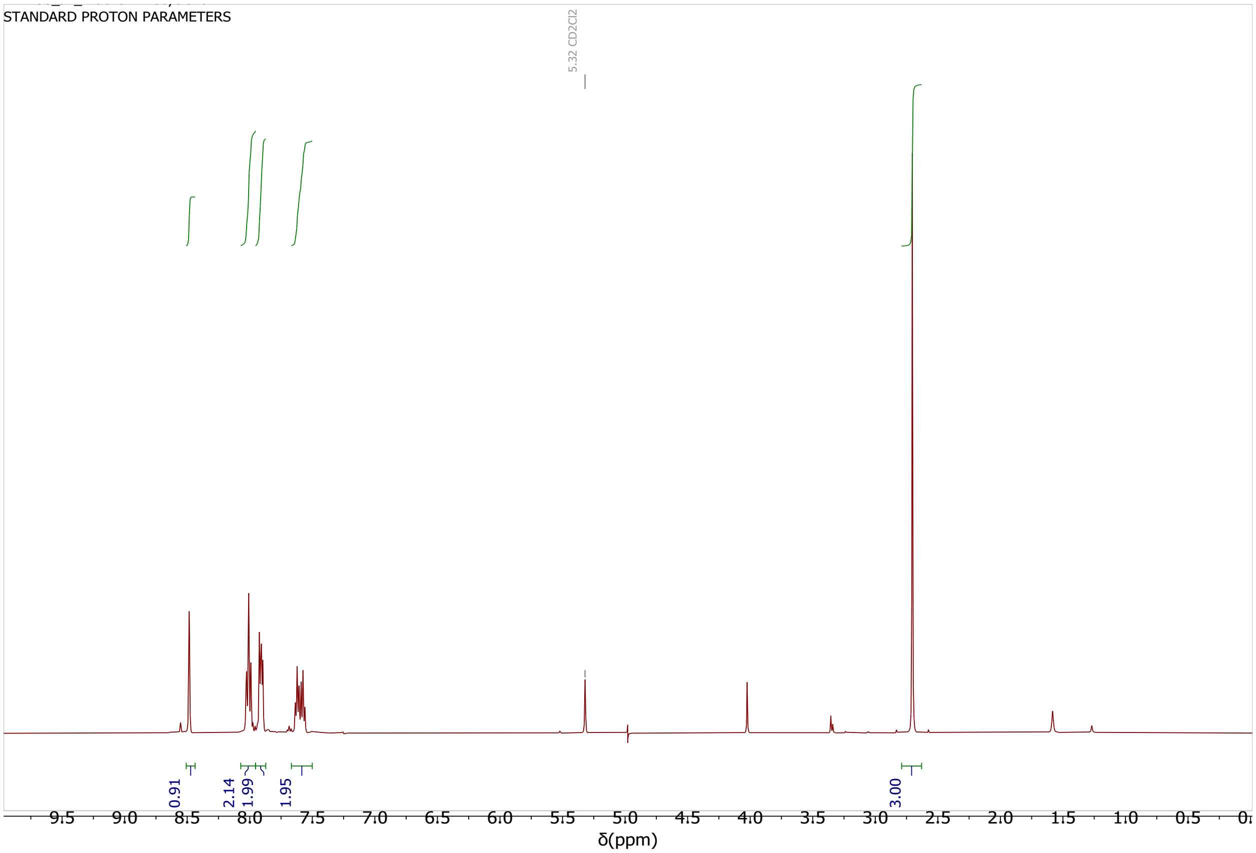

Figure S43. **^{1}H-NMR spectra of isolated 2-acetonaphtone synthesized using recycled catalyst (500 MHz, CD_2Cl_2).**

14. HR-ESI-MS data

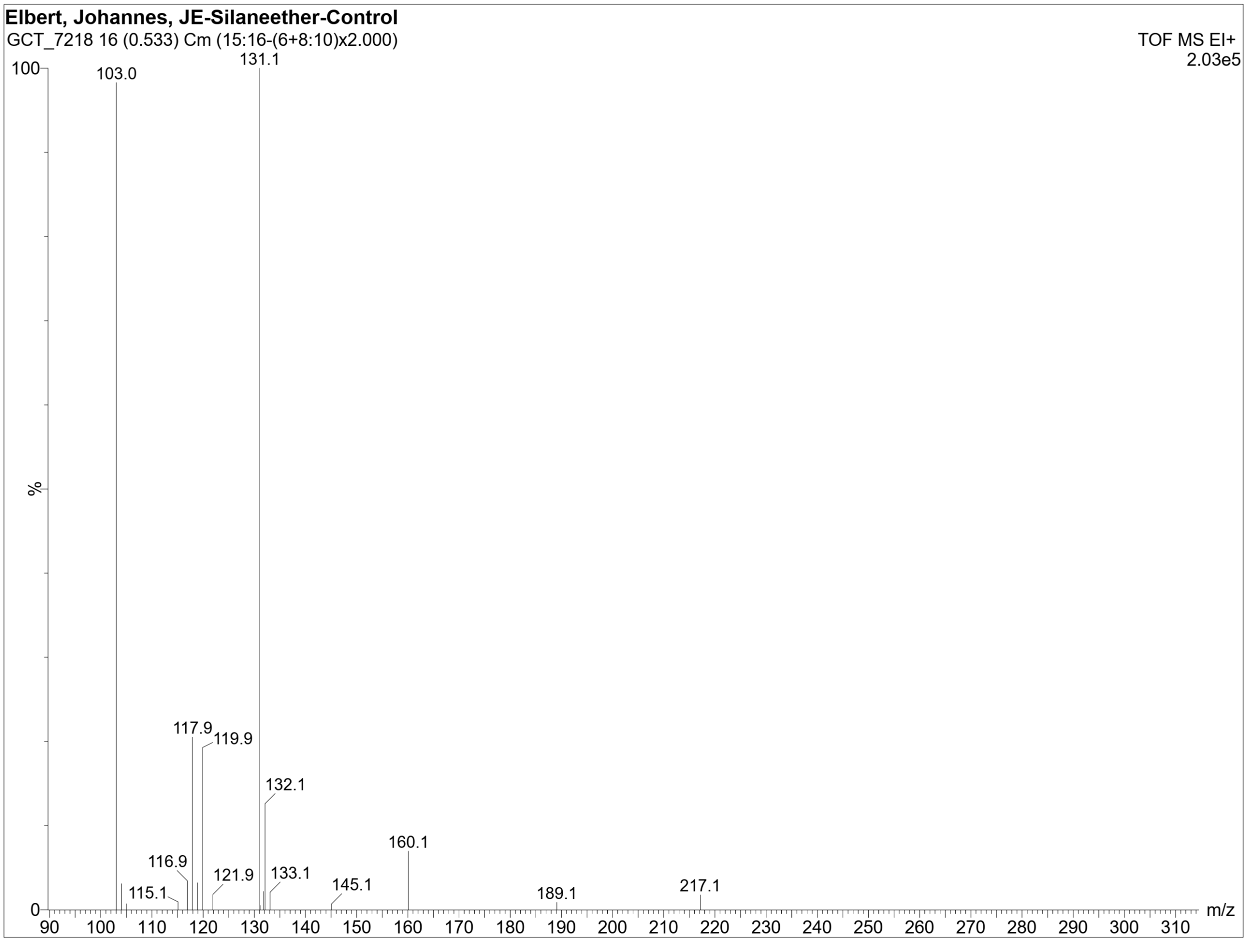

Figure S44. HR-ESI-MS of isolated triethyl ethoxy silane synthesized using fresh catalyst.

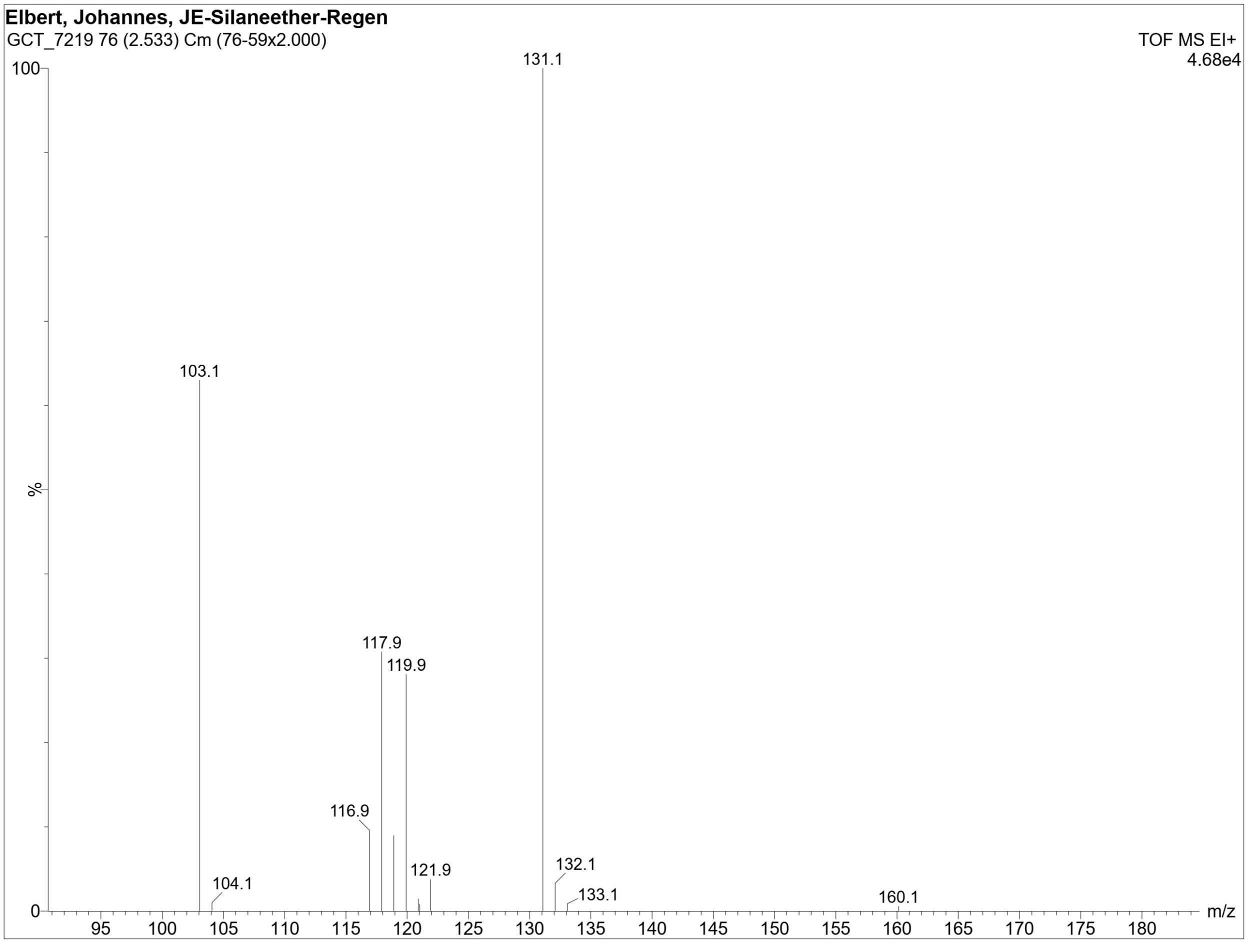

Figure S45. HR-ESI-MS of isolated triethyl ethoxysilane synthesized using recycled catalyst.

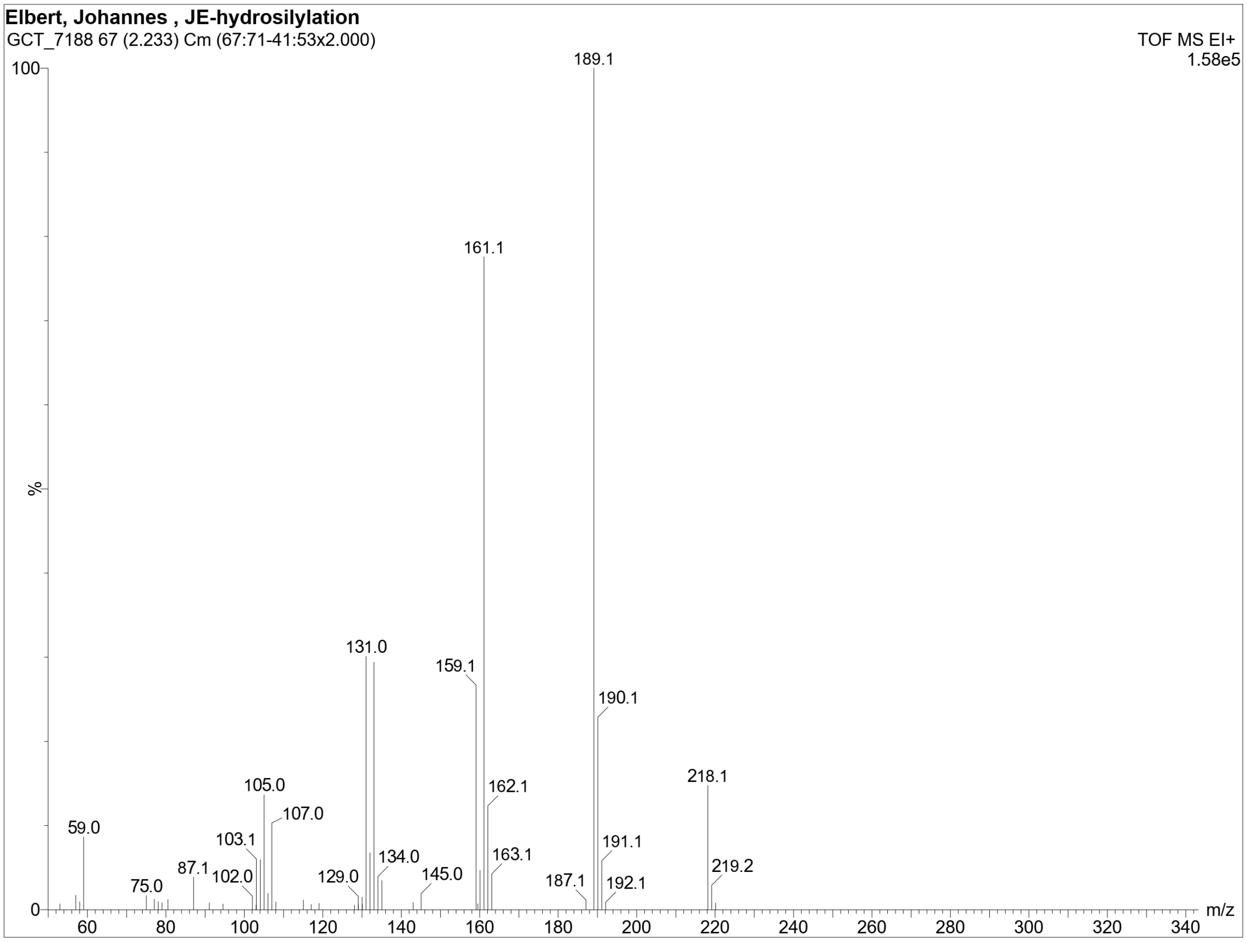

Figure S46. HR-ESI-MS of isolated triethyl(phenyl vinyl)silane synthesized using fresh catalyst.

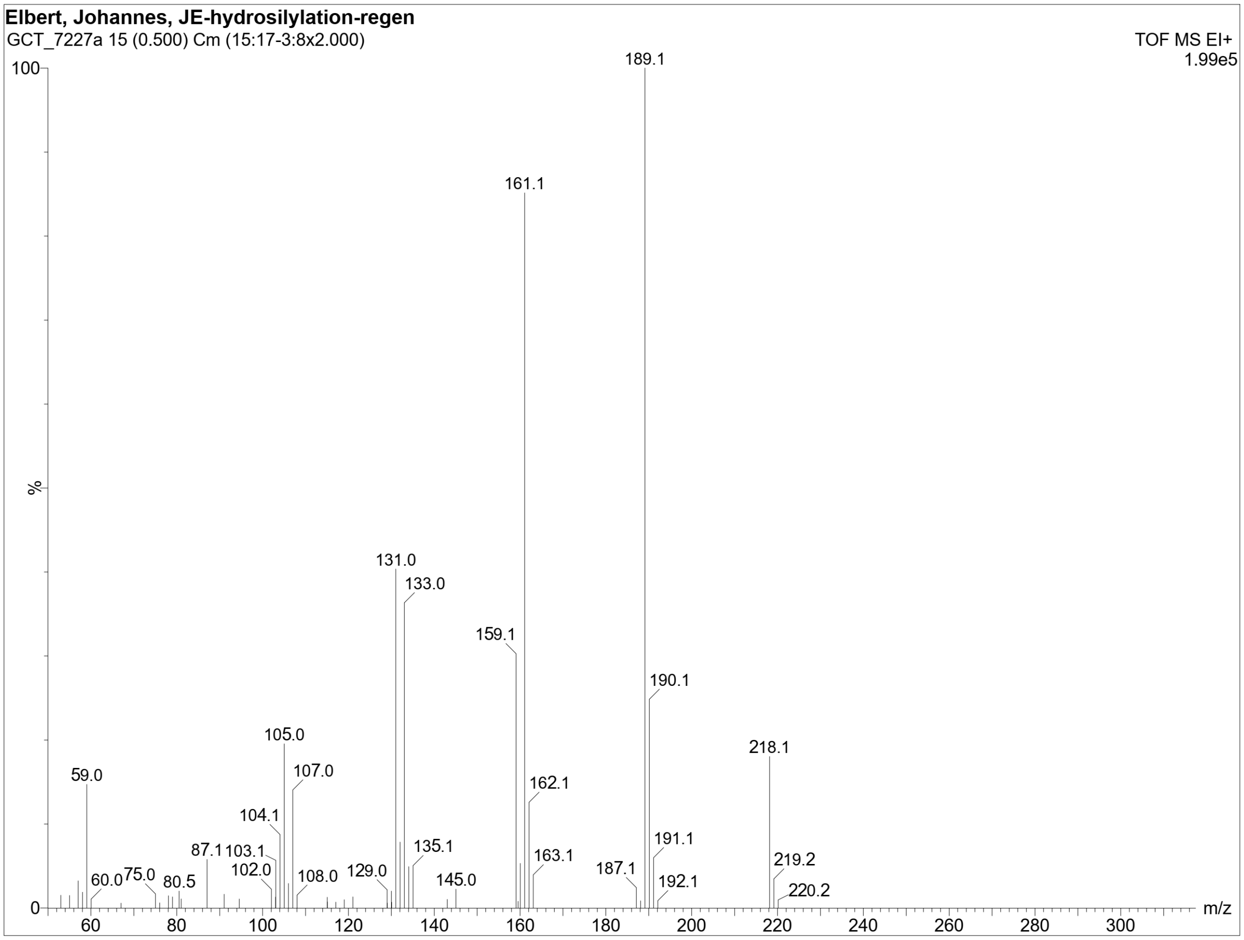

Figure S47. HR-ESI-MS of isolated triethyl(phenyl vinyl)silane synthesized using recycled catalyst.

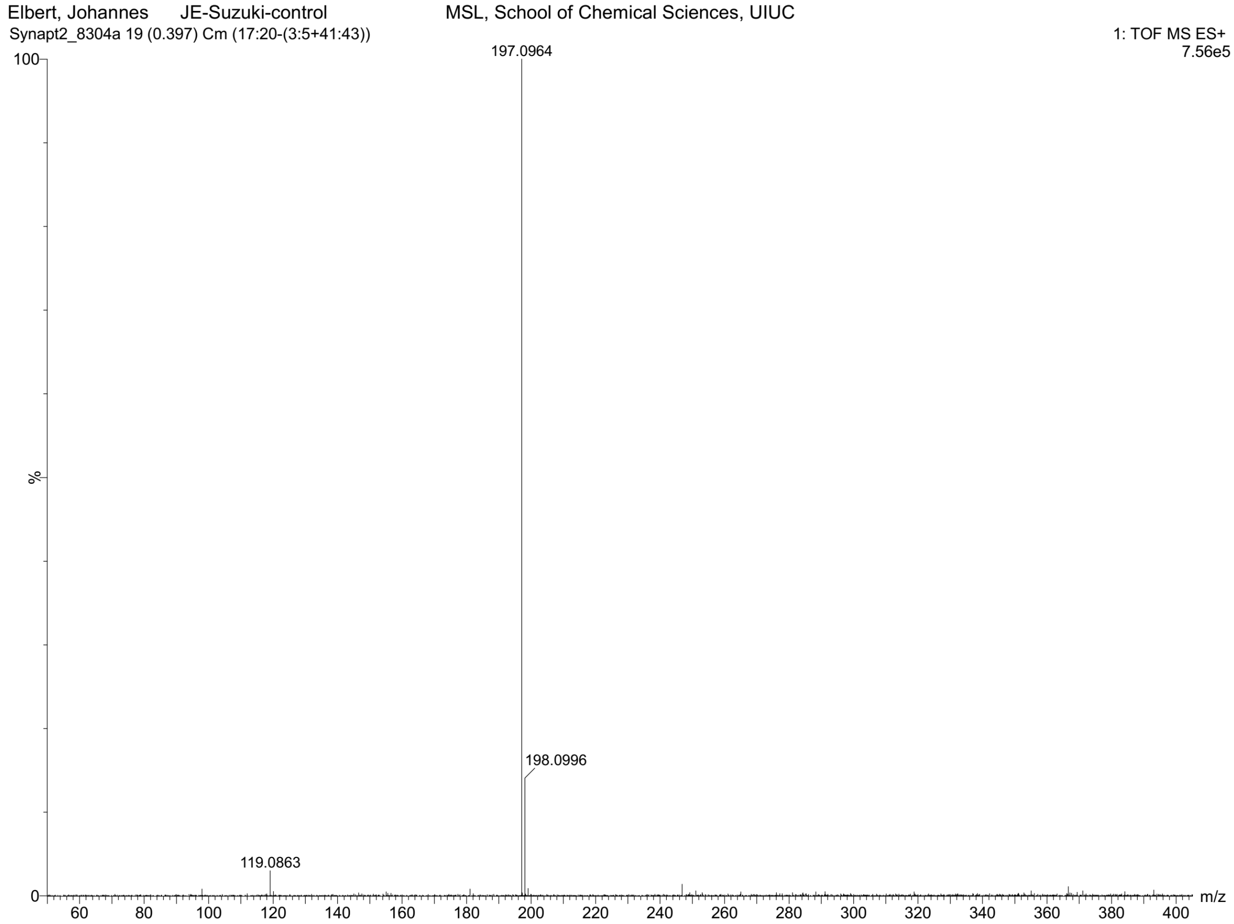

Figure S48. HR-ESI-MS of isolated 4-acetylbiphenyl synthesized using fresh catalyst.

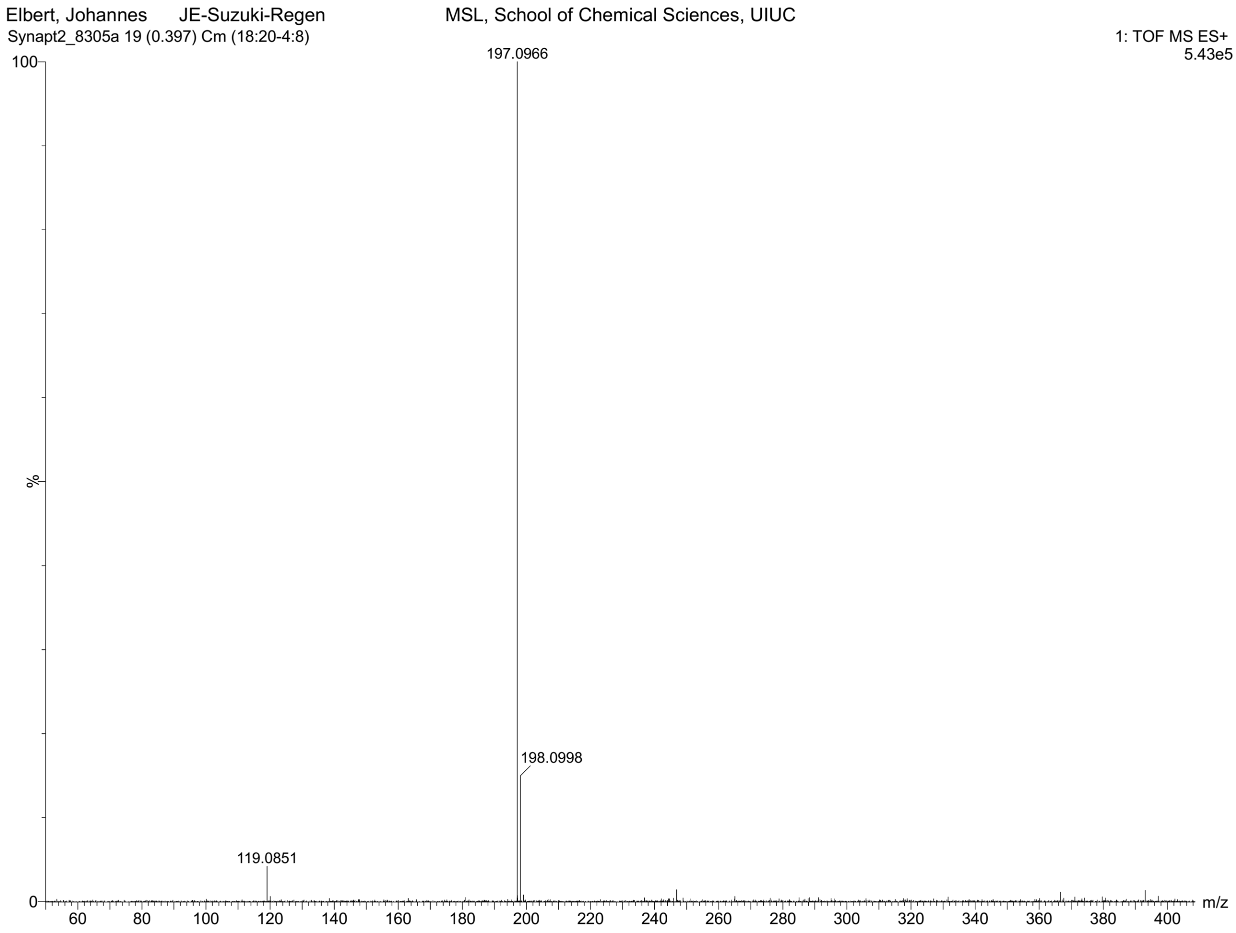

Figure S49. HR-ESI-MS of isolated 4-acetylbiphenyl synthesized using recycled catalyst.

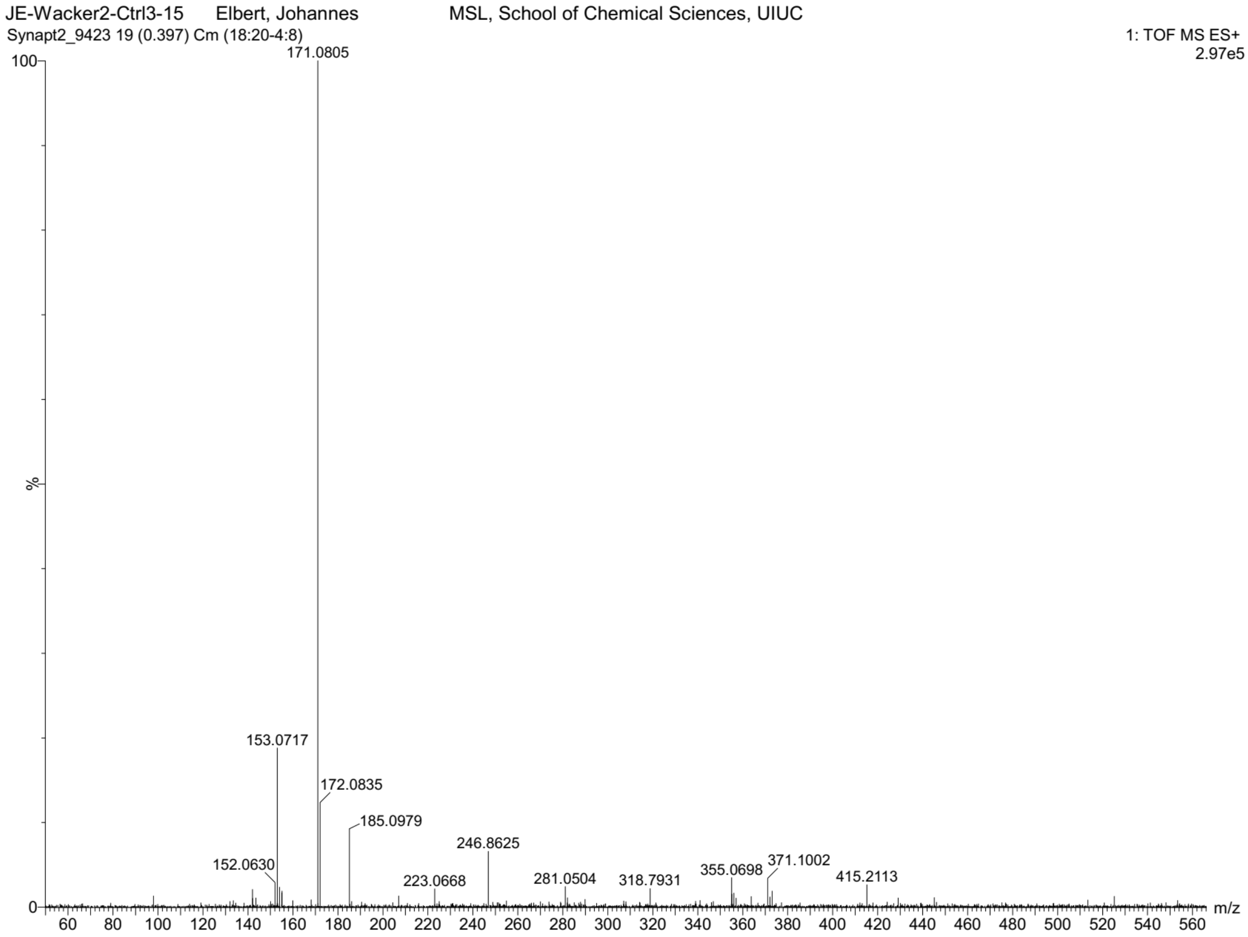

Figure S50. **HR-ESI-MS of isolated 2-acetonaphtone synthesized using fresh catalyst.**

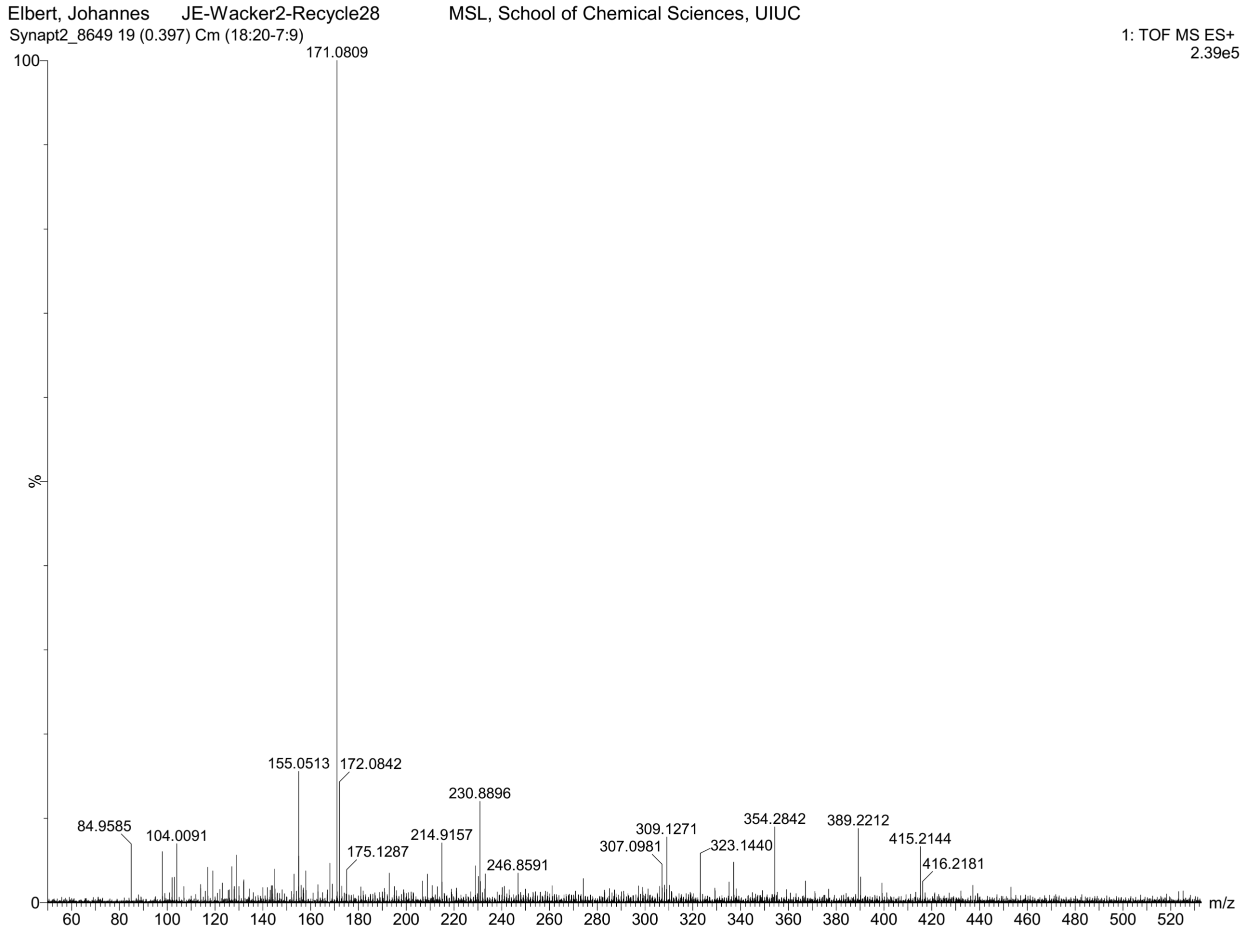

Figure S51. HR-ESI-MS of isolated 2-acetonaphtone synthesized using recycled catalyst.

15. LC-MS Data

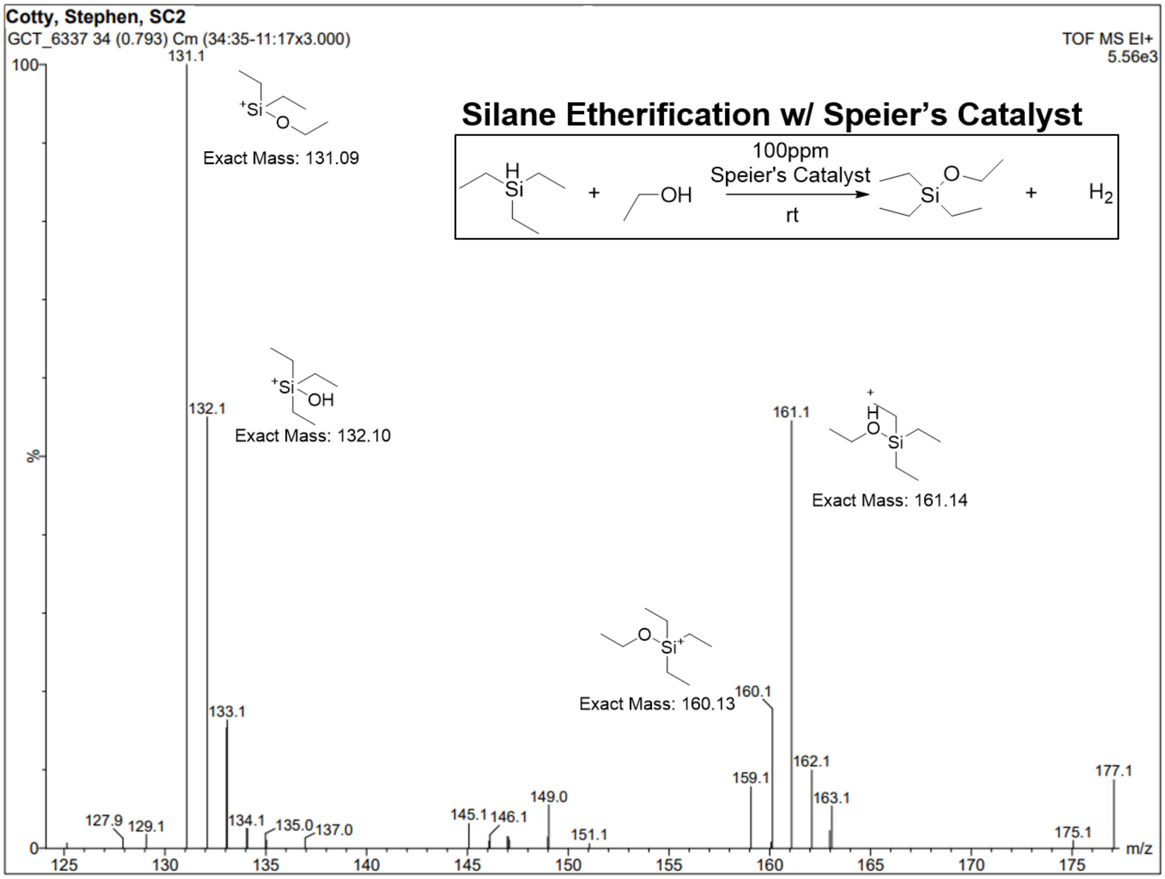

Figure S52. **EI+ mass spectra of silane etherification products obtained from the addition of 100 ppm Speier's catalyst to 2mL ethanol and 1mL triethylsilane after 8 minutes at 20 ºC.**

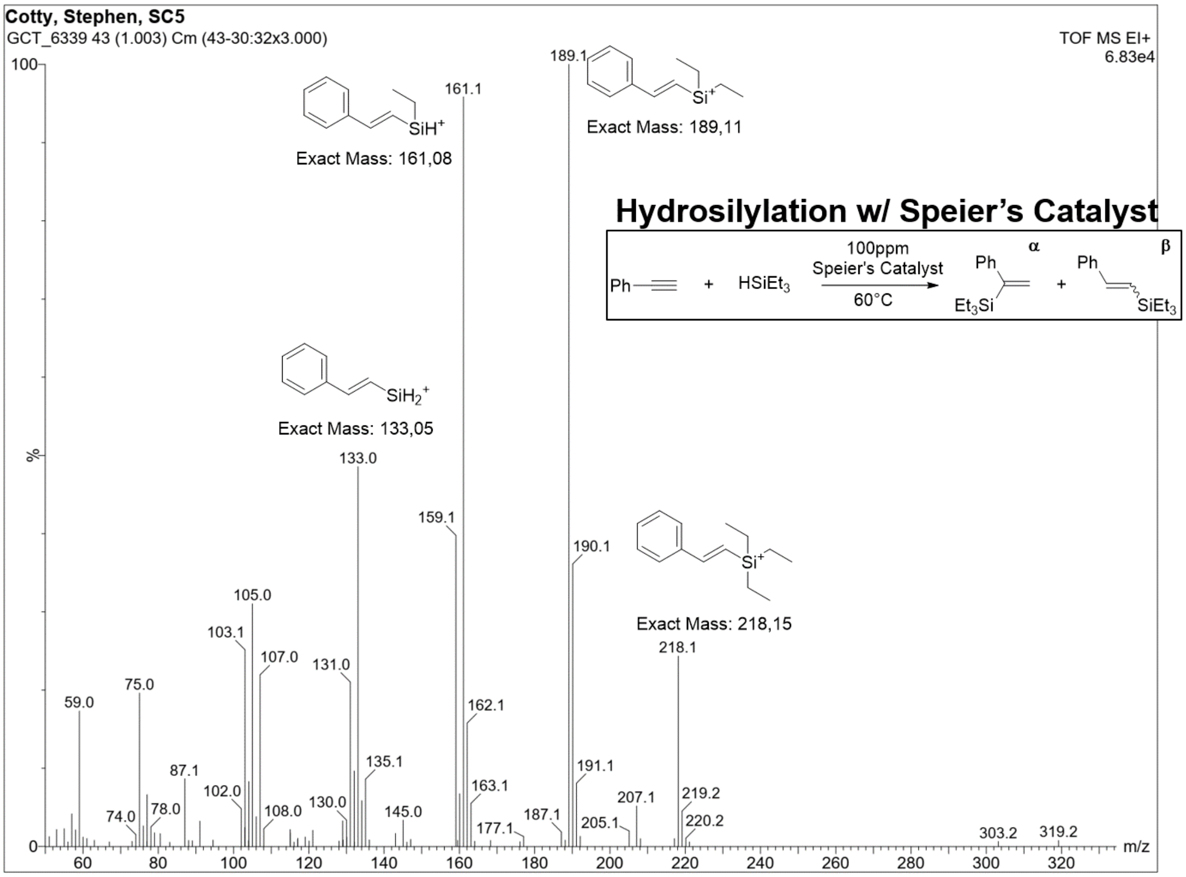

Figure S53. EI+ mass spectra of hydrosilylation products obtained from the addition of 100 ppm Speier's catalyst to 1 mL phenylacetylene, 1 mL triethylsilane, and 1 mL acetonitrile after 24 hours at 60 ºC.

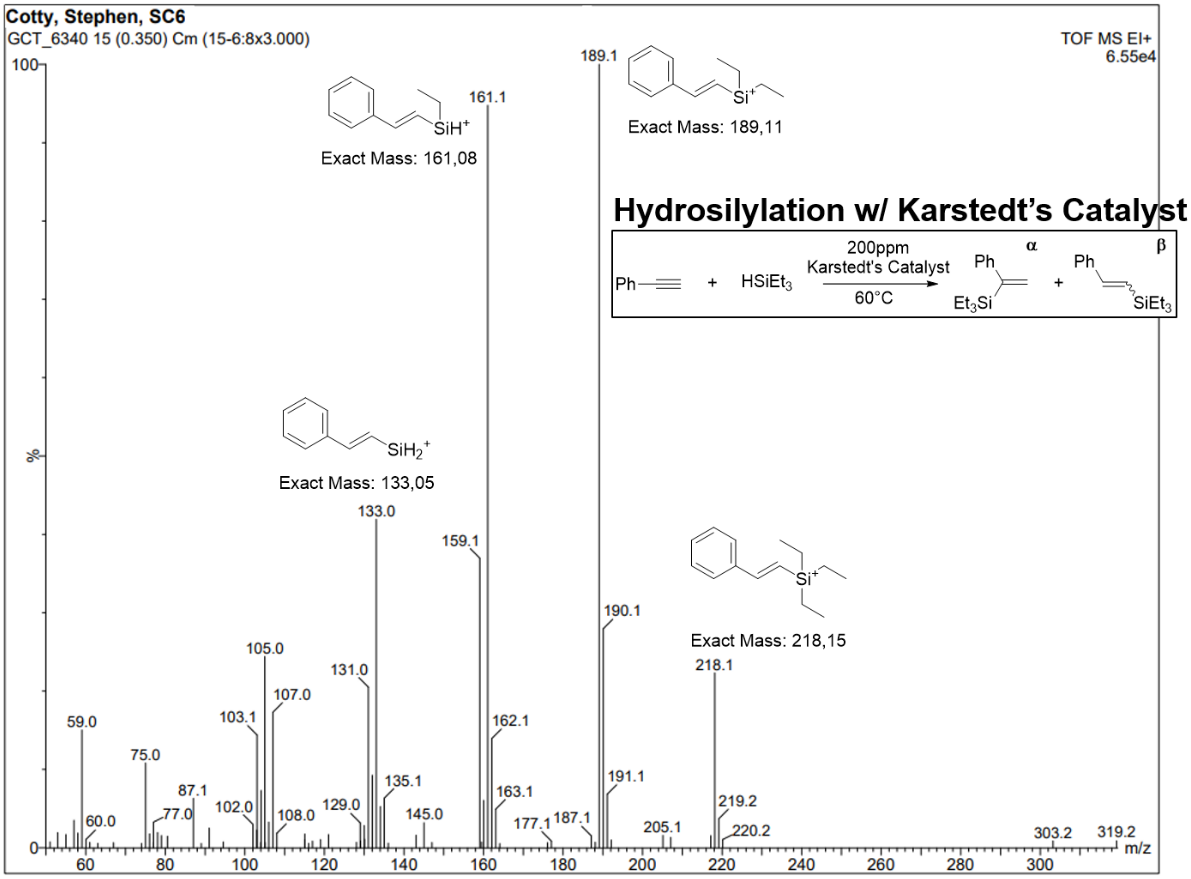

Figure S54. EI+ mass spectra of hydrosilylation products obtained from the addition of 200 ppm Karstedt's catalyst to 1mL phenylacetylene, 1mL triethylsilane, and 1mL acetonitrile after 24 hours at 60 Celsius.

16. TOF-SIMS Data

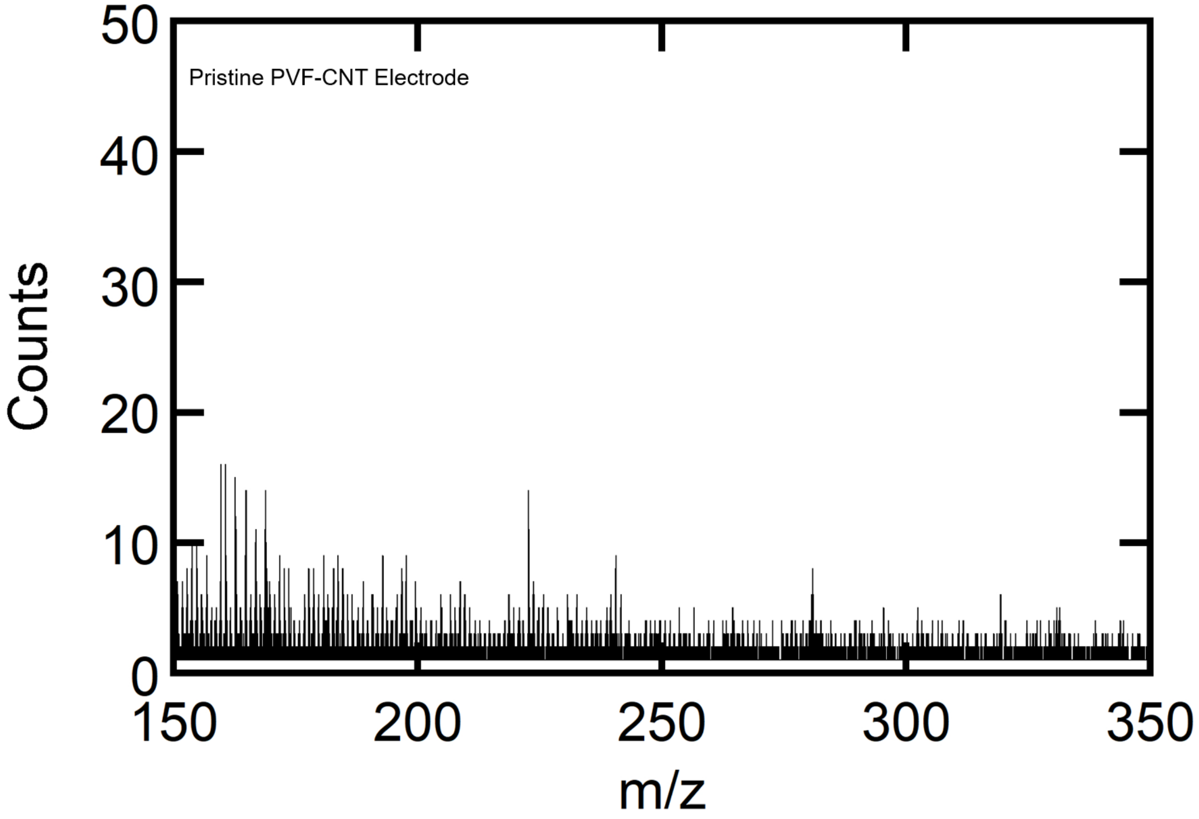

Figure S55. TOF-SIMS mass spectra of pristine PVF-CNT coated carbon paper electrode surface.

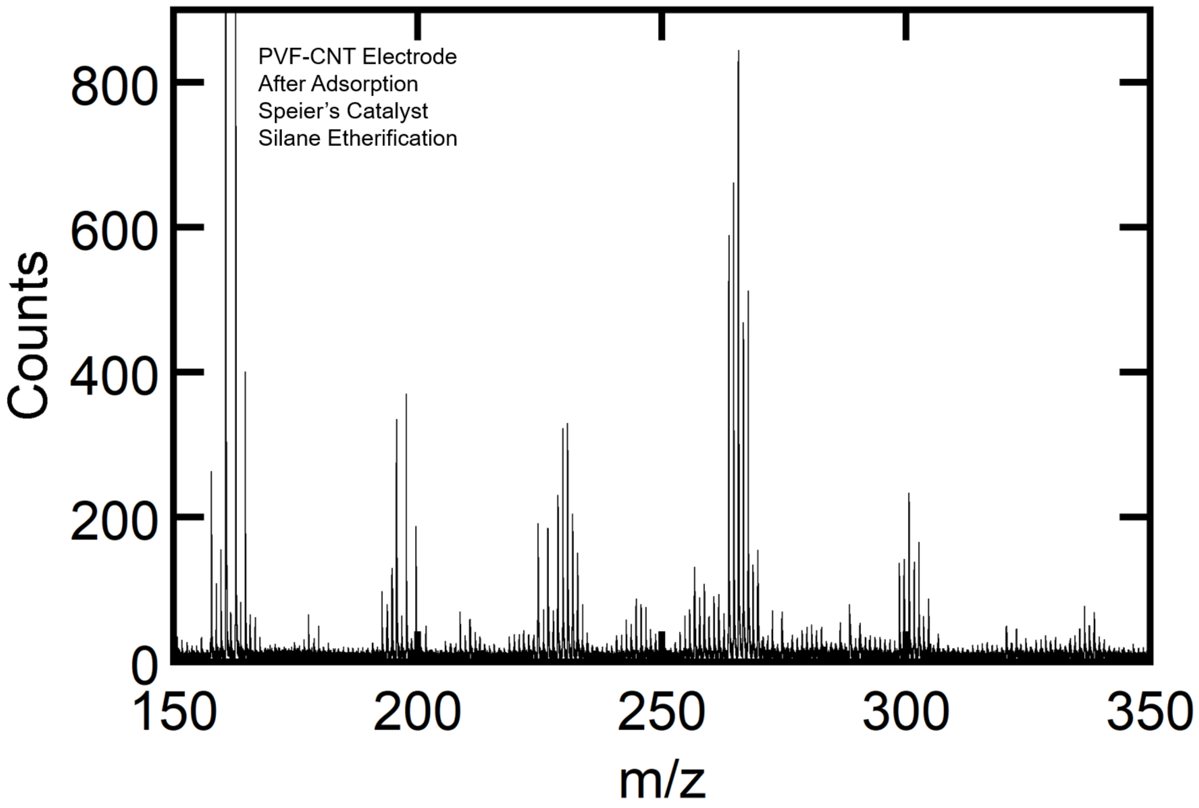

Figure S56. TOF-SIMS mass spectra of PVF-CNT electrode after adsorption of Speier’s catalyst from silane etherification reaction.

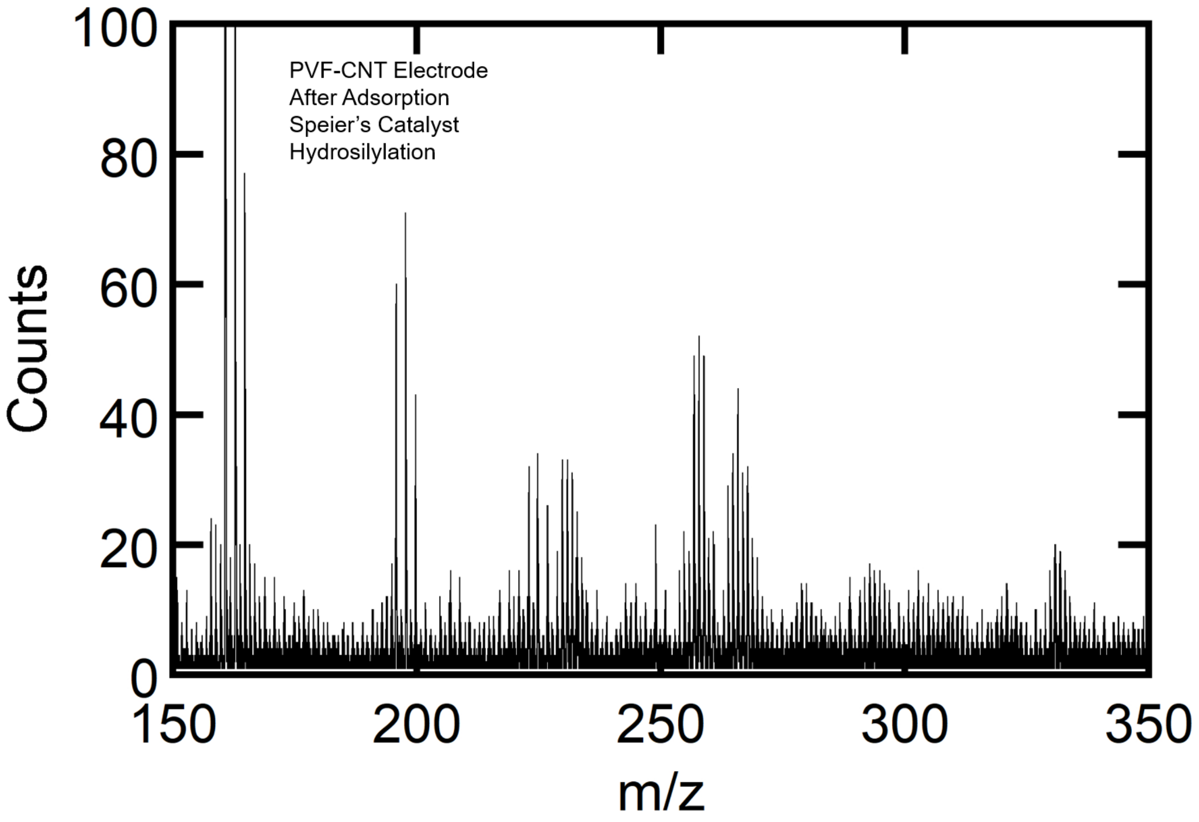

Figure S57. TOF-SIMS mass spectra of PVF-CNT electrode after adsorption of Speier's catalyst from hydrosilylation reaction.

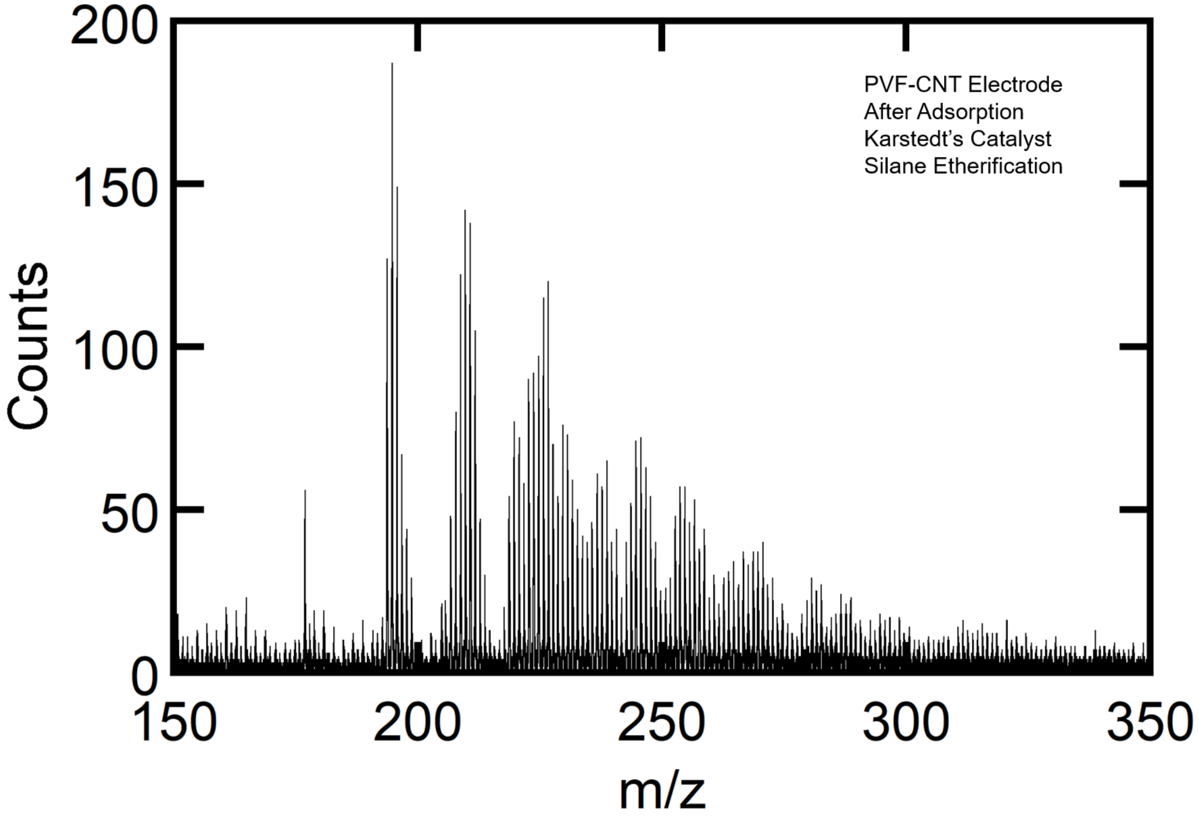

Figure S58. TOF-SIMS mass spectra of PVF-CNT electrode after adsorption of Karstedt's catalyst from silane etherification reaction.

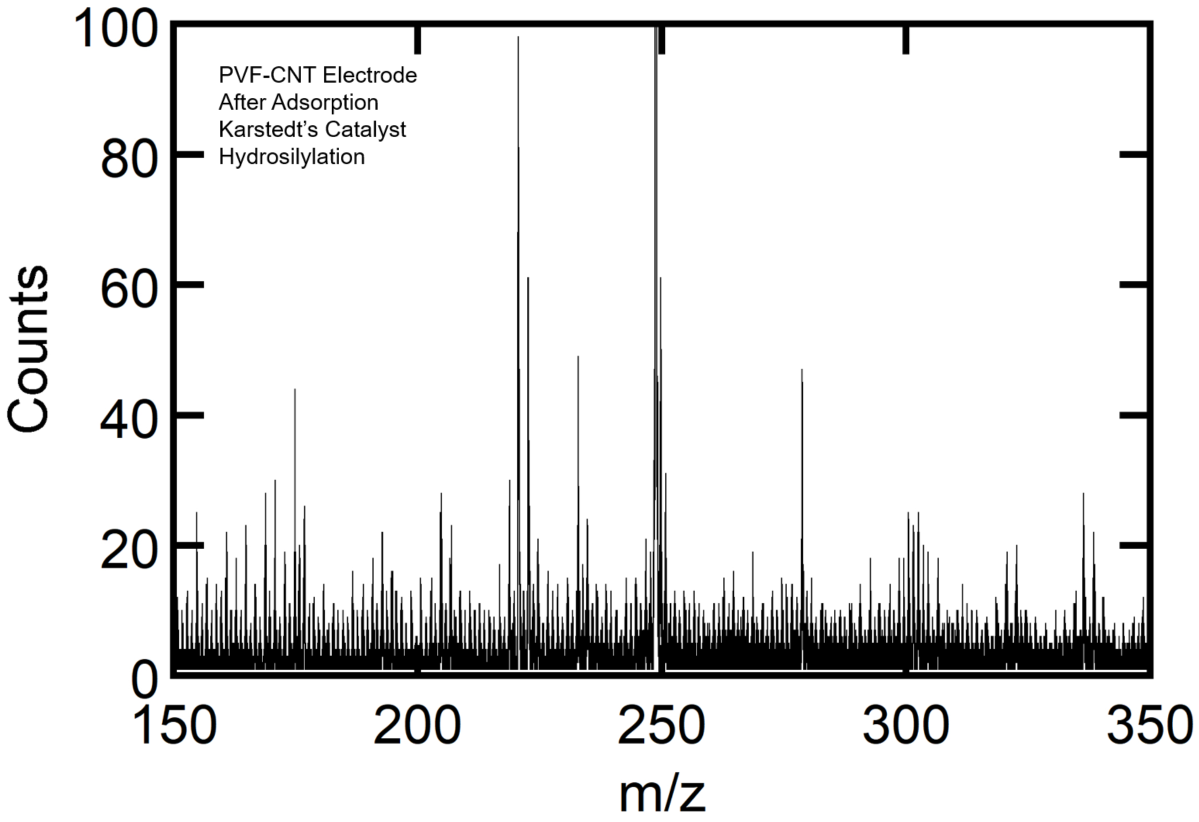

Figure S59. TOF-SIMS mass spectra of PVF-CNT electrode after adsorption of Karstedt's catalyst from hydrosilylation reaction.

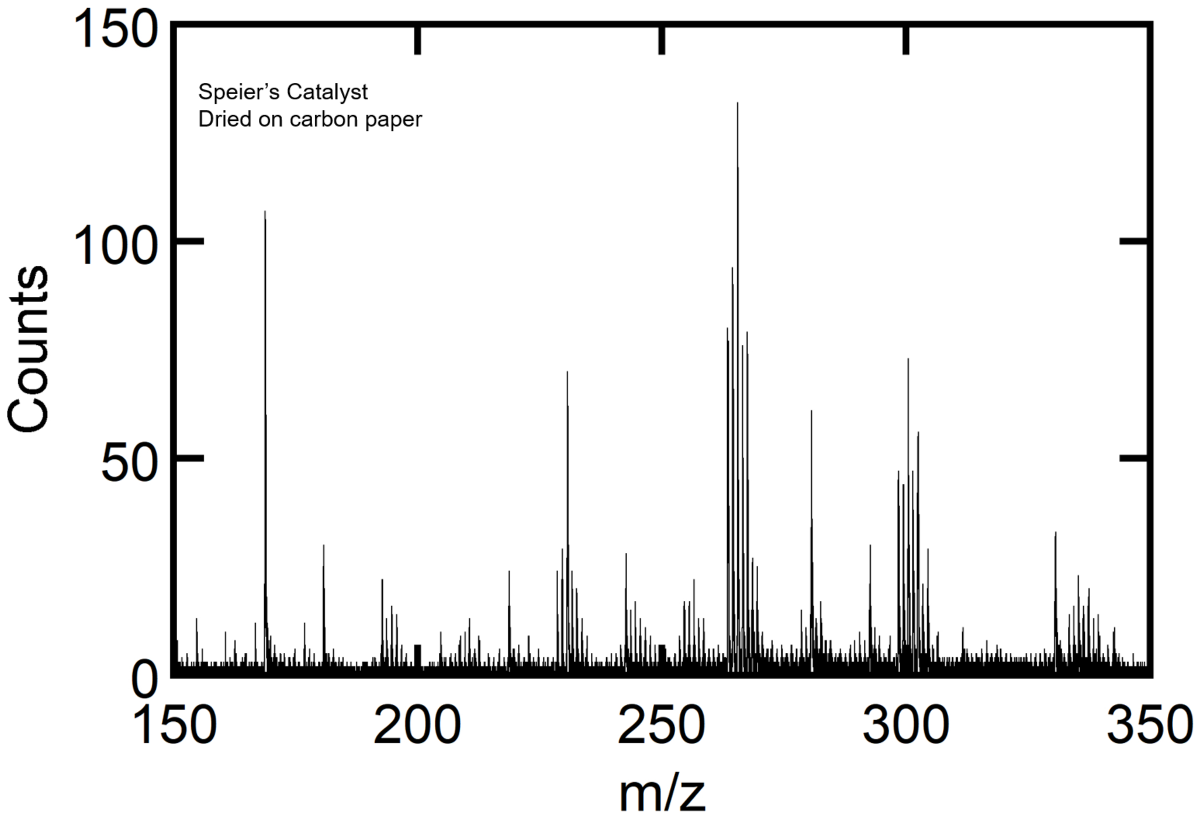

Figure S60. TOF-SIMS mass spectra of Speier's catalyst solution dried on carbon paper.

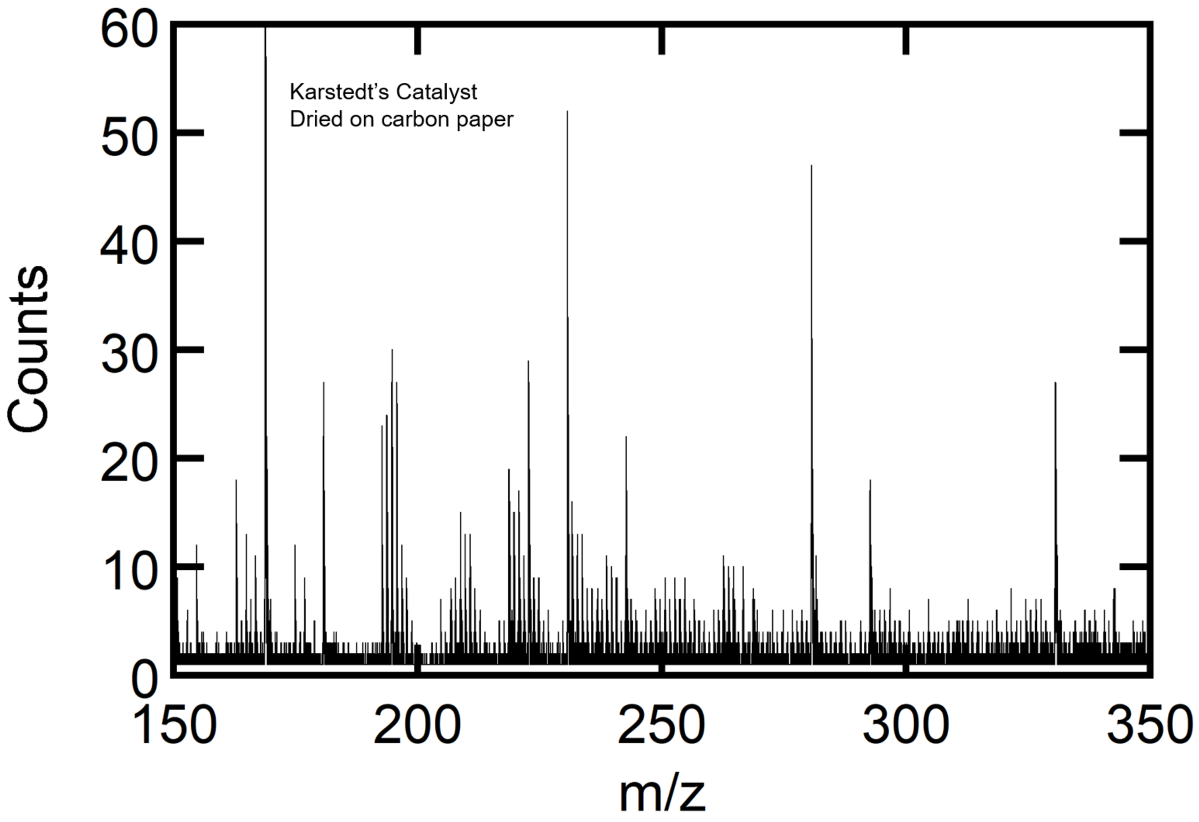

Figure S61. TOF-SIMS mass spectra of Karstedt's catalyst solution dried on carbon paper.

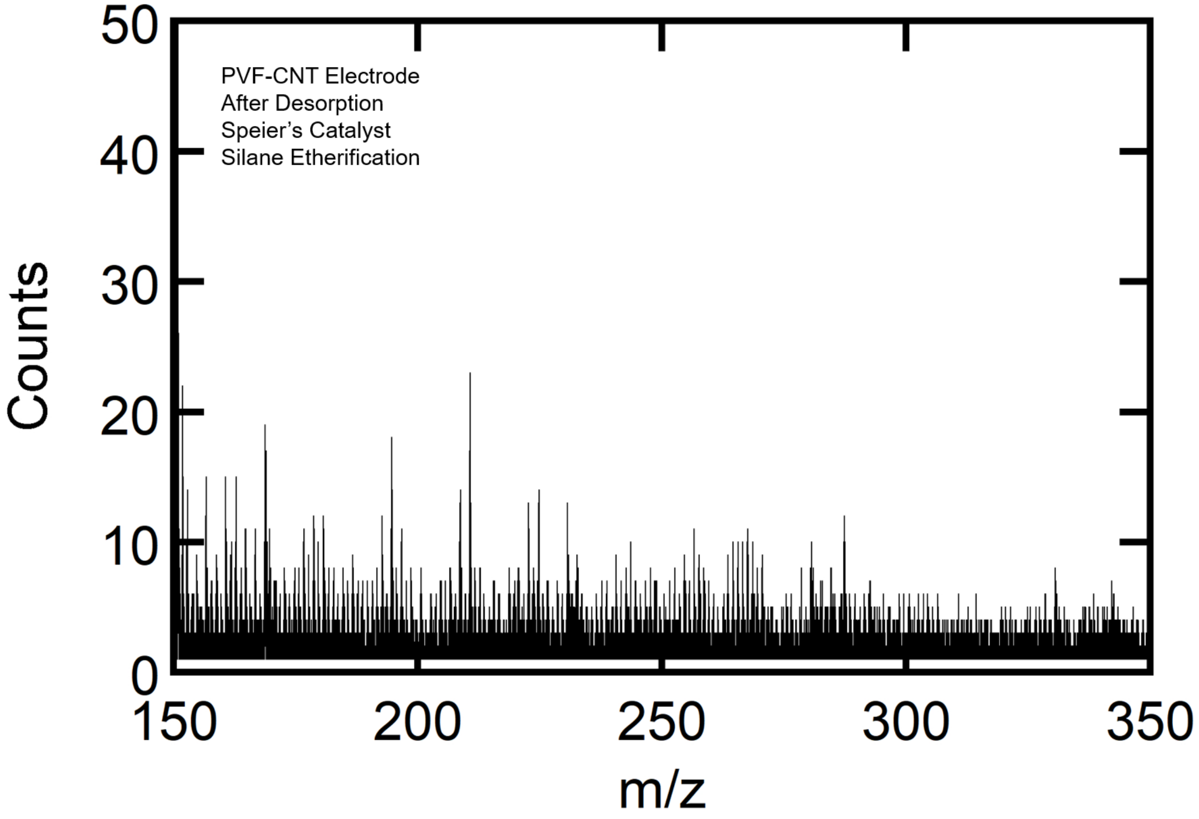

Figure S62. TOF-SIMS mass spectra of PVF-CNT electrode after desorption of Speier’s catalyst to silane etherification reactants.

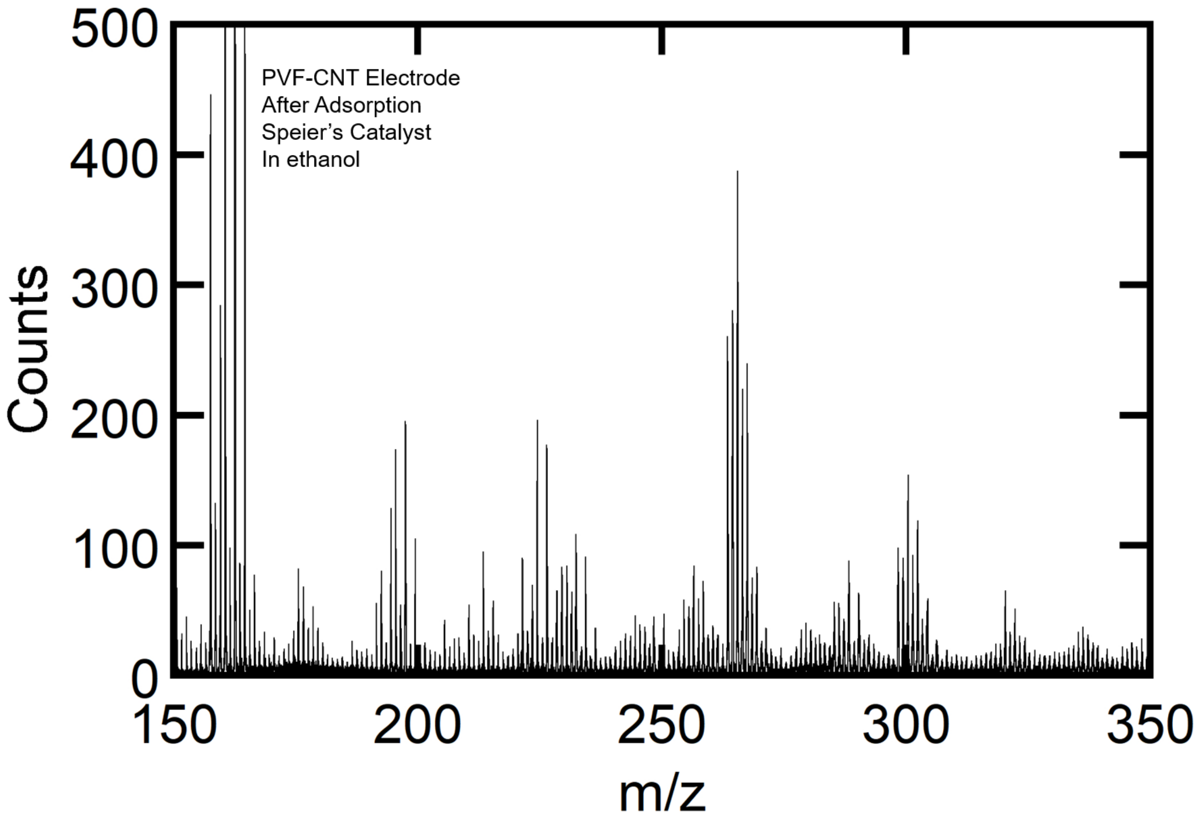

Figure S63. TOF-SIMS mass spectra of PVF-CNT electrode after adsorption of Speier's catalyst from ethanol.

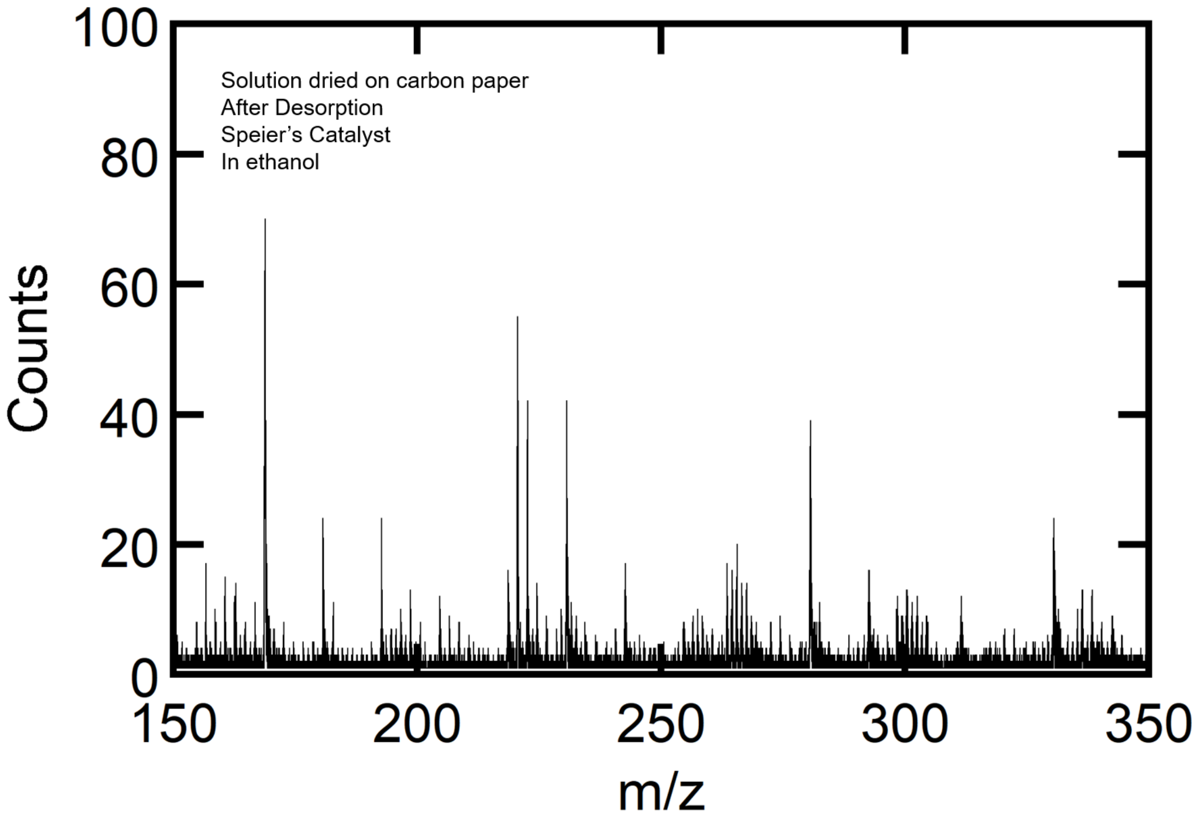

Figure S64. **TOF-SIMS mass spectra of sample of solution after Speier's catalyst desorption in ethanol dried onto carbon paper.**

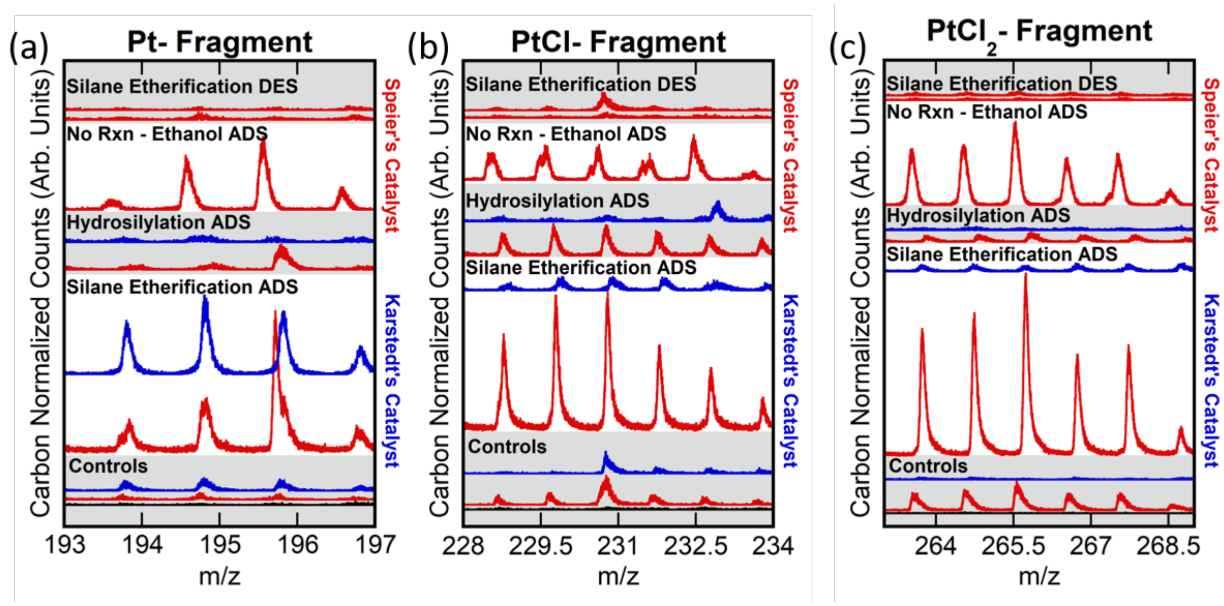

Figure S65. TOF-SIMS spectra focused on the presence of atomic (a) Pt, (b) PtCl, and (c) $PtCl_2$ fragments. Sample description from bottom to top: (black) pristine PVF-CNT electrode, (red) Speier's catalyst solution dried on carbon paper, (blue) Karstedt's catalyst solution dried on carbon paper, (red) PVF-CNT electrode after adsorption of Speier's catalyst from silane etherification reaction, (blue) PVF-CNT electrode after adsorption of Karstedt's catalyst from silane etherification reaction, (red) PVF-CNT electrode after adsorption of Speier's catalyst from hydrosilylation reaction, (blue) PVF-CNT electrode after adsorption of Karstedt's catalyst from hydrosilylation reaction, (red) PVF-CNT electrode after adsorption of Speier's catalyst from ethanol, (red) PVF-CNT electrode after desorption of Speier's catalyst to silane etherification reactants, (red) sample of the solution after Speier's catalyst desorption in ethanol dried onto carbon paper.

17. GC-MS Data

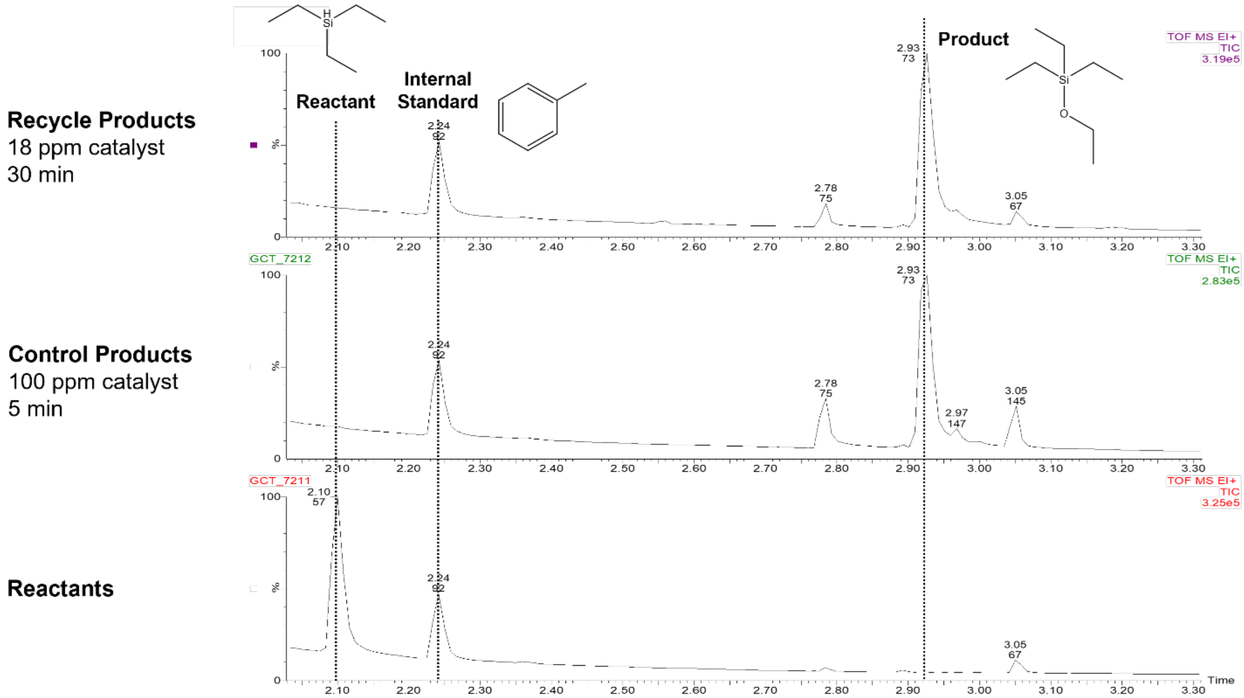

Figure S66. GC-MS chromatograph of silane etherification reactants, products from reaction using as received Speier's catalyst, and products from reaction where Speier's catalyst was electrochemically recycled using PVF adsorption.

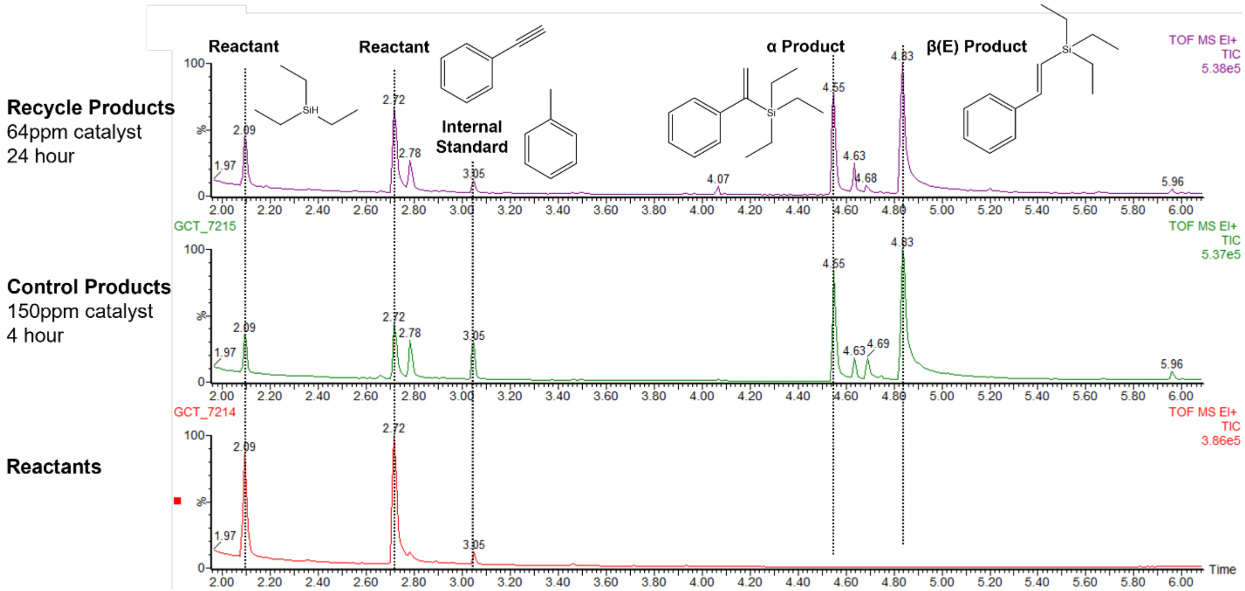

Figure S67. GC-MS chromatograph of hydrosilylation reactants, products from reaction using as received Speier's catalyst, and products from reaction where Speier's catalyst was electrochemically recycled using PVF adsorption.

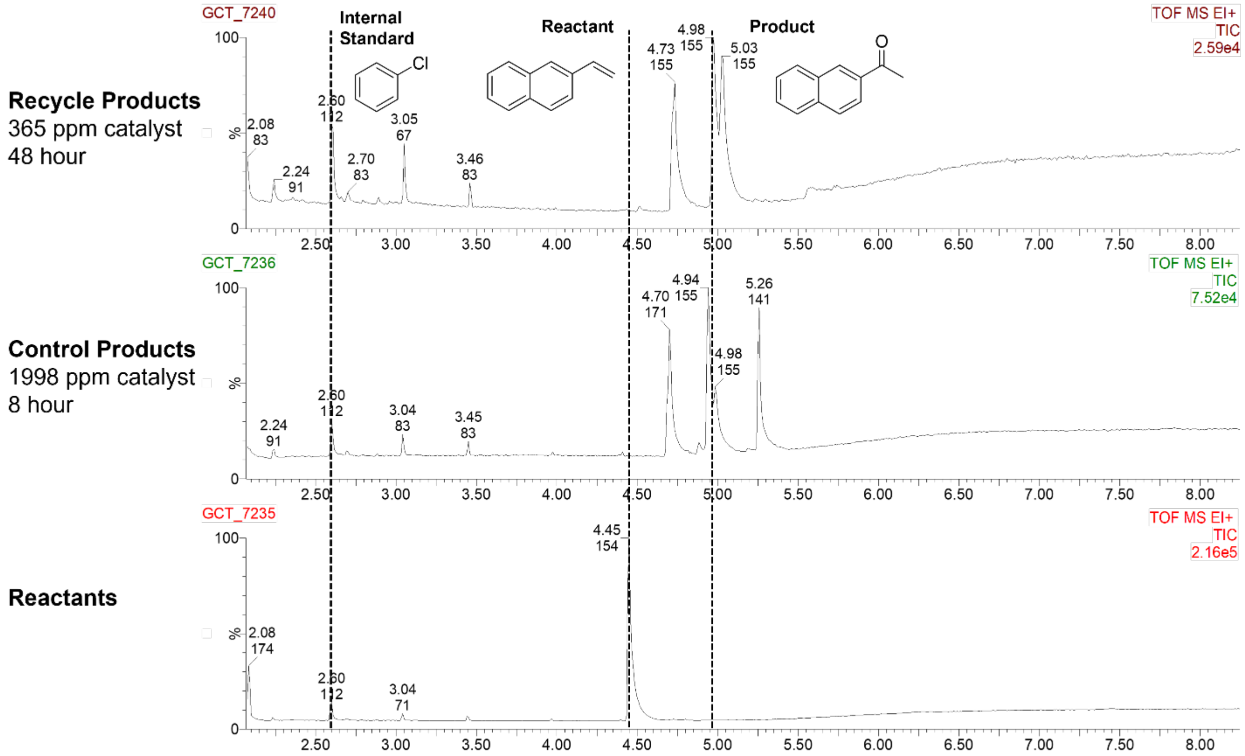

Figure S68. GC-MS chromatograph of Wacker oxidation reactants, products from reaction using as received Speier's catalyst, and products from reaction where $PdCl_2$ catalyst was electrochemically recycled using PVF adsorption.

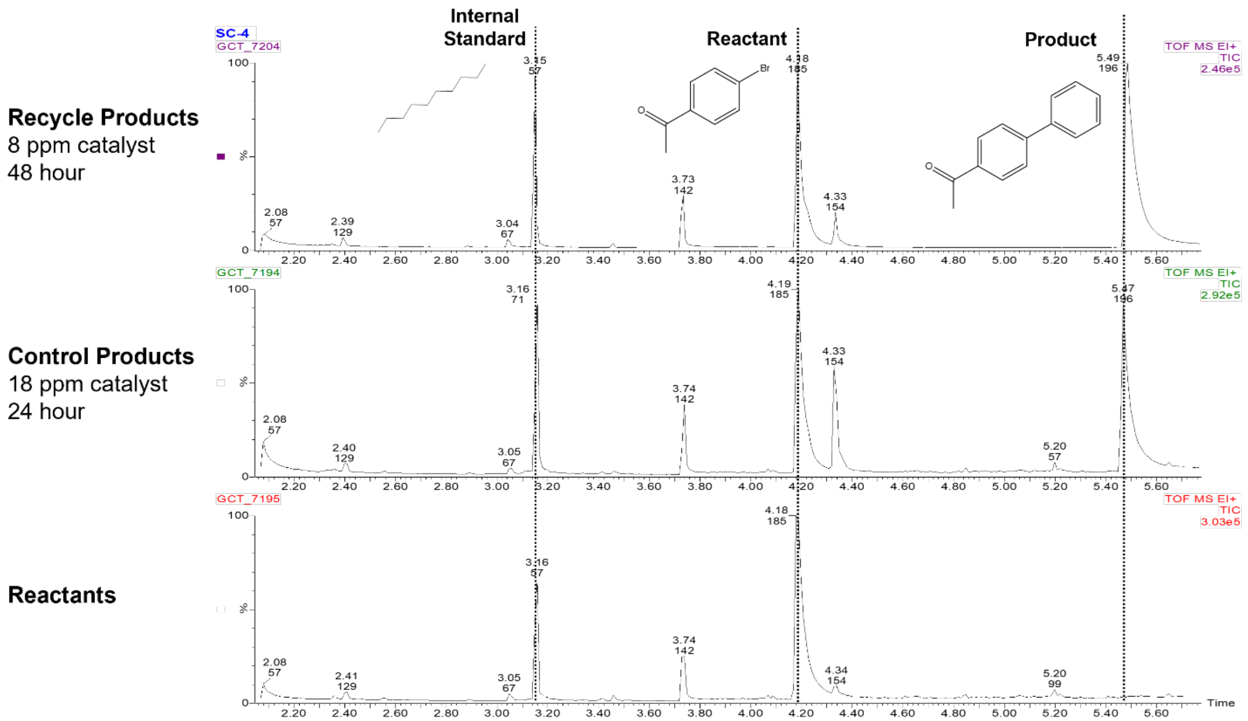

Figure S69. **GC-MS chromatograph of Suzuki cross-coupling reactants, products from reaction using as received Speier's catalyst, and products from reaction where Speier's catalyst was electrochemically recycled using PVF adsorption.**

REFERENCES AND NOTES

1. C. A. Tolman, J. P. Jesson, Homogeneous catalysis. *Science* **181**, 501–505 (1973).

2. D. J. Cole-Hasmilton, Homogeneous catalysis - New approaches to catalyst separation, recovery, and recycling. *Science* **299**, 1702–1706 (2003).

3. B. Cornils, W. A. Herrmann, *Applied Homogeneous Catalysis with Organometallic Compounds: A Comprehensive Handbook in Two Volumes* (Wiley-VCH, 1996).

4. J. A. Hueffel, T. Sperger, I. Funes-Ardoiz, J. S. Ward, K. Rissanen, F. Schoenebeck, Accelerated dinuclear palladium catalyst identification through unsupervised machine learning. *Science* **374**, 1134–1140 (2021).

5. S. Park, M. Brookhart, Hydrosilylation of carbonyl-containing substrates catalyzed by an electrophilic η1-silane iridium(III) complex. *Organometallics* **29**, 6057–6064 (2010).

6. M. Benaglia, in *Recoverable and Recyclable Catalysts* (Wiley, ed. 1, 2009), pp. xviii, 471 p.

7. N. D. Knofel, H. Rothfuss, J. Willenbacher, C. Barner-Kowollik, P. W. Roesky, Platinum(II)-crosslinked single-chain nanoparticles: An approach towards recyclable homogeneous catalysts. *Angew. Chem. Int. Ed.* **56**, 4950–4954 (2017).

8. B. Cornils, W. A. Herrmann, Concepts in homogeneous catalysis: The industrial view. *J. Catal.* **216**, 23–31 (2003).

9. D. Troegel, J. Stohrer, Recent advances and actual challenges in late transition metal catalyzed hydrosilylation of olefins from an industrial point of view. *Coordin. Chem. Rev.* **255**, 1440–1459 (2011).

10. R. J. Hofmann, M. Vlatkovic, F. Wiesbrock, Fifty years of hydrosilylation in polymer science: A review of current trends of low-cost transition-metal and metal-free catalysts, non-thermally triggered hydrosilylation reactions, and industrial applications. *Polymers* **9**, 534 (2017).

11. B. Marciniec, in *Hydrosilylation of Alkenes and Their Derivatives* (Hydrosilylation: A Comprehensive Review on Recent Advances, Springer, 2009), vol. 1, pp. 3–51.

12. L. D. de Almeida, H. Wang, K. Junge, X. Cui, M. Beller, Recent advances in catalytic hydrosilylations: Developments beyond traditional platinum catalysts. *Angew. Chem. Int. Ed. Engl.* **60**, 550–565 (2021).

13. C. K. Blasius, H. Wadepohl, L. H. Gade, NNN-cobalt(II) pincer complexes: Paramagnetic NMR spectroscopy in solution and application as hydrosilylation catalysts. *Eur. J. Inorg. Chem.* **2020**, 2335–2342 (2020).

14. W. J. Teo, C. Wang, Y. W. Tan, S. Z. Ge, Cobalt-catalyzed *Z*-selective hydrosilylation of terminal alkynes. *Angew. Chem. Int. Ed.* **56**, 4328–4332 (2017).

15. Y. F. Wei, S. X. Liu, H. Mueller-Bunz, M. Albrecht, Synthesis of triazolylidene nickel complexes and their catalytic application in selective aldehyde hydrosilylation. *ACS Catal.* **6**, 8192–8200 (2016).

16. Y. Nakajima, S. Shimada, Hydrosilylation reaction of olefins: Recent advances and perspectives. *RSC Adv.* **5**, 20603–20616 (2015).

17. H. B. Li, C. C. C. J. Seechurn, T. J. Colacot, Development of preformed Pd catalysts for cross-coupling reactions, beyond the 2010 nobel prize. *ACS Catal.* **2**, 1147–1164 (2012).

18. K. C. Nicolaou, E. J. Sorensen, in *Classics in Total Synthesis: Targets, Strategies, Methods* (Wiley-VCH, 1996), pp. xxiii, 798 p.

19. J. Magano, J. R. Dunetz, Recent large-scale applications of transition metal-catalyzed couplings for the synthesis of pharmaceuticals. *Rsc Catal. Ser.* 697–778 (2015).

20. D. J. Cole-Hamilton, R. P. Tooze, in *Catalyst Separation, Recovery and Recycling: Chemistry and Process Design* (Catalysis by Metal Complexes, Springer, 2006), pp. ix, 248 p.

21. J. Hagen, in *Industrial Catalysis: A Practical Approach* (John Wiley & Sons, 2006), pp. 1–507.

22. A. E. Schweizer, G. T. Kerr, Thermal-decomposition of hexachloroplatinic acid. *Inorg. Chem.* **17**, 2326–2327 (1978).

23. P. W. N. M. v. Leeuwen, J. C. Chadwick, *Homogeneous Catalysts. Activity—Stability—Deactivation* (Wiley-VCH, 2011), pp. 1 online resource (xiii, 404 pages).

24. G. Duca, in *Homogeneous Catalysis with Metal Complexes: Fundamentals and Applications* (Springer Series in Chemical Physics, Springer, 2012), pp. 1 online resource.

25. J. L. Barton, Electrification of the chemical industry. *Science* **368**, 1181–1182 (2020).

26. D. S. Sholl, R. P. Lively, Seven chemical separations to change the world. *Nature* **532**, 435–437 (2016).

27. X. Su, Electrochemical interfaces for chemical and biomolecular separations. *Curr. Opin. Colloid. Interface Sci.* **46**, 77–93 (2020).

28. M. E. Suss, S. Porada, X. Sun, P. M. Biesheuvel, J. Yoon, V. Presser, Water desalination via capacitive deionization: What is it and what can we expect from it? *Energ. Environ. Sci.* **8**, 2296–2319 (2015).

29. R. Candeago, K. Kim, H. Vapnik, S. Cotty, M. Aubin, S. Berensmeier, A. Kushima, X. Su, Semiconducting polymer interfaces for electrochemically assisted mercury remediation. *ACS Appl. Mater. Interfaces* **12**, 49713–49722 (2020).

30. X. Su, K.-J. Tan, J. Elbert, C. Ruttiger, M. Gallei, T. F. Jamison, T. A. Hatton, Asymmetric Faradaic systems for selective electrochemical separations. *Energ. Environ. Sci.* **10**, 1272–1283 (2017).

31. X. Su, L. Bromberg, K. J. Tan, T. F. Jamison, L. P. Padhye, T. A. Hatton, Electrochemically mediated reduction of nitrosamines by hemin-functionalized redox electrodes. *Environ. Sci. Tech. Lett.* **4**, 161–167 (2017).

32. F. Y. Cheng, J. Liang, Z. L. Tao, J. Chen, Functional materials for rechargeable batteries. *Adv. Mater.* **23**, 1695–1715 (2011).

33. K. Kim, S. J. Lee, D. Y. Kim, C. Y. Yoo, J. W. Choi, J. N. Kim, Y. Woo, H. C. Yoon, J. I. Han, Electrochemical synthesis of ammonia from water and nitrogen: A lithium-mediated approach using lithium-ion conducting glass ceramics. *ChemSusChem* **11**, 120–124 (2018).

34. A. K. Dutta, F. Neese, R. Izsak, Speeding up equation of motion coupled cluster theory with the chain of spheres approximation. *J. Chem. Phys.* **144**, 034102 (2016).

35. X. Su, A. Kushima, C. Halliday, J. Zhou, J. Li, T. A. Hatton, Electrochemically-mediated selective capture of heavy metal chromium and arsenic oxyanions from water. *Nat. Commun.* **9**, 4701 (2018).

36. K. Kim, S. Cotty, J. Elbert, R. L. Chen, C. H. Hou, X. Su, Asymmetric redox-polymer interfaces for electrochemical reactive separations: Synergistic capture and conversion of arsenic. *Adv. Mater.* **32**, 1906877 (2020).

37. K. Kim, P. B. Medina, J. Elbert, E. Kayiwa, R. D. Cusick, Y. J. Men, X. Su, Molecular tuning of redox-copolymers for selective electrochemical remediation. *Adv. Funct. Mater.* **30**, 2004635 (2020).

38. X. Su, J. Hubner, M. J. Kauke, L. Dalbosco, J. Thomas, C. C. Gonzalez, E. Zhu, M. Franzreb, T. F. Jamison, T. A. Hatton, Redox interfaces for electrochemically controlled protein-surface interactions: Bioseparations and heterogeneous enzyme catalysis. *Chem. Mater.* **29**, 5702–5712 (2017).

39. J. L. Speier, J. A. Webster, G. H. Barnes, The addition of silicon hydrides to olefinic double bonds. Part II. The use of group-VIII metal catalysts. *J. Am. Chem. Soc.* **79**, 974–979 (1957).

40. J. E. Baeckvall, B. Akermark, S. O. Ljunggren, Stereochemistry and mechanism for the palladium(II)-catalyzed oxidation of ethene in water (the Wacker process). *J. Am. Chem. Soc.* **101**, 2411–2416 (1979).

41. N. Miyaura, A. Suzuki, Palladium-catalyzed cross-coupling reactions of organoboron compounds. *Chem. Rev.* **95**, 2457–2483 (1995).

42. R. A. Benkeser, J. Kang, The composition of speier's catalyst. *J. Organomet. Chem.* **185**, C9–C12 (1980).

43. J. Stein, L. N. Lewis, Y. Gao, R. A. Scott, In situ determination of the active catalyst in hydrosilylation reactions using highly reactive Pt(0) catalyst precursors. *J. Am. Chem. Soc.* **121**, 3693–3703 (1999).

44. P. W. N. M. Leeuwen, Oxidation with dioxygen, in *Homogeneous Catalysis: Understanding the Art* (Springer, 2004), pp. 319–336.

45. R. A. Fernandes, A. K. Jha, P. Kumar, Recent advances in Wacker oxidation: From conventional to modern variants and applications. *Cat. Sci. Technol.* **10**, 7448–7470 (2020).

46. C. Amatore, A. Jutand, Anionic Pd(0) and Pd(II) intermediates in palladium-catalyzed Heck and cross-coupling reactions. *Acc. Chem. Res.* **33**, 314–321 (2000).

47. A. A. Thomas, S. E. Denmark, Pre-transmetalation intermediates in the Suzuki-Miyaura reaction revealed: The missing link. *Science* **352**, 329–332 (2016).

48. F. Schroeter, J. Soellner, T. Strassner, Cross-coupling catalysis by an anionic palladium complex. *ACS Catal.* **7**, 3004–3009 (2017).

49. M. Peuckert, H. P. Bonzel, Characterization of oxidized platinum surfaces by x-ray photoelectron-spectroscopy. *Surf. Sci.* **145**, 239–259 (1984).

50. A. Romanchenko, M. Likhatski, Y. Mikhlin, X-ray photoelectron spectroscopy (XPS) study of the products formed on sulfide minerals upon the interaction with aqueous platinum (IV) chloride complexes. *Minerals* **8**, 578 (2018).

51. H. M. Yasin, G. Denuault, D. Pletcher, Studies of the electrodeposition of platinum metal from a hexachloroplatinic acid bath. *J. Electroanal. Chem.* **633**, 327–332 (2009).

52. M. E. Baumgartner, D. R. Gabe, Palladium-iron alloy electrodeposition. Part I Single metal systems. *Trans. IMF* **78**, 11–16 (2000).

53. C. Capello, U. Fischer, K. Hungerbühler, What is a green solvent? A comprehensive framework for the environmental assessment of solvents. *Green Chem.* **9**, 927–934 (2007).

54. M. P. Sibi, Hydrogen hexachloroplatinate(IV), in *Encyclopedia of Reagents for Organic Synthesis* (2001).

55. M. G. Voronkov, V. B. Pukhnarevich, N. I. Ushakova, I. I. Tsykhanskaya, A. I. Albanov, V. Y. Vitkovskii, Dehydrocondensation of trialkylsilanes with acetylenes and monoorganylacetylenes. *Zh Obshch Khim* **55**, 94–100 (1985), pp. 1–8.

56. R. L. Reyes, M. Sato, T. Iwai, K. Suzuki, S. Maeda, M. Sawamura, Asymmetric remote C–H borylation of aliphatic amides and esters with a modular iridium catalyst. *Science* **369**, 970−974 (2020).

57. S. Y. Hong, Y. Park, Y. Hwang, Y. B. Kim, M. H. Baik, S. Chang, Selective formation of γ-lactams via C-H amidation enabled by tailored iridium catalysts. *Science* **359**, 1016–1021 (2018).

58. B. A. Vaughan, M. S. Webster-Gardiner, T. R. Cundari, T. B. Gunnoe, A rhodium catalyst for single-step styrene production from benzene and ethylene. *Science* **348**, 421–424 (2015).

59. J. Y. Cho, M. K. Tse, D. Holmes, R. E. Maleczka, M. R. Smith III, Remarkably selective iridium catalysts for the elaboration of aromatic C–H bonds. *Science* **295**, 305–308 (2002).

60. Z. G. Yin, Q. D. Zheng, S. C. Chen, D. D. Cai, L. Y. Zhou, J. Zhang, Bandgap tunable $Zn_{1-x}Mg_xO$ thin films as highly transparent cathode buffer layers for high-performance inverted polymer solar cells. *Adv. Energy Mater.* **4**, 1301404 (2014).

61. D. A. Baker, G. C. East, S. K. Mukhopadhyay, Synthesis and characterization of some disulfonyl azides as potential crosslinking agents for textile fibers. *J. Appl. Polym. Sci.* **79**, 1092–1100 (2001).

62. K. Lehmann, O. Yurchenko, G. Urban, Carbon nanowalls for oxygen reduction reaction in bio fuel cells. *J. Phys. Conf. Ser.* **557**, 012008 (2014).

63. J.-D. Lin, Q.-Y. Bi, L. Tao, T. Jiang, Y.-M. Liu, H.-Y. He, Y. Cao, Y.-D. Wang, Wettability-driven palladium catalysis for enhanced dehydrogenative coupling of organosilanes. *ACS Catal.* **7**, 1720–1727 (2017).

64. C. Xu, B. Huang, T. Yan, M. Cai, A recyclable and reusable K2PtCl4/Xphos-SO3Na/PEG-400/H2O system for highly regio- and stereoselective hydrosilylation of terminal alkynes. *Green Chem.* **20**, 391–397 (2018).

65. G. Kumar, J. R. Blackbur, R. G. Aldridge, W. E. Moddeman, M. M. Jones, Photoelectron spectroscopy of coordination compounds. II. Palladium complexes. *Inorg Chem* **11**, 296–300 (1972).

66. K. Plevova, B. Mudrakova, R. Sebesta, A practical three-step synthesis of vinylferrocene. *Synthesis Stuttgart* **50**, 760–763 (2018).

67. M. D. Hanwell, D. E. Curtis, D. C. Lonie, T. Vandermeersch, E. Zurek, G. R. Hutchison, Avogadro: An advanced semantic chemical editor, visualization, and analysis platform. *J. Chem.* **4**, 17 (2012).

68. A. Najibi, L. Goerigk, The nonlocal kernel in van der Waals density functionals as an additive correction: An extensive analysis with special emphasis on the B97M-V and ωB97M-V approaches. *J. Chem. Theory Comput.* **14**, 5725–5738 (2018).

69. F. Weigend, R. Ahlrichs, Balanced basis sets of split valence, triple zeta valence and quadruple zeta valence quality for H to Rn: Design and assessment of accuracy. *Phys. Chem. Chem. Phys.* **7**, 3297–3305 (2005).

70. M. Garcia-Rates, F. Neese, Effect of the solute cavity on the solvation energy and its derivatives within the framework of the gaussian charge scheme. *J. Comput. Chem.* **41**, 922–939 (2020).

71. F. Neese, F. Wennmohs, U. Becker, C. Riplinger, The ORCA quantum chemistry program package. *J. Chem. Phys.* **152**, 224108 (2020).

72. P. S. Albright, L. J. Gosting, Dielectric constants of the methanol-water system from 5 to 55°[1]. *J. Am. Chem. Soc.* **68**, 1061–1063 (1946).

73. J. Wyman, The dielectric constant of mixtures of ethyl alcohol and water from −5 to 40°. *J. Am. Chem. Soc.* **53**, 3292–3301 (1931).

74. M. J. Gilkey, A. V. Mironenko, D. G. Vlachos, B. J. Xu, Adipic acid production via metal-free selective hydrogenolysis of biomass-derived tetrahydrofuran-2,5-dicarboxylic acid. *ACS Catal.* **7**, 6619–6634 (2017).

75. S. Spicher, S. Grimme, Single-point Hessian calculations for improved vibrational frequencies and rigid-rotor-harmonic-oscillator thermodynamics. *J. Chem. Theory Comput.* **17**, 1701–1714 (2021).

76. S. Grimme, Supramolecular binding thermodynamics by dispersion-corrected density functional theory. *Chem. Eur. J.* **18**, 9955–9964 (2012).

77. R. F. Ribeiro, A. V. Marenich, C. J. Cramer, D. G. Truhlar, Use of solution-phase vibrational frequencies in continuum models for the free energy of solvation. *J. Phys. Chem. B* **115**, 14556–14562 (2011).

78. A. Altun, F. Neese, G. Bistoni, Effect of electron correlation on intermolecular interactions: A pair natural orbitals coupled cluster based local energy decomposition study. *J. Chem. Theory Comput.* **15**, 215–228 (2019).

79. Y. Guo, C. Riplinger, U. Becker, D. G. Liakos, Y. Minenkov, L. Cavallo, F. Neese, Communication: An improved linear scaling perturbative triples correction for the domain based local pair-natural orbital based singles and doubles coupled cluster method [DLPNO-CCSD(T)]. *J. Chem. Phys.* **148**, 011101 (2018).

80. G. L. Stoychev, A. A. Auer, F. Neese, Automatic generation of auxiliary basis sets. *J. Chem. Theory Comput.* **13**, 554–562 (2017).
